# Balancing the benefits and risks of colchicine use among patients with atherosclerotic cardiovascular disease: an umbrella review of meta-analyses of randomised controlled trials

**DOI:** 10.1016/j.eclinm.2025.103277

**Published:** 2025-06-05

**Authors:** Rui-Han Bao, Na Zhao, Xiao-Feng Jiang, Zi-Han Cong, Jun Liu, Long-Dan Kang, Yu-Han Chen, Jia-Nan Sun, Wei-Yi Xing, Jia-Xin Liu, Xue-Li Bai, Hai-Chen Lv, Jing-Ying Zhao, Qi-Peng Ma, Ting-Ting Gong, Qi-Jun Wu

**Affiliations:** a Department of Clinical Epidemiology, Shengjing Hospital of China Medical University, Shenyang, China; b Department of Obstetrics and Gynecology, Shengjing Hospital of China Medical University, Shenyang, China; c The First Clinical Department, China Medical University, Shenyang, China; d Department of Laboratory Medicine, The Fourth Affiliated Hospital of China Medical University, Shenyang, China; e Department of Obstetrics and Gynecology, The Fourth Affiliated Hospital of China Medical University, Shenyang, China; f Department of Cardiology, The Fourth Affiliated Hospital of China Medical University, Shenyang, China; g Department of Ophthalmology, The First Affiliated Hospital of China Medical University, Shenyang, China; h Department of Epidemiology, School of Public Health, China Medical University, Shenyang, China; i Department of Cardiology, The First Affiliated Hospital of Dalian Medical University, Dalian, China; j Department of Pediatrics, Shengjing Hospital of China Medical University, Shenyang, China; k NHC Key Laboratory of Advanced Reproductive Medicine and Fertility (China Medical University), National Health Commission, Shenyang, China

**Keywords:** Colchicine, Atherosclerotic cardiovascular disease, Umbrella review, Randomised controlled trials

## Abstract

**Background:**

The risks and benefits of colchicine use among patients with atherosclerotic cardiovascular disease (ASCVD) have been widely reported. Our umbrella review aimed to systematically analyse and synthesise the available causal evidence on the therapeutic effects and safety profile of colchicine in patients with ASCVD.

**Methods:**

We searched PubMed, Embase, Web of Science, and Cochrane Library from database inception to December 4, 2024, to identify systematic reviews and meta-analyses of randomised controlled trials (RCTs) investigating colchicine use among patients with ASCVD. The primary endpoints were physical adverse events, MACEs, and CV disorder. The quality of systematic reviews was evaluated using A Measurement Tool to Assess Systematic Reviews (AMSTAR), and the certainty of evidence was assessed using the Grading of Recommendations, Assessment, Development, and Evaluations (GRADE) system. Risk of bias was evaluated using Cochrane Systematic Review criteria. Inconsistency was flagged if heterogeneity (*I*^*2*^) exceeded 50%, or 75% without explanation. Publication bias was detected via funnel plot asymmetry or Egger’s test. Subgroup analyses were done according to colchicine dose, treatment duration, geographical region, participant age and disease of patients. This study is registered with PROSPERO (CRD 42025631311).

**Findings:**

This review analysed 47 unique outcomes from 48 systematic reviews and meta-analyses of RCTs. Among 271 associations, 95 were supported by high-certainty evidence. For primary outcomes, compared with placebo treatment, colchicine exhibited therapeutic efficacy in coronary heart disease [relative risk (RR): 0.73, 95% confidence interval (CI): 0.64–0.83, *I*^*2*^: 0%] and acute coronary syndromes (OR: 0.72, 95% CI: 0.58–0.89, *I*^*2*^: 0%), demonstrating secondary prevention benefits in major adverse cardiovascular events (RR: 0.56, 95% CI: 0.47–0.67, *I*^*2*^: 0%). Common adverse effects included gastrointestinal reactions and hepatic toxicity, with dose-dependent associations of drug discontinuation (RR: 4.63, 95% CI: 2.06–10.38, *I*^*2*^: 36%). Evidence of publication bias was identified in 18 of 271 tested associations. Subgroup analyses indicated that optimal clinical application required consideration of patient age, geographic variance in pharmacogenomics, and protocol adjustments (recommended dose ≤0.5 mg/day for more than a month) to balance efficacy–risk profiles.

**Interpretation:**

High-certainty evidence supported the therapeutic and secondary preventive benefits of colchicine in patients with ASCVD when dose and duration were appropriately controlled. Future studies could focus on identifying ASCVD subgroups with pharmacogenomic and ethnographic predictors of colchicine response heterogeneity to guide precision therapy.

**Funding:**

This work was supported by the 10.13039/501100018617Liaoning Revitalization Talents Program, Outstanding Youth Scientific Talent Project of Dalian, and Outstanding Scientific Fund of 10.13039/501100015226Shengjing Hospital.

**Research in context**

**Evidence before this study**

We searched PubMed, Embase, Web of Science, and the Cochrane Database of Systematic Reviews from inception to December 4, 2024, for meta-analyses of colchicine use among patients with atherosclerotic cardiovascular disease (ASCVD). This search yielded inconsistent findings among studies and even guideline discordance, leading to doubts over the validity of the claimed efficacy of colchicine use among patients with ASCVD, and the potential influence of biases (such as publication bias) in the literature. Inconsistencies in the results of various studies have raised concerns regarding the validity of the purported effectiveness of colchicine use among patients with ASCVD. Previous umbrella reviews have only investigated whether colchicine can reduce mortality in patients with COVID-19 clinical syndrome, without focusing on the application of this medicine in the cardiovascular (CV) field.

**Added value of this study**

To address the shortcomings of previous studies on this topic, we conducted a comprehensive updated umbrella review encompassing a thorough analysis of randomised controlled trials and focusing on diverse CV-related outcomes. Additionally, we incorporate subgroup analyses to discern potential variations in the effects of colchicine across different populations. We performed the Grading of Recommendations, Assessment, Development, and Evaluations to assess the certainty of existing evidence. We found and analysed 271 unique associations of the effect of colchicine on CV-related events. Among identified outcomes, there was high certainty of evidence that colchicine exhibited therapeutic efficacy in coronary heart disease and acute coronary syndromes, demonstrating secondary prevention benefits in acute coronary events, stroke, and major cardiovascular events (MACEs). Mechanistically, it attenuated inflammatory cell activation and improved revascularisation outcomes while reducing hospitalisation duration. Common adverse effects included gastrointestinal reactions and hepatic toxicity, with dose-dependent associations of drug discontinuation and non-cardiovascular mortality. Subgroup analyses indicated optimal clinical application required consideration of patient age, geographic variance in pharmacogenomics, and protocol adjustments (recommended dose ≤0.5 mg/day for more than a month) to balance efficacy–risk profiles.

**Implications of all the available evidence**

Our findings, supported by high certainty of evidence, provide insights for clinicians and scientists in helping them provide high-evidence-level recommendations when considering colchicine for patients with ASCVD. Current evidence supports the adoption of conservative dosing regimens with extended treatment duration to optimise the risk–benefit ratio, though therapeutic protocols should remain adaptable to evolving clinical contexts. Additional research is necessary to thoroughly assess the impact of colchicine on various health outcomes and elucidate the underlying mechanisms involved.

## Introduction

Atherosclerotic cardiovascular disease (ASCVD) accounts for 32% of global mortality (17.9 million annual deaths) and affects 340 million individuals worldwide, with a $1.1 trillion economic burden in 2022.^1^ Despite standard therapies, 30% of patients experience major adverse cardiovascular events (MACEs) within 10 years.^2^ Beyond traditional risk factors, systemic low-grade inflammation is now well-established as a key driver of plaque progression and destabilisation.^3^, ^4^, ^5^ However, the clinical efficacy of conventional anti-inflammatory agents such as methotrexate remains debated,^6^ while persistent residual inflammation under statin therapy is associated with an elevated risk of recurrence,^7^ necessitating the exploration of alternative pharmacological agents with anti-inflammatory properties.

Colchicine, a low-cost anti-inflammatory agent traditionally used for Familial Mediterranean Fever, Behçet’s disease, and gout,^8^ is now gaining traction in the cardiovascular (CV) domain. Growing evidence supports its efficacy in coronary heart disease (CHD) through ASCVD mechanisms.^9^ Mechanistically, its benefits are linked to NLRP3 (NOD-like receptor thermal protein domain associated protein 3) inflammasome inhibition,^10^ macrophage polarisation (M1 suppression/M2 promotion),^11^^,^^12^ and reduced leukocyte-platelet aggregation.^13^

Multiple RCT meta-analyses have assessed colchicine’s effects on ASCVD outcomes and recurrent event risks. However, heterogeneity in dosing (0.5–1.0 mg/day), populations, and endpoints contributes to inconsistent findings—some studies report reduced CV mortality,^14^, ^15^, ^16^ others show no mortality benefit. Guideline discordance persists.^17^^,^^18^ The 2021 ESC Guidelines^19^ conditionally recommend low-dose colchicine (IIb/B) for high-risk chronic coronary syndromes, supported by LoDoCo2/COLCOT. Conversely, the 2023 ACC/AHA Guidelines^20^ advise against routine use (III/B-R) due to safety concerns (COPS trial). The 2025 AHA/ACS Statement^21^ permits short-term use (IIb/B-NR) in select post-ACS patients (CLEAR-SYNERGY). Discrepancies exist in target populations (chronic vs. acute) and safety monitoring, while consensus cautions against use in severe renal impairment or with CYP3A4/P-gp inhibitors. Divergent recommendations highlight unmet needs for biomarker-stratified trials and long-term safety data.

Moreover, beyond the debated efficacy of colchicine across diverse clinical scenarios, its relatively narrow therapeutic window often results in frequent side effects and toxicity.^22^^,^^23^ As such, this underscores the necessity for a highly individualised treatment approach. The effects of colchicine are dose-dependent, with most adverse reactions being reversible upon dose reduction or discontinuation.^24^ Currently, most clinically recommended low-dose regimens (0.5–0.6 mg)^19^^,^^25^^,^^26^ exhibit significant variability in treatment duration, often extending beyond one month.^26^ Additionally, dosage formulations differ by geographic region, with 0.6-mg tablets available in the United States and 0.5-mg tablets in Australia. The absence of comparative data on the efficacy and toxicity profiles of these formulations suggests that the differences in preparation strength are likely driven by commercial manufacturing considerations.^26^ Age-related prescribing disparities exist, with evidence favoring use in patients >65 years old.^27^ Additionally, the heterogeneity of the disease suffered by patients has its own exploratory value, especially the impact of surgery on medication needs to be discussed in depth. We therefore analysed dosage,^25^^,^^26^ duration,^26^ geography,^26^ age,^27^ and disease of patients to refine clinical applicability.

Umbrella review, a useful tool, aimed at providing a relatively comprehensive understanding of published systematic reviews with meta-analyses on a specific topic recently.^28^ Considering the great controversy of the evidence, we conducted a umbrella review. Our primary aim was to comprehensively summarise the risks and benefits of colchicine use among patients with ASCVD based on meta-analyses of RCTs. The secondary aim was to identify the population that would benefit the most from colchicine use, thus supporting evidence-based clinical decision-making.

## Methods

### Protocol registration

The current umbrella review was conducted following the Preferred Reporting Items for Systematic Reviews and Meta-Analyses (PRISMA) guidelines, as detailed in Supplementary Table S1.^29^ Additionally, the protocol for this umbrella review was registered with the International Prospective Register of Systematic Reviews (PROSPERO; registration number CRD 42025631311) to mitigate the risk of duplication and reporting bias.

### Search strategy

Two investigators (R-HB and X-FJ) independently screened studies from PubMed, Embase, Web of Science, and Cochrane Library through December 4, 2024, focusing on systematic reviews and meta-analyses of RCTs evaluating colchicine use among patients with ASCVD. Data extraction followed standardised protocols and references were manually cross-checked. Discrepancies were arbitrated by a third reviewer (Q-JW), with inter-reviewer agreement quantified using Kappa statistics. Search terms are detailed in Supplementary Table S2.

### Selection criteria

Two investigators (R-HB and N-Z) independently screened the titles and abstracts for relevance and assessed the full texts of potentially eligible articles. Any discrepancies were resolved by a third investigator (Q-JW). Studies were included based on the following Population, Intervention, Comparator, Outcome, and Study design (PICOS) criteria:

(1) Population: patients with ASCVD;
(2) Intervention: colchicine use with different dose and duration;
(3) Comparison: placebo, standard treatment (e.g., conventional anti-thrombotic and anticoagulant therapy), or other alternative treatment [e.g., percutaneous coronary intervention (PCI)];
(4) Outcome: cardiovascular disease-related outcomes. The primary endpoints were 1) physical adverse events 2) MACEs and CV disorder. Secondary outcomes included mortality, stroke, revascularisation, hospitalisation and other outcomes; and
(5) Study design: systematic reviews and meta-analyses of RCTs.

The exclusion criteria were as follows: Systematic reviews without quantitative analysis; Studies that focused on observational studies, laboratory studies, or animal studies; Studies without comprehensive data (such as 95% confidence intervals [CIs], effect sizes, and the number of participants); Studies involving patients without ASCVD (such as atrial fibrillation, pericarditis, ventricular arrhythmia, etc.); or (5) Meta-analyses including fewer than three RCTs.^30^

When two or more meta-analyses examined the same association, we selected only the one that included the largest number of studies, which was usually the latest.^31^ In instances with similar datasets, we preferred the meta-analysis with the highest methodological quality.^32^ Furthermore, each analysis was assessed separately if an article performed different meta-analyses on more than one outcome or among various populations.

### Data extractions

Two trained investigators (J-L and L-DK) worked in pairs to independently collect information from each eligible study. All disagreements were resolved through consultation with a third investigator (Q-JW). We collected information on the first author, publication year, journal, number of included RCTs, heterogeneity *I*^*2*^, *P*-values, pooled effect (relative risk [RR], odds ratio [OR], hazard ratio [HR], mean difference [MD], and standard mean difference [SMD]), corresponding 95% CIs, and outcomes for each eligible meta-analysis. For primary studies in each eligible meta-analysis, we further extracted the first author, publication year, baseline health status of participants, number of cases, controls, and total participants, median age of the experimental group, the region where the study was conducted, intervention (duration and dose), the treatment in control groups, study-specific risk estimates, and 95% CIs.

### Quality assessment

Two investigators (J-L and L-DK) independently evaluated the methodological quality of included meta-analyses by using AMSTAR (A Measurement Tool to Assess Systematic Reviews). Any disagreement was resolved by a third investigator (Q-JW). AMSTAR, a reliable and valid assessment tool, comprises 11 items, including the provision of a study list, characteristics of the included studies, and the disclosure of conflicts of interest, among others. It demonstrates strong face and content validity for evaluating the methodological quality of systematic reviews, which are categorised as high (8–11), moderate (4–7), or low (0–3) quality.^33^

### Assessment of evidence credibility

Two investigators (J-L and N-Z) assessed the credibility of evidence from eligible systematic reviews and meta-analyses using the Grading of Recommendations, Assessment, Development, and Evaluations (GRADE) framework, grading evidence as high, moderate, low, or very low quality. Meta-analyses of RCTs with initially high-certainty evidence could be downgraded due to risk of bias, inconsistency, indirectness, imprecision, or publication bias.^34^ Risk of bias was evaluated using Cochrane Systematic Review criteria.^35^ Inconsistency was flagged if heterogeneity (*I*^*2*^) exceeded 50%, or 75% without explanation.^36^ Indirectness was identified for discrepancies in PICOS between original studies and meta-analyses.^37^ Imprecision was assigned if experimental or control group sizes were below 200.^38^ Publication bias was detected via funnel plot asymmetry or Egger’s test *P* < 0.05.^39^

### Data analysis

For each clinical association, we extracted data exclusively from original research classified as RCTs, systematically excluding non-RCT study designs. Associations with fewer than three qualifying RCTs were excluded to ensure statistical robustness. For associations retaining three or more RCTs, we extracted effect sizes from partial studies reported in each meta-analysis, and then recalculated the pooled effect sizes and 95% CI, using random effects models. Additionally, we calculated the *I*^*2*^ and its CIs metric for heterogeneity for each association.^40^ The regression asymmetry test was used to calculate the *P* value of Egger’s test reflecting publication bias.^41^

To conduct horizontal comparisons among different associations, for continuous outcomes, limited to SMD, we performed a transformation to a logOR based on the assumption that an underlying continuous variable produces a logistic distribution of equal standard deviation in the two intervention groups. In order to improve communication of the clinical value of findings, we have made the eOR greater than 1 for clinically harmful associations.^32^

### Sensitivity analyses and subgroup analyses

Sensitivity analyses assessed evidence credibility and association robustness under retained study conditions, excluding overlapping meta-analyses (defined as duplicate evaluations of identical outcomes^42^ and those with small samples (<25th percentile) or high bias risk.^43^^,^^44^ We further conducted sensitivity analyses by listing systematic reviews lacking quantitative synthesis to ensure that any unique outcome was considered and compare whether this conclusion was consistent with the conclusion of the main analysis. Subgroup analyses stratified by colchicine dose (≤5 mg/d; >5 mg/d),^19^^,^^25^ duration (≤1 month; >1 month),^45^ region (Asia; Europe; America; Oceania),^26^ age (<65 years old; ≥65 years old)^27^ and disease of patients (acute or stable CAD; stroke; post-operative) were conducted with GRADE evidence evaluation. Of note, while our PROSPERO protocol pre-specified primary and secondary outcomes, the subgroup analyses were exploratory and initiated post hoc. These analyses were motivated by emerging evidence from interim data suggesting potential heterogeneity in treatment effects. Given the clinical relevance of optimising colchicine use in diverse populations, we deemed it methodologically appropriate to investigate these associations as hypothesis-generating explorations. All analyses used STATA v17.

### Role of the funding source

The funder of the study had no role in study design, data collection, data analysis, data interpretation, or writing of the report.

## Results

### Literature review

**Fig. 1:**
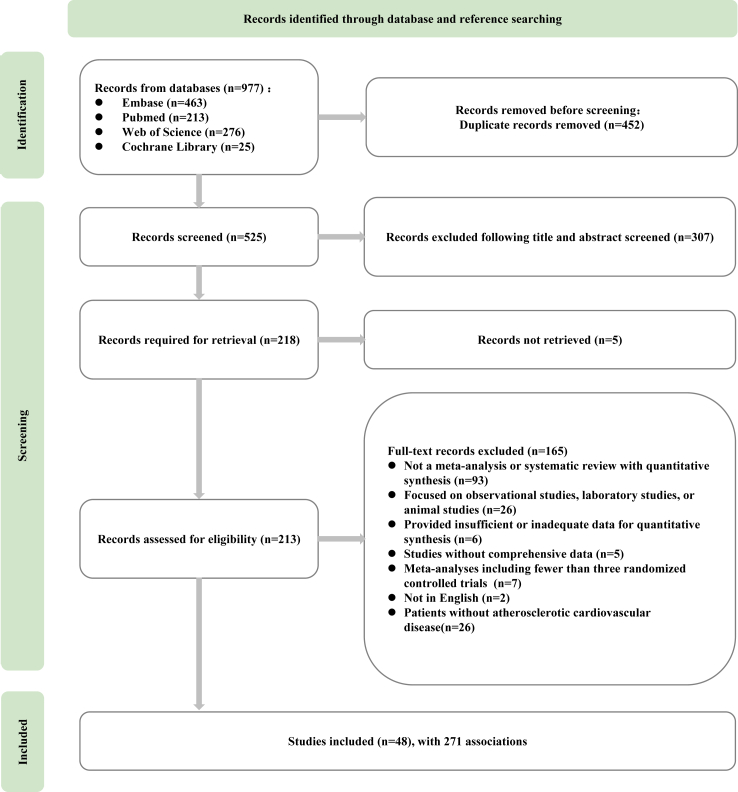
**Flow diagram of study screening and selection**.

Fig. 1 presented a PRISMA flow diagram illustrating the search strategy and study selection process. A total of 48 studies satisfied the predetermined inclusion criteria and were included in this systematic review. Study selection demonstrated a robust inter-rater agreement between two investigators, with a Kappa coefficient of 0.964. Detailed information on the excluded meta-analyses was provided in Supplementary Table S3.

### Characteristics of the included meta-analyses

The 48 eligible articles encompassed 271 associations, investigating 47 outcomes of colchicine use among patients with ASCVD (Table 1)^14^^,^^15^^,^^18^^,^^16^^,^^45^, ^46^, ^47^, ^48^, ^49^, ^50^, ^51^, ^52^, ^53^, ^54^, ^55^, ^56^, ^57^, ^58^, ^59^, ^60^, ^61^, ^62^, ^63^, ^64^, ^65^, ^66^, ^67^, ^68^, ^69^, ^70^, ^71^, ^72^, ^73^^,^^74^, ^75^, ^76^, ^77^, ^78^, ^79^, ^80^, ^81^, ^82^, ^83^, ^84^, ^85^, ^86^, ^87^, ^88^. They were published between 2010 and 2024. The median number of studies included in meta-analyses was 5 (range: 3–26) and the median number of participants was 6113 (range: 262–16,238). Overall, 271 unique meta-analytical associations were identified reporting on acceptability or tolerability of all-cause and cause-specific mortality (n = 70, 25.8%), CV disorders (n = 48, 17.7%), physical adverse events (n = 47, 17.4%), MACEs (n = 38, 14.0%), stroke (n = 28, 10.3%), revascularisation (n = 20, 7.4%), hospitalisation (n = 5, 1.9%), and other outcomes (n = 15, 5.5%).

**Table 1:** Description of 271 meta-analyses investigating the associations between colchicine intervention and cardiovascular diseases in randomised controlled trials.

Outcomes	Intervention	Intervention	Comparison	Patients	Author, year, reference	No. of RCTs	No. of cases/participants	Metric	Random effect size (95% CI)
	Dose (mg/d)	Duration (month)							
**Adverse events**									
Adverse events	0.5–1.0	0.2–1.0	Placebo	Surgery (aortic, CABG)	Wang et al., 2022^46^	3	160/334	OR	2.61 (1.28, 5.35)
Adverse events	0.5–1.0	1.0–24.0	Placebo	ACS	Diaz-Arocutipa et al., 2021^45^	6	5885/11,718	RR	1.10 (0.90, 1.35)
Adverse events	0.5–1.0	1.0–36.0	Placebo	CHD	Shrestha, 2022^47^	3	2837/5695	OR	0.99 (0.86, 1.13)
Cutaneous adverse events	0.5–2.0	0.2–28.6	Placebo	CHD	Andreis et al., 2022^16^	3	282/499	RR	0.77 (0.16, 3.69)
Diarrhea	0.5–1.0	0.2–7.1	Placebo	ACS, CCS	Ma et al., 2022^48^	5	2693/5422	RR	3.26 (1.29, 8.25)
Diarrhea	0.5–1.0	1.0–36.0	Placebo	CHD	Xiang et al., 2021^49^	5	2891/5699	RR	2.53 (1.17, 5.48)
Drug discontinuation	0.5	7.1–28.6	Placebo	CHD	Andreis et al., 2021^50^	3	5497/11,035	RR	1.26 (0.87, 1.83)
Drug discontinuation	0.5–1.0	0.2–1.0	Placebo	CHD	Papageorgiou et al., 2017^51^	5	623/1065	OR	7.59 (3.40, 16.93)
Drug discontinuation	0.5–1.0	1.0–24.0	No colchicine, placebo	ACS, CCS	Grajek et al., 2021^52^	10	6350/12,614	RR	1.60 (1.06, 2.42)
Drug discontinuation	0.5–1.0	1.0–36.0	Placebo	CHD	Verma et al., 2015^53^	3	448/803	RR	8.80 (1.44, 53.75)
Drug discontinuation	0.5–1.0	6.0–24.0	No colchicine, placebo	ACS, CCS	Grajek et al., 2021^52^	4	5806/11,594	RR	1.30 (0.94, 1.80)
Drug discontinuation	0.5–1.2	0.2–36.0	Placebo	CHD	Kofler et al., 2021^14^	11	6313/12,539	OR	1.68 (1.14, 2.48)
Drug discontinuation	0.5–2.0	0.2–28.6	Placebo	CHD	Andreis et al., 2021^50^	8	5886/11,745	RR	1.61 (1.11, 2.35)
Drug discontinuation	1	0.2–6.0	Placebo	CHD	Andreis et al., 2021^50^	4	259/513	RR	2.45 (1.27, 4.71)
Drug discontinuation	1.0–2.0	0.2–1.0	Placebo	CHD	Andreis et al., 2021^50^	3	147/291	RR	2.95 (1.10, 7.86)
Drug discontinuation	1.0–2.0	In-hospital–1.0	Placebo	Surgery (aortic, CABG)	Agarwal et al., 2023^54^	3	430/848	RR	1.33 (0.93, 1.89)
Drug discontinuation (due to adverse events)	0.5–1.0	1.0–36.0	Placebo	CHD	Verma et al., 2015^53^	3	448/803	RR	2.45 (1.27, 4.72)
Drug discontinuation (without PROBE studies)	0.5–1.0	1.0–24.0	No colchicine, placebo	ACS, CCS	Grajek et al., 2021^52^	8	6227/12,374	RR	1.34 (0.97, 1.84)
Gastrointestinal adverse events	0.5	0.2–28.6	Placebo	CHD	Andreis et al., 2022^16^	4	5607/11,230	RR	1.02 (0.92, 1.14)
Gastrointestinal adverse events	0.5–1.0	0.2–36.0	Placebo	CHD	Shrestha, 2022^47^	9	6117/12,214	OR	1.50 (1.01, 2.23)
Gastrointestinal adverse events	0.5–1.0	1.0–24.0	No colchicine, placebo	ACS, CCS	Abrantes et al., 2021^55^	9	6230/12,374	RR	1.73 (1.17, 2.56)
Gastrointestinal adverse events	0.5–1.0	1.0–36.0	Placebo	CHD	Verma et al., 2015^53^	3	448/803	RR	0.71 (0.27, 1.88)
Gastrointestinal adverse events	0.5–1.0	6.0–24.0	No colchicine, placebo	CHD	Xia et al., 2021^56^	4	5588/11,189	RR	1.05 (0.91, 1.22)
Gastrointestinal adverse events	0.5–1.0	7.1–28.6	Placebo	CHD	Sen et al., 2021^57^	3	5488/10,993	RR	1.02 (0.91, 1.13)
Gastrointestinal adverse events	0.5–1.0	7.1–28.6	Placebo	ACS, CCS, surgery (PCI)	Xu et al., 2022^58^	5	5743/11,499	RR	1.09 (0.91, 1.31)
Gastrointestinal adverse events	0.5–1.8	0.2–36.0	Placebo	ACS, CCS	Ma et al., 2022^48^	14	6704/13,399	RR	2.07 (1.45, 2.95)
Gastrointestinal adverse events	0.5–1.8	0.2–36.0	Placebo	CHD	Kofler et al., 2021^14^	12	6523/12,945	OR	2.21 (1.45, 3.36)
Gastrointestinal adverse events	0.5–1.8	0.2–36.0	Placebo	CHD	Tien et al., 2021^59^	8	3259/6420	OR	4.25 (1.18, 9.96)
Gastrointestinal adverse events	0.5–2.0	<0.1–24.0	No colchicine, placebo	CHD	Chen et al., 2023^60^	12	6693/13,273	OR	2.08 (1.39, 3.12)
Gastrointestinal adverse events	0.5–2.0	<0.1–24.0	Placebo	ACS	Bao et al., 2022^61^	9	3560/7119	RR	1.89 (1.25, 2.84)
Gastrointestinal adverse events	0.5–2.0	0.2–6.0	Placebo	ACS	Younas et al., 2025^62^	7	3232/6528	RR	1.86 (1.14, 3.02)
Gastrointestinal adverse events	0.5–2.0	0.2–24.0	Placebo, standard treatment	ACS	Diaz-Arocutipa et al., 2021^45^	5	2981/5972	RR	2.49 (0.48, 12.99)
Gastrointestinal adverse events	0.5–2.0	0.2–28.6	Placebo	CHD	Andreis et al., 2021^50^	10	6286/12,506	RR	2.00 (1.37, 2.93)
Gastrointestinal adverse events	0.5–2.0	0.2–19.6	Placebo	ACS	Zhou et al., 2023^63^	5	2642/5287	RR	2.99 (1.14, 7.82)
Gastrointestinal adverse events	0.5–2.0	0.2–36.0	Placebo	CHD	Chen et al., 2022^64^	10	3852/7609	RR	2.15 (1.40, 3.31)
Gastrointestinal adverse events	0.6–2.0	0.2–1.0	Placebo	CHD	Andreis et al., 2021^50^	4	353/691	RR	2.97 (1.69, 5.21)
Gastrointestinal adverse events	1.0–2.0	0.2–6.0	Placebo	CHD	Andreis et al., 2021^50^	4	773/1543	RR	2.72 (1.44, 5.16)
Gastrointestinal adverse events	1.0–2.0	In-hospital–6.0	No colchicine, placebo	Surgery (aortic, CABG)	Agarwal et al., 2023^54^	6	751/1529	RR	2.20 (1.38, 3.51)
Gastrointestinal adverse events	1.0–2.0	In-hospital–6.0	Placebo	Surgery (aortic, CABG)	Agarwal et al., 2023^54^	5	572/1169	RR	1.64 (1.24, 2.19)
Gastrointestinal adverse events	>1.0	0.2–28.6	Placebo	CHD	Andreis et al., 2022^16^	3	573/1062	RR	4.63 (2.06, 10.38)
Gastrointestinal adverse events (surgery < 1 m)	1.0–2.0	In-hospital–50.2	Placebo	Surgery (aortic, CABG)	Agarwal et al., 2023^54^	3	373/752	RR	2.03 (0.76, 5.46)
Gastrointestinal adverse events (surgery ≥ 1 m)	1.0–2.0	1.0–6.0	Placebo	Surgery (aortic, CABG)	Agarwal et al., 2023^54^	3	378/777	RR	2.29 (1.41, 3.72)
Hematology adverse events	0.5–2.0	0.2–28.6	Placebo	CHD	Andreis et al., 2022^16^	9	5870/11,687	RR	1.39 (0.68, 2.84)
Hepatic adverse events	0.5–2.0	0.2–28.6	Placebo	CHD	Andreis et al., 2022^16^	3	212/417	RR	1.16 (1.02, 1.32)
Postoperative adverse events	0.5–1.0	1.0–12.0	Placebo	PCI	Wei et al., 2023^65^	5	2968/6017	RR	1.55 (1.04, 2.32)
ISR	0.5–0.6	6	Placebo	ACS, surgery (PCI)	Aw et al., 2022^66^	3	230/435	RR	0.64 (0.35, 1.15)
Myalgias	0.5–2.0	0.2–28.6	Placebo	CHD	Andreis et al., 2022^16^	3	2205/4372	RR	2.88 (0.12, 69.70)
**All-cause and cause-specific mortality**									
All-cause mortality	0.5–1.0	1.0–28.6	Placebo	CHD	Akl et al., 2024^67^	8	6088/12,151	Peto OR	1.11 (0.86, 1.43)
All-cause mortality	0.5–1.0	1.0–36.0	Placebo	CHD	Verma et al., 2015^53^	3	524/951	RR	0.38 (0.14, 1.01)
All-cause mortality	0.5–1.0	1.0–36.0	Placebo	ACS, AIS, surgery (PCI)	Masson et al., 2020^68^	7	3359/6630	OR	0.85 (0.50, 1.42)
All-cause mortality	0.5–1.0	6.0–12.0	Placebo	ACS, CCS	Niu et al., 2022^69^	3	2882/5789	RR	0.99 (0.91, 1.07)
All-cause mortality	0.5–1.0	6.0–24.0	Placebo	CHD	Sattar et al., 2022^70^	5	5794/11,538	RR	1.14 (0.76, 1.69)
All-cause mortality	0.5–1.0	6.0–28.6	Placebo	CHD	Niu et al., 2022^69^	4	5644/11,311	RR	1.00 (0.91, 1.09)
All-cause mortality	0.5–1.0	6.0–36.0	Placebo	CCS	Shrestha, 2022^47^	3	3144/6250	OR	0.80 (0.32, 2.03)
All-cause mortality	0.5–1.0	7.1–36.0	Placebo	CHD, surgery (PCI)	Xu et al., 2022^58^	5	5906/11,790	RR	1.04 (0.64, 1.69)
All-cause mortality	0.5–1.0	7.1–36.0	Placebo	CHD, stroke	Fiolet et al., 2024^18^	6	7478/14,960	RR	1.09 (0.89, 1.33)
All-cause mortality	0.5–1.0	7.1–36.0	Placebo	CHD	Sen et al., 2021^57^	4	5806/11,594	RR	1.04 (0.61, 1.78)
All-cause mortality	0.5–1.2	7.1–36.0	Placebo	ACS, CCS, surgery (PCI)	Aw et al., 2022^66^	5	3198/6333	RR	1.12 (0.49, 2.58)
All-cause mortality	0.5–1.8	<0.1–24.0	Placebo	ACS	Wang et al., 2021^71^	3	2968/5940	OR	1.06 (0.70, 1.61)
All-cause mortality	0.5–1.8	<0.1–24.0	No colchicine, placebo	ACS, CCS	Grajek et al., 2021^52^	8	6382/12,666	RR	0.99 (0.68, 1.45)
All-cause mortality	0.5–1.8	<0.1–36.0	Placebo	ACS, CCS	Wang et al., 2021^71^	4	5730/11,462	OR	1.15 (0.88, 1.50)
All-cause mortality	0.5–1.8	0.2–36.0	Placebo	ACS, CCS	Ma et al., 2022^48^	13	6692/13,288	RR	1.00 (0.70, 1.42)
All-cause mortality	0.5–1.8	0.2–36.0	Placebo	CHD	Kofler et al., 2021^14^	13	6602/13,098	OR	0.96 (0.65, 1.41)
All-cause mortality	0.5–2.0	<0.1	Placebo	Surgery (PCI)	Fu et al., 2021^72^	4	2773/5482	OR	0.89 (0.60, 1.32)
All-cause mortality	0.5–2.0	<0.1–12.0	Placebo	CHD	Liao et al., 2021^73^	5	3169/6277	OR	0.93 (0.63, 1.36)
All-cause mortality	0.5–2.0	<0.1–24.0	Placebo	ACS	Bao et al., 2022^61^	5	3062/6138	RR	1.25 (0.70, 2.24)
All-cause mortality	0.5–2.0	0.2–6.0	Placebo	ACS	Younas et al., 2025^62^	8	3361/6757	RR	1.00 (0.72, 1.39)
All-cause mortality	0.5–2.0	0.2–22.0	Placebo	ACS	Shrestha, 2022^47^	3	2839/5691	OR	1.65 (0.44, 6.23)
All-cause mortality	0.5–2.0	0.2–24.0	Placebo, standard treatment	ACS	Diaz-Arocutipa et al., 2021^45^	5	2981/5972	RR	1.06 (0.61, 1.85)
All-cause mortality	0.5–2.0	0.2–28.6	Placebo	CHD	Andreis et al., 2022^16^	11	6501/12,869	RR	1.01 (0.71, 1.43)
All-cause mortality	0.5–2.0	0.2–36.0	Placebo	CHD	Chen et al., 2022^64^	11	6650/13,200	OR	1.09 (0.85, 1.40)
All-cause mortality	0.5–2.0	0.2–36.0	Placebo	CHD	Xiang et al., 2021^49^	8	5776/11,463	RR	0.70 (0.61, 0.80)
All-cause mortality	0.5–2.0	1.0–28.6	Placebo	CHD	Bytyçi et al., 2022^74^	9	6294/12,552	RR	1.05 (0.71, 1.53)
All-cause mortality	1	1.0–3.0	Placebo	ACS	Ullah et al., 2021^75^	3	2874/5762	RR	1.62 (0.45, 5.88)
All-cause mortality (excluding smaller studies)	0.5–2.0	0.2–22.0	Placebo	ACS	Shrestha, 2022^47^	3	2839/5691	OR	2.21 (0.29, 17.16)
All-cause mortality (excluding the outlier study)	0.5–1.0	6.0–36.0	Placebo	CCS	Shrestha, 2022^47^	3	3144/6250	OR	0.40 (0.14, 1.19)
All-cause mortality (excluding the outlier study)	0.5–2.0	0.2–22.0	Placebo	ACS	Shrestha, 2022^47^	3	2839/5691	OR	3.44 (0.42, 27.90)
All-cause mortality (fixed-effect model)	0.5–1.0	6.0–36.0	Placebo	CCS	Shrestha, 2022^47^	3	3144/6250	OR	1.09 (0.79, 1.50)
All-cause mortality (fixed-effect model)	0.5–2.0	0.2–22.0	Placebo	ACS	Shrestha, 2022^47^	3	2839/5691	OR	1.14 (0.76, 1.70)
All-cause mortality (follow-up time < 6 m)	0.5–2.0	<0.1–0.2	Placebo	CHD	Chen et al., 2023^60^	3	354/691	OR	0.63 (0.22, 1.81)
All-cause mortality (follow-up time ≥6 m)	0.5–1.0	6.0–24.0	No colchicine, placebo	CHD	Chen et al., 2023^60^	6	6045/11,996	OR	1.09 (0.85, 1.40)
All-cause mortality (postoperative)	0.5–1.0	6.0–12.0	Placebo	Surgery (PCI)	Wei et al., 2023^65^	3	2862/5736	RR	1.14 (0.77, 1.68)
All-cause mortality (preoperative, postoperative)	0.5–2.0	<1.0–6.0	Placebo	Surgery (PCI)	Wei et al., 2023^65^	3	321/586	RR	0.75 (0.32, 1.74)
CV mortality	0.5–1.0	0.2–36.0	Placebo	CHD	Kofler et al., 2021^14^	9	6165/12,302	OR	0.82 (0.46, 0.90)
CV mortality	0.5–1.0	1.0–22.0	Placebo	ACS	Ullah et al., 2021^75^	5	3031/6064	OR	0.91 (0.53, 1.59)
CV mortality	0.5–1.0	1.0–24.0	Placebo, standard treatment	ACS	Diaz-Arocutipa et al., 2021^45^	4	2904/5821	RR	0.91 (0.52, 1.61)
CV mortality	0.5–1.0	1.0–28.6	Placebo	CHD	Akl et al., 2024^67^	8	6088/12,151	Peto OR	0.78 (0.53, 1.16)
CV mortality	0.5–1.0	1.0–36.0	Placebo	ACS, CCS	Ma et al., 2022^48^	9	6286/12,548	RR	0.91 (0.49, 1.68)
CV mortality	0.5–1.0	1.0–36.0	Placebo	ACS, AIS, surgery (PCI)	Masson et al., 2020^68^	6	2909/5794	OR	0.42 (0.07, 2.61)
CV mortality	0.5–1.0	1.0–36.0	No colchicine, placebo	ACS, CCS	Grajek et al., 2021^52^	6	3284/6547	RR	0.80 (0.55, 1.18)
CV mortality	0.5–1.0	6.0–12.0	Placebo	ACS, CCS	Niu et al., 2022^69^	3	2882/5789	RR	0.92 (0.82, 1.03)
CV mortality	0.5–1.0	6.0–24.0	Placebo	CHD	Sattar et al., 2022^70^	4	5662/11,341	RR	0.85 (0.57, 1.27)
CV mortality	0.5–1.0	6.0–24.0	No colchicine, placebo	ACS, CCS	Abrantes et al., 2021^55^	6	6051/12,016	RR	0.79 (0.53, 1.18)
CV mortality	0.5–1.0	6.0–24.0	No colchicine, placebo	CHD	Xia et al., 2021^56^	5	5906/11,790	RR	0.79 (0.43, 1.45)
CV mortality	0.5–1.0	6.0–28.6	Placebo	CHD	Niu et al., 2022^69^	4	5644/11,311	RR	0.94 (0.86, 1.02)
CV mortality	0.5–1.0	6.0–36.0	No colchicine, placebo	ACS, CCS	Grajek et al., 2021^52^	5	5906/11,790	RR	0.80 (0.53, 1.22)
CV mortality	0.5–1.0	6.0–36.0	Placebo	CCS	Shrestha, 2022^47^	3	3144/6250	OR	0.42 (0.07, 2.48)
CV mortality	0.5–1.0	7.1–28.6	Placebo	ACS, CCS	Samuel et al., 2021^76^	3	5524/11,062	HR	0.82 (0.46, 1.18)
CV mortality	0.5–1.0	7.1–36.0	Placebo	CHD	Sen et al., 2021^57^	4	5806/11,594	RR	0.71 (0.48, 1.05)
CV mortality	0.5–1.0	7.1–36.0	Placebo	ACS, CCS, surgery (PCI)	Xu et al., 2022^58^	6	5940/11,862	RR	0.74 (0.58, 0.95)
CV mortality	0.5–1.0	7.1–36.0	Placebo	CHD, stroke	Fiolet et al., 2024^18^	6	7478/14,960	RR	0.89 (0.65, 1.23)
CV mortality	0.5–1.0	12.0–24.0	No colchicine, placebo	CHD	Chen et al., 2023^60^	4	5806/11,569	OR	0.77 (0.52, 1.15)
CV mortality	0.5–1.8	<0.1–7.1	Placebo	ACS	Wang et al., 2021^71^	3	2968/5940	OR	0.91 (0.52, 1.61)
CV mortality	0.5–1.8	<0.1–36.0	Placebo	ACS, CCS	Wang et al., 2021^71^	4	5730/11,462	OR	0.86 (0.57, 1.29)
CV mortality	0.5–1.8	1.0–36.0	Placebo	CHD	Chen et al., 2022^64^	9	6446/12,852	OR	0.80 (0.54, 1.18)
CV mortality	0.5–2.0	0.2–24.0	Placebo	ACS	Bao et al., 2022^61^	4	2959/5940	RR	0.99 (0.58, 1.69)
CV mortality	0.5–2.0	0.2–28.6	Placebo	CHD	Andreis et al., 2022^16^	9	6255/12,389	RR	0.73 (0.45, 1.21)
CV mortality	0.5–2.0	1.0–28.6	Placebo	CHD	Bytyçi et al., 2022^74^	7	6048/12,071	RR	0.75 (0.40, 1.43)
CV mortality (fixed-effect model)	0.5–1.0	6.0–36.0	Placebo	ACS	Shrestha, 2022^47^	3	3144/6250	OR	0.57 (0.34, 0.98)
Non-CV mortality	0.5–1.0	1.0–28.6	Placebo	CHD	Akl et al., 2024^67^	8	6088/12,151	Peto OR	1.54 (1.10, 2.15)
Non-CV mortality	0.5–1.0	1.0–36.0	Placebo	ACS, CCS	Ma et al., 2022^48^	7	6065/12,107	RR	1.32 (0.91, 1.92)
Non-CV mortality	0.5–1.0	7.1–28.6	Placebo	CHD	Andreis et al., 2021^50^	3	5524/11,062	RR	1.43 (0.93, 2.19)
Non-CV mortality	0.5–1.0	7.1–36.0	Placebo	CHD	Sen et al., 2021^57^	4	5806/11,594	RR	1.53 (1.10, 2.14)
Non-CV mortality	0.5–1.0	7.1–36.0	Placebo	CHD, stroke	Fiolet et al., 2024^18^	5	7375/14,738	RR	1.26 (0.97, 1.64)
Non-CV mortality	0.5–1.0	7.1–36.0	Placebo	ACS, CCS	Xu et al., 2022^58^	4	5624/11,258	RR	1.34 (0.90, 2.00)
Non-CV mortality	0.5–1.0	12.0–24.0	No colchicine, placebo	CHD	Xia et al., 2021^56^	4	5806/11,594	RR	1.50 (0.93, 2.40)
Non-CV mortality	0.5–1.8	1.0–36.0	Placebo	CHD	Chen et al., 2022^64^	9	6446/12,852	OR	1.38 (1.00, 1.92)
**Cardiovascular disorders**									
ACS	0.5–1.0	1.0–22.0	Placebo	ACS	Ullah et al., 2021^75^	6	3142/6288	OR	0.80 (0.51, 1.24)
ACS	0.5–1.0	1.0–24.0	No colchicine, placebo	ACS, CCS	Abrantes et al., 2021^55^	7	5988/11,955	RR	0.62 (0.44, 0.89)
ACS	0.5–1.0	1.0–24.0	No colchicine, placebo	CHD	Chen et al., 2023^60^	5	2961/5937	OR	0.70 (0.53, 0.92)
ACS	0.5–1.0	1.0–24.0	Placebo	ACS, CCS	Akl et al., 2024^67^	5	2944/5901	OR	0.72 (0.58, 0.89)
ACS	0.5–1.0	1.0–36.0	Placebo	CHD	Shrestha, 2022^47^	3	714/1401	OR	0.39 (0.24, 0.64)
ACS	0.5–1.8	1.0–22.7	Placebo	ACS	Alberto et al., 2021^77^	5	NA/5654	HR	0.77 (0.56, 1.05)
ACS (new)	0.5–1.8	<0.1–24.0	No colchicine, placebo	CHD	Chen et al., 2023^60^	8	6194/12,386	OR	0.68 (0.57, 0.81)
ACS or UA	0.5–1.0	1.0–36.0	Placebo	CHD	Shrestha, 2022^47^	3	714/1401	OR	0.43 (0.23, 0.83)
ACS or UA (fixed-effect model)	0.5–1.0	1.0–36.0	Placebo	CHD	Shrestha, 2022^47^	3	714/1401	OR	0.42 (0.26, 0.67)
CHD	0.5–1.0	6.0–36.0	Placebo	CHD	Xu et al., 2022^58^	3	5524/11,602	RR	0.56 (0.31, 0.99)
CHD	0.5–1.2	<0.1–36.0	Placebo	CCS	Casula et al., 2022^78^	3	3250/6636	RR	0.62 (0.39, 0.98)
CHD	0.5–2.0	0.2–28.6	Placebo	CHD	Andreis et al., 2021^50^	8	6050/12,059	RR	0.73 (0.64, 0.83)
CCS	0.5–1.8	12.0–36.0	Placebo	CCS	Alberto et al., 2021^77^	3	NA/6256	HR	0.51 (0.32, 0.81)
Cardiac arrest	0.5–2.0	1.0–3.0	Placebo	ACS	Younas et al., 2025^62^	3	2929/5896	RR	0.81 (0.33, 1.95)
MI	0.5–1.0	1.0–24.0	Placebo, standard treatment	CHD	Sattar et al., 2022^70^	5	5829/11,638	RR	0.75 (0.62, 0.90)
MI	0.5–1.0	1.0–36.0	Placebo	CHD	Akl et al., 2024^67^	6	5948/11,875	OR	0.75 (0.62, 0.91)
MI	0.5–1.0	1.0–36.0	Placebo	ACS, AIS, surgery (PCI)	Masson et al., 2020^68^	5	2809/5598	OR	0.45 (0.13, 1.60)
MI	0.5–1.0	6.0–12.0	Placebo	ACS	Niu et al., 2022^69^	3	2882/5789	RR	0.92 (0.85, 0.99)
MI	0.5–1.0	6.0–28.6	Placebo	CHD	Niu et al., 2022^69^	4	5644/11,311	RR	0.90 (0.84, 0.96)
MI	0.5–1.0	7.1–28.6	Placebo	CHD	Niu et al., 2022^69^	3	5524/11,062	RR	0.85 (0.77, 0.94)
MI	0.5–1.0	7.1–36.0	Placebo	ACS, CHD	Samuel et al., 2021^76^	4	5774/11,594	HR	0.62 (0.36, 0.88)
MI	0.5–1.0	7.1–36.0	Placebo	CHD	Al-Atta et al., 2021^79^	4	5806/11,594	RR	0.65 (0.46, 0.93)
MI	0.5–1.0	12.0–24.0	No colchicine, placebo	CHD	Xia et al., 2021^56^	4	5806/11,594	RR	0.73 (0.55, 0.98)
MI	0.5–1.0	12.0–36.0	No colchicine, placebo	ACS, CCS	Grajek et al., 2021^52^	4	5806/11,594	RR	0.72 (0.52, 1.00)
MI	0.5–1.2	1.0–28.6	Placebo	CHD	Andreis et al., 2022^16^	7	6154/12,275	RR	0.76 (0.61, 0.96)
MI	0.5–1.8	<0.1–24.0	No colchicine, placebo	ACS, CCS	Grajek et al., 2021^52^	7	6146/12,262	RR	0.73 (0.57, 0.95)
MI	0.5–1.8	0.2–36.0	Placebo	ACS, CCS	Ma et al., 2022^48^	9	6392/12,752	RR	0.60 (0.43, 0.83)
MI	0.5–1.8	1.0–36.0	Placebo	CHD	Chen et al., 2022^64^	7	6306/12,576	RR	0.77 (0.64, 0.92)
MI	0.5–2.0	0.2–19.6	Placebo	ACS	Zhou et al., 2023^63^	4	2601/5205	RR	0.88 (0.67, 1.15)
MI (postoperative)	0.5–1.0	1.0–7.1	Placebo	PCI	Wei et al., 2023^65^	3	2508/5026	RR	0.89 (0.67, 1.17)
MI (acute)	0.5–1.0	1.0–28.6	Placebo	CHD	Shrestha, 2022^47^	3	5151/10,311	OR	0.80 (0.65, 0.98)
MI (acute)	0.5–1.0	1.0–36.0	Placebo	CHD	Xiang et al., 2021^49^	4	5394/10,804	RR	0.77 (0.64, 0.94)
MI (recurrent)	0.5–1.0	1.0–24.0	Placebo, standard treatment	ACS	Diaz-Arocutipa et al., 2021^45^	4	2904/5821	RR	0.87 (0.62, 1.22)
MI (recurrent)	0.5–1.2	1.0–36.0	Placebo	CHD	Bytyçi et al., 2022^74^	7	6154/12,275	RR	0.78 (0.65, 0.93)
MI (recurrent)	0.5–1.8	<0.1–7.1	Placebo	ACS	Wang et al., 2021^71^	4	2968/5940	OR	0.86 (0.66, 1.13)
MI (recurrent)	0.5–1.8	<0.1–36.0	Placebo	ACS, CCS	Wang et al., 2021^71^	5	6012/11,994	OR	0.76 (0.63, 0.92)
MI (recurrent)	0.5–2.0	<0.1–24.0	Placebo	ACS	Bao et al., 2022^61^	7	3220/6447	RR	0.75 (0.49, 1.14)
MI (recurrent)	0.5–2.0	1.0–6.0	Placebo	ACS	Younas et al., 2025^62^	6	3185/6415	RR	0.78 (0.57, 1.06)
POAF	0.5–1.0	<0.1–3.0	Placebo	Surgery (aortic, CABG)	Ge et al., 2022^80^	5	588/1165	OR	0.70 (0.56, 0.86)
POAF	0.5–1.0	0.2–1.0	Placebo	Surgery (aortic, CABG)	Wang et al., 2022^46^	4	268/550	OR	0.59 (0.39, 0.89)
POAF	0.5–2.0	0.3–out-hospital	Placebo	Surgery (CABG)	Kirov et al., 2024^81^	5	415/839	RR	0.54 (0.40, 0.73)
POAF	0.5–2.0	<3.0	Placebo	Surgery (aortic, CABG, MR)	Zhao et al., 2022^82^	5	488/968	RR	0.40 (0.25, 0.65)
POAF	0.5–2.0	NA	Placebo	CHD	Teo et al., 2021^83^	4	439/868	RR	0.64 (0.48, 0.86)
POAF	1.0–2.0	In-hospital–6.0	Placebo	Surgery (aortic, CABG)	Agarwal et al., 2023^54^	8	931/1876	RR	0.70 (0.59, 0.82)
POAF (<1 m)	1.0–2.0	In-hospital–0.3	Placebo	Surgery (aortic, CABG, MR)	Zhao et al., 2022^82^	5	488/968	RR	0.65 (0.49, 0.86)
POAF (<1 m)	1.0–2.0	In-hospital–50.2	Placebo	Surgery (aortic, CABG, MR)	Agarwal et al., 2023^54^	5	552/1108	RR	0.63 (0.49, 0.81)
POAF (≥1 m)	1.0–2.0	1.0–6.0	Placebo	Surgery (aortic, CABG)	Agarwal et al., 2023^54^	3	378/777	RR	0.74 (0.56, 0.99)
POAF (no history of AF)	1.0–2.0	In-hospital–50.2	Placebo	Surgery (aortic, CABG)	Agarwal et al., 2023^54^	4	444/892	RR	0.68 (0.51, 0.90)
**Hospitalization**									
Hospitalization	0.5–1.0	1.0–28.6	Placebo	ACS, CCS	Abrantes et al., 2021^55^	4	5643/11,299	RR	0.76 (0.53, 1.10)
Hospitalization	0.5–2.0	1.0–28.6	Placebo	CHD	Bytyçi et al., 2022^74^	7	555/1112	RR	0.32 (0.12, 0.87)
Hospitalization urgency	0.5–1.0	1	Placebo	ACS	Younas et al., 2025^62^	3	2939/5928	RR	0.46 (0.31, 0.68)
Rehospitalization	0.5–1.0	1.0–22.0	Placebo	CHD	Shrestha, 2022^47^	4	2909/5838	OR	0.64 (0.20, 2.06)
Rehospitalisation (excluding the outlier study)	0.5–1.0	1.0–22.0	Placebo	CHD	Shrestha, 2022^47^	3	543/1093	OR	0.30 (0.11, 0.84)
**MACEs**									
ACS, AF, mortality, revascularization and stroke	0.5–2.0	<0.1–6.0	No colchicine, placebo	Surgery (CABG, PCI)	Chen et al., 2023^60^	7	649/1244	OR	0.68 (0.44, 1.05)
ACS, CHD, post-angioplasty and stroke	0.5–1.0	1.0–36.0	Placebo	CHD	Verma et al., 2015^53^	4	560/1025	RR	0.40 (0.25, 0.65)
Adverse cardiovascular events^a^	0.5–2.0	1.0–6.0	Placebo	ACS	Younas et al., 2025^62^	7	3184/6505	RR	0.75 (0.60, 0.94)
All-cause mortality, cardiac arrest, ISR, MI, stent thrombosis and stroke	0.5–1.0	1.0–12.0	Placebo	Surgery (PCI)	Wei et al., 2023^65^	5	3004/6017	RR	0.70 (0.58, 0.84)
All-cause mortality, cardiac arrest, ISR, MI, stent thrombosis and stroke	0.5–2.0	0.2–6.0	Placebo	Surgery (PCI)	Wei et al., 2023^65^	5	583/1099	RR	0.67 (0.50, 0.89)
All-cause mortality and stroke (post surgery)	0.5–2.0	0.2–3.0	Placebo	Surgery (aortic, CABG, MR)	Ge et al., 2022^80^	6	730/1449	RR	0.86 (0.46, 1.60)
All-cause mortality, CV mortality, MI and stroke	0.5–1.0	6.0–24.0	No colchicine, placebo	ACS, CCS	Abrantes et al., 2021^55^	6	5869/11,718	RR	0.65 (0.49, 0.86)
All-cause mortality, CV mortality, MI and stroke	0.5–1.0	6.0–24.0	No colchicine, placebo	CHD	Xia et al., 2021^56^	5	5906/11,790	RR	0.65 (0.52, 0.82)
All-cause mortality, CV mortality, recurrent MI and stroke	0.5–1.2	1.0–36.0	Placebo	CHD	Bytyçi et al., 2022^74^	7	6154/12,275	RR	0.67 (0.55, 0.83)
All-cause mortality, ACS, CV mortality, revascularization and stroke	0.5–1.0	12.0–36.0	Placebo	CHD	Shrestha, 2022^47^	4	5806/11,594	OR	0.63 (0.48, 0.83)
All-cause mortality, CV mortality and stroke	0.5–1.2	1.0–12.0	Placebo	CHD	Aw et al., 2022^66^	7	3347/6660	RR	0.73 (0.61, 0.87)
All-cause mortality, HF, MI, revascularization and stroke	0.5	1.0–19.6	Placebo	ACS	Zhou et al., 2023^63^	3	2638/5290	RR	0.56 (0.47, 0.67)
All-cause mortality, HF, MI, revascularization and stroke	0.5–2.0	0.2–19.6	Placebo	ACS	Zhou et al., 2023^63^	5	2762/5526	RR	0.56 (0.48, 0.67)
All-cause mortality, HF, MI, revascularization and stroke	0.5–2.0	<12.0	Placebo	ACS	Zhou et al., 2023^63^	3	235/460	RR	0.56 (0.31, 1.03)
All-cause mortality, HF, MI, revascularization and stroke (≤3 d)	0.5–2.0	0.2–19.6	Placebo	ACS	Zhou et al., 2023^63^	5	993/1964	RR	0.58 (0.44, 0.78)
All-cause mortality, recurrent MI and stroke	0.5–1.8	<0.1–36.0	Placebo	ACS, CCS	Wang et al., 2021^71^	4	5730/11,462	OR	0.81 (0.70, 0.95)
All-cause mortality, recurrent MI, revascularization and stroke	0.5	12.0–36.0	Placebo	ACS, CCS	Wang et al., 2021^71^	3	5524/11,062	OR	0.76 (0.67, 0.85)
CV mortality, coronary revascularization, MI and stroke	0.5	7.1–28.6	Placebo	Stroke, TIA	Fiolet et al., 2024^18^	3	1730/3473	RR	0.84 (0.70, 1.02)
CV mortality, coronary revascularization, MI and stroke	0.5	7.1–36.0	Placebo	CHD	Condello et al., 2021^84^	3	5410/10,799	RR	0.73 (0.50, 1.06)
CV mortality, coronary revascularization, MI and stroke	0.5	7.1–36.0	Placebo	ACS, CCS, surgery (PCI)	Xu et al., 2022^58^	5	5806/12,134	RR	0.64 (0.50, 0.82)
CV mortality, coronary revascularization, MI and stroke	0.5–1.0	1.0–36.0	Placebo	CHD	Teo et al., 2021^83^	3	NA/NA	HR	0.71 (0.62, 0.82)
CV mortality, coronary revascularization, MI and stroke	0.5–1.0	12.0–36.0	Placebo	CHD	Masson et al., 2021^85^	4	5804/11,592	HR	0.80 (0.69, 0.93)
CV mortality, coronary revascularization, MI and stroke	0.5–1.0	7.1–28.6	Placebo	ACS, CCS	Samuel et al., 2021^76^	3	5524/11,062	HR	0.68 (0.54, 0.81)
CV mortality, coronary revascularization, MI and stroke	0.5–1.0	7.1–36.0	Placebo	ACS, CCS, surgery (PCI)	Xu et al., 2022^58^	7	6048/12,071	RR	0.64 (0.51, 0.80)
CV mortality, coronary revascularization, MI and stroke	0.5–1.0	7.1–36.0	Placebo	CHD, stroke	Fiolet et al., 2024^18^	5	7375/14,738	RR	0.73 (0.65, 0.81)
CV mortality, coronary revascularization, MI and stroke	0.5–1.0	7.1–36.0	Placebo	CHD	Al-Atta et al., 2021^79^	4	5524/11,062	RR	0.65 (0.51, 0.83)
CV mortality, MI and revascularization	0.5–1.8	<0.1–12.0	Placebo	CHD	Liao et al., 2021^73^	3	2968/5940	OR	0.74 (0.59, 0.92)
CV mortality, MI and stroke	≤0.5	1.0–28.6	Placebo	CHD	Akl et al., 2024^67^	5	5925/11,831	Peto OR	0.67 (0.58, 0.77)
CV mortality, MI and stroke	>0.5	1.0–6.0	Placebo	CHD	Akl et al., 2024^67^	3	163/320	Peto OR	0.84 (0.32, 2.23)
CV mortality, MI and stroke	0.5–1.0	0.2–36.0	Placebo	ACS, CCS	Ma et al., 2022^48^	7	6067/12,115	RR	0.54 (0.38, 0.77)
CV mortality, MI and stroke	0.5–1.0	1	Placebo	CHD	Akl et al., 2024^67^	3	182/361	Peto OR	0.57 (0.14, 2.33)
CV mortality, MI and stroke	0.5–1.0	>1.0	Placebo	CHD	Akl et al., 2024^67^	5	5906/11,790	Peto OR	0.67 (0.59, 0.77)
CV mortality, MI and stroke	0.5–1.0	1.0–36.0	Placebo	ACS, CCS	Akl et al., 2024^67^	6	5948/11,875	OR	0.70 (0.60, 0.83)
CV mortality, MI and stroke	0.5–1.0	12.0–24.0	No colchicine, placebo	Atherosclerosis	Fiolet et al., 2021^15^	4	5806/11,594	RR	0.75 (0.61, 0.92)
CV mortality, MI, PCI-related myocardial injury, revascularization and stroke	0.5–1.8	<0.1–24.0	No colchicine, placebo	ACS, CCS	Grajek et al., 2021^52^	5	6012/11,994	RR	0.70 (0.55, 0.88)
Major CV events^b^	0.5–1.0	7.1–36.0	Placebo	CHD with diabetes	Kuzemczak et al., 2021^86^	4	1102/2278	RR	0.73 (0.57, 0.93)
Major CV events^b^	0.5–1.0	7.1–36.0	Placebo	CHD without diabetes	Kuzemczak et al., 2021^86^	4	1102/2278	RR	0.69 (0.59, 0.82)
Major adverse CV and cerebrovascular events	0.5–2.0	0.2–28.6	Placebo	CHD	Andreis et al., 2022^16^	11	6501/12,869	RR	0.67 (0.56, 0.80)
**Revascularization**									
Revascularization	0.5–1.0	1.0–28.6	Placebo	CHD	Teo et al., 2021^83^	3	NA/NA	HR	0.59 (0.38, 0.91)
Revascularization	0.5–1.0	1.0–36.0	Placebo	CHD	Xiang et al., 2021^49^	4	5191/10,391	RR	0.70 (0.58, 0.86)
Revascularization	0.5–1.0	1.0–36.0	Placebo	ACS, CCS	Akl et al., 2024^67^	4	5806/11,594	OR	0.67 (0.58, 0.82)
Revascularization	0.5–1.0	1.0–36.0	No colchicine, placebo	ACS, CCS	Grajek et al., 2021^52^	6	6017/12,014	RR	0.57 (0.41, 0.80)
Revascularization	0.5–1.0	6.0–24.0	No colchicine, placebo	Atherosclerosis	Fiolet et al., 2021^15^	5	5918/11,816	RR	0.77 (0.66, 0.90)
Revascularization	0.5–1.0	6.0–24.0	No colchicine, placebo	CHD	Chen et al., 2023^60^	7	5988/11,986	OR	0.65 (0.53, 0.78)
Revascularization	0.5–1.0	6.0–28.6	Placebo	CHD	Chen et al., 2022^64^	4	5624/11,258	RR	0.77 (0.66, 0.89)
Revascularization	0.5–1.0	6.0–28.6	Placebo	ACS, CCS	Abrantes et al., 2021^55^	4	5636/11,284	RR	0.61 (0.42, 0.89)
Revascularization	0.5–1.0	6.0–36.0	No colchicine, Placebo	ACS, CCS	Grajek et al., 2021^52^	5	5906/11,790	RR	0.57 (0.40, 0.82)
Revascularization	0.5–1.0	7.1–28.6	Placebo	ACS, CCS	Samuel et al., 2021^76^	3	5524/11,062	HR	0.56 (0.30, 0.82)
Revascularization	0.5–1.0	7.1–28.6	Placebo	CHD	Niu et al., 2022^69^	3	5524/11,062	RR	0.74 (0.66, 0.84)
Revascularization	0.5–1.0	7.1–36.0	Placebo	ACS, CCS, surgery (PCI)	Xu et al., 2022^58^	5	5906/11,790	RR	0.48 (0.28, 0.83)
Revascularization	0.5–1.0	12.0–24.0	Placebo	CHD	Sattar et al., 2022^70^	3	5524/11,062	RR	0.58 (0.37, 0.92)
Revascularization	0.5–1.0	12.0–36.0	Placebo	ACS, CCS	Wang et al., 2021^71^	3	5524/11,062	OR	0.68 (0.56, 0.83)
Revascularization	0.5–1.8	<0.1–24.0	Placebo	ACS	Bao et al., 2022^61^	3	2865/5738	RR	0.46 (0.29, 0.73)
Revascularization	0.5–1.8	<0.1–28.6	Placebo	CHD	Ma et al., 2022^48^	5	5830/11,658	RR	0.61 (0.42, 0.89)
Repeat vessel revascularization	0.5	6.0–12.0	Placebo	CHD	Aw et al., 2022^66^	4	2892/5862	RR	0.47 (0.31, 0.72)
Restenosis after PCI	1.0–1.2	6	Placebo	Surgery (PCI)	Tien et al., 2021^59^	3	260/453	OR	0.46 (0.23, 0.92)
Revascularization (fixed-effect model)	0.5–1.0	1.0–36.0	Placebo	CHD	Shrestha, 2022^47^	4	5624/11,258	OR	0.63 (0.52, 0.76)
Stent thrombosis	0.5	1.0–12.0	Placebo	CHD	Aw et al., 2022^66^	3	545/1122	RR	0.50 (0.25, 0.98)
**Stroke**									
Ischaemic stroke	0.5–1.0	1.0–36.0	Placebo	CHD	Xiang et al., 2021^49^	5	5550/11,075	RR	0.49 (0.30, 0.79)
Ischaemic stroke	0.5–1.0	7.1–36.0	Placebo	ACS, CCS	Samuel et al., 2021^76^	4	5774/11,594	HR	0.38 (0.13, 0.63)
Ischaemic stroke	0.5–1.0	7.1–36.0	Placebo	CHD, stroke	Fiolet et al., 2024^18^	5	7375/14,738	RR	0.73 (0.58, 0.90)
Ischaemic stroke	0.5–1.8	1.0–36.0	Placebo	CHD	Chen et al., 2022^64^	5	6172/12,308	RR	0.47 (0.30, 0.76)
Non-cardio-embolic ischaemic stroke	0.5–1.0	1.0–36.0	Placebo	CHD	Shrestha, 2022^47^	6	5942/11,864	OR	0.48 (0.30, 0.76)
Stroke	0.5	19.6–36	Placebo	ACS, CCS	Ullah et al., 2021^75^	3	5410/10,799	RR	0.43 (0.19, 0.96)
Stroke	0.5	22.6–36.0	Placebo	CCS	Katsanos et al., 2021^87^	3	5410/10,799	RR	0.43 (0.21, 0.89)
Stroke	0.5–1.0	0.2–36.0	Placebo	ACS, CCS	Ma et al., 2022^48^	6	5947/11,866	RR	0.50 (0.31, 0.80)
Stroke	0.5–1.0	1.0–12.0	Placebo	ACS, AIS	Katsanos et al., 2021^87^	3	536/1071	RR	0.55 (0.15, 2.05)
Stroke	0.5–1.0	1.0–24.0	No colchicine, placebo	ACS, CCS	Abrantes et al., 2021^55^	6	5958/11,896	RR	0.48 (0.30, 0.78)
Stroke	0.5–1.0	1.0–36.0	Placebo	CHD	Katsanos et al., 2020^88^	4	2788/5552	RR	0.31 (0.13, 0.71)
Stroke	0.5–1.0	1.0–36.0	Placebo	ACS, CHD	Akl et al., 2024^67^	8	6088/12,151	OR	0.47 (0.30, 0.74)
Stroke	0.5–1.0	1.0–36.0	Placebo	ACS, AIS, CCS	Masson et al., 2020^68^	5	2809/5598	OR	0.33 (0.15, 0.72)
Stroke	0.5–1.0	6.0–12.0	Placebo	Surgery (PCI)	Wei et al., 2023^65^	3	2862/5736	RR	0.33 (0.15, 0.73)
Stroke	0.5–1.0	7.1–28.6	Placebo	CHD	Niu et al., 2022^69^	3	5524/11,062	RR	0.96 (0.85, 1.09)
Stroke	0.5–1.0	7.1–36.0	Placebo	ACS, CCS	Xu et al., 2022^58^	5	5906/11,790	RR	0.82 (0.55, 1.22)
Stroke	0.5–1.0	7.1–36.0	Placebo	CHD	Al-Atta et al., 2021^79^	4	5806/11,594	RR	0.48 (0.29, 0.78)
Stroke	0.5–1.0	12.0–24.0	Placebo	CHD	Sattar et al., 2022^70^	4	5806/11,594	RR	0.46 (0.28, 0.74)
Stroke	0.5–1.0	12.0–36.0	No colchicine, placebo	ACS, CCS	Grajek et al., 2021^52^	5	5906/11,790	RR	0.47 (0.28, 0.81)
Stroke	0.5–1.0	12.0–36.0	Placebo	ACS, CCS	Ullah et al., 2021^75^	4	5806/11,594	RR	0.43 (0.22, 0.83)
Stroke	0.5–1.2	7.1–36.0	Placebo	CHD	Aw et al., 2022^66^	7	6152/12,270	RR	0.50 (0.31, 0.81)
Stroke	0.5–1.8	<0.1–24.0	Placebo	ACS	Wang et al., 2021^71^	3	2968/5940	OR	0.32 (0.14, 0.72)
Stroke	0.5–1.8	<0.1–24.0	No colchicine, placebo	CHD	Chen et al., 2023^60^	7	5882/12,000	OR	0.51 (0.32, 0.82)
Stroke	0.5–1.8	<0.1–36.0	Placebo	ACS, CCS	Wang et al., 2021^71^	5	6012/11,994	OR	0.48 (0.30, 0.79)
Stroke	0.5–2.0	<0.1–24.0	Placebo	ACS	Bao et al., 2022^61^	5	3248/6395	RR	0.39 (0.18, 0.81)
Stroke	0.5–2.0	0.2–28.6	Placebo	CHD	Andreis et al., 2022^16^	7	6152/12,270	RR	0.48 (0.30, 0.77)
Stroke	0.5–2.0	1.0–3.0	Placebo	ACS	Younas et al., 2025^62^	4	3030/6088	RR	0.45 (0.17, 1.19)
Stroke	0.5–2.0	1.0–28.6	Placebo	CHD	Bytyçi et al., 2022^74^	7	6165/12,223	RR	0.47 (0.29, 0.76)
**Other outcomes**									
Cancer	0.5–1.0	7.1–28.6	Placebo	CHD	Sen et al., 2021^57^	3	5488/10,993	RR	0.98 (0.80, 1.21)
CRP	0.5–1.0	0.2–19.6	Placebo	ACS	Zhou et al., 2023^63^	4	2654/5322	MD	0.39 (−0.78, 1.57)
CRP	0.5–2.0	0.2–12.0	Placebo	ACS	Zhou et al., 2023^63^	5	357/709	MD	−7.69 (−19.50, 4.11)
CRP	0.5–2.0	0.2–19.6	Placebo	ACS	Zhou et al., 2023^63^	8	1161/1815	MD	−0.21 (−1.01, 0.59)
Hs-CRP	0.5–1.0	1.0–24.0	No colchicine, placebo	ACS, CCS	Grajek et al., 2021^52^	6	5942/11,864	RR	0.99 (0.68, 1.45)
Hs-CRP	0.5–2.0	0.2–6.0	Placebo	ACS	Younas et al., 2025^62^	6	403/793	MD	−0.87 (−1.80, 0.06)
Hs-CRP	0.5–2.0	0.2–24.0	Placebo, standard treatment	ACS	Diaz-Arocutipa et al., 2021^45^	4	227/449	MD	−1.95 (−12.88, 8.98)
Infection	0.5–1.0	24	Placebo	CHD	Chen et al., 2023^60^	3	5143/10,318	OR	1.42 (0.82, 2.46)
Infection	0.5–2.0	NA	Placebo	CHD	Teo et al., 2021^83^	3	5163/10,338	RR	1.37 (0.82, 2.29)
Leukocytes	0.5–1.0	1.0–19.6	Placebo	ACS	Zhou et al., 2023^63^	3	1178/2344	SMD	−0.05 (−0.13, 0.03)
LVEF	0.5–2.0	0.2–12.0	Placebo	ACS	Zhou et al., 2023^63^	3	318/633	MD	3.18 (0.10, 6.27)
Neutrophils	0.5–1.0	0.2–19.6	Placebo	ACS	Zhou et al., 2023^63^	4	1255/2492	SMD	−0.07 (−0.15, 0.01)
Pneumonia	0.5–1.0	7.1–28.6	Placebo	CHD	Sen et al., 2021^57^	3	5488/10,993	RR	1.47 (0.57, 3.79)
Pneumonia	0.5–1.0	12.0–24.0	Placebo	Atherosclerosis	Fiolet et al., 2021^15^	3	5488/10,993	RR	1.67 (0.58, 4.77)
Pneumonia	0.5–1.0	12.0–24.0	Placebo	CHD	Chen et al., 2023^60^	3	5488/10,993	OR	1.55 (0.58, 4.18)

ACS, acute coronary syndrome; AF, atrial fibrillation; AIS, acute ischaemic stroke; CABG, coronary artery bypass grafting; CCS, chronic coronary syndromes; CHD, coronary heart disease; CI, confidence interval; CRP, C-reactive protein; CV, cardiovascular; d, day; HF, heart failure; HR, hazard ratio; Hs-CRP, high-sensitive C-reactive protein; ISR, in-stent restenosis; LVEF, left ventricular ejection fraction; m, month; MACEs, major adverse cardiac events; MD, mean difference; MI, myocardial infarction; MR, myocardial revascularization; NA, not available; No., number; OR, odds ratio; PCI, percutaneous coronary intervention; POAF, postoperative atrial fibrillation; PROBE, Prospective; Randomized, Open-label, Blinded Endpoint; RCTs, randomised controlled trials; RR, risk ratio; SMD, standardized mean difference; TIA, transient ischaemic attack; UA, unstable angina.

a Adverse cardiovascular events: ACS, CV mortality, HF, MI, resuscitated cardiac arrest, stroke, UA, urgent hospitalization for angina and ventricular arrhythmias.

b Major CV events: ACS, out-of-hospital cardiac arrest, CV mortality, resuscitated cardiac arrest, MI, stroke, or urgent hospitalization for angina, leading to coronary revascularization, ischaemic stroke, or ischemia-driven coronary revascularization.

### Methodological quality of meta-analyses

Based on the quality assessment of included studies performed using the AMSTAR questionnaire (Supplementary Fig. S1), 30 studies (63%) were classified as high quality, 17 studies (35%) as moderate quality, and 1 study (2%) as low quality. The AMSTAR assessment was downgraded mainly because of the absence of a comprehensive list outlining the included and excluded studies.

### Summary of associations

**Fig. 2:**
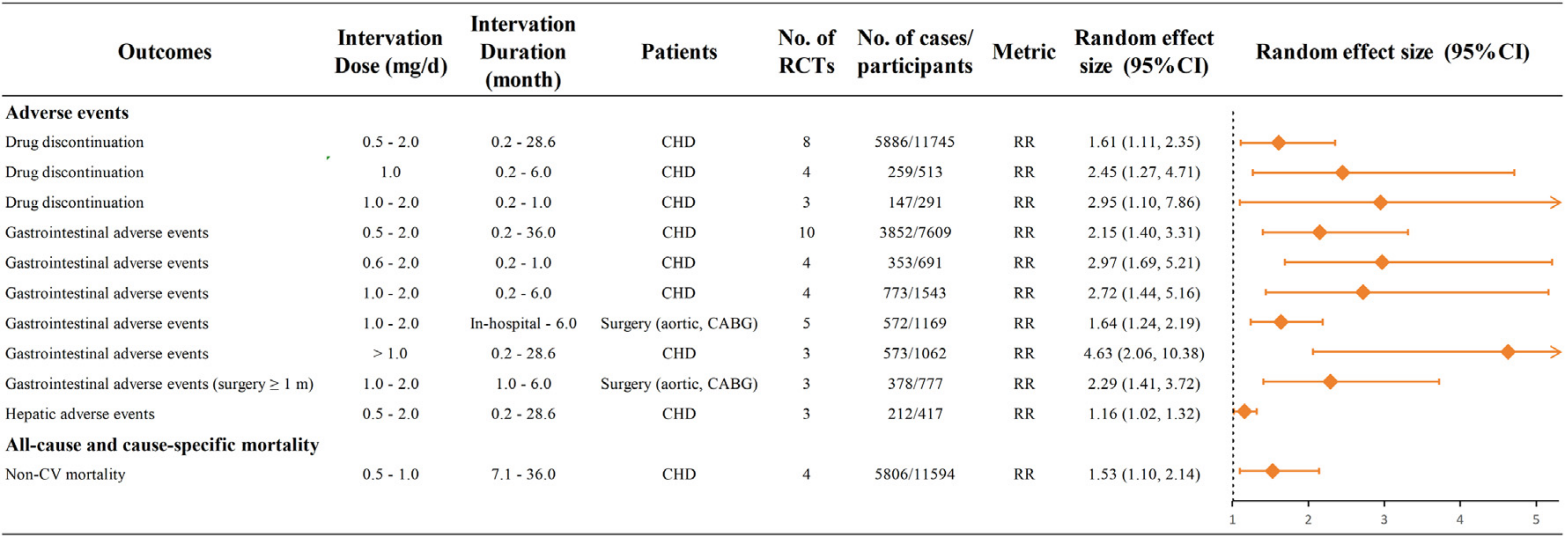
**Summary of associations between the efficacy of colchicine use in different atherosclerotic cardiovascular disease patients and different outcomes, with statistical significance.** High certainty evidence according to Grading of Recommendations, Assessment, Development and Evaluations (GRADE), from randomised controlled trials on outcomes of colchicine based medications in people with all-cause and cause-specific mortality, cardiovascular disorders, hospitalization, MACEs, revascularization, stroke and other outcomes. Only associations for which an equivalent effect size was available are displayed. ACS, acute coronary syndrome; CABG, coronary artery bypass grafting; CCS, chronic coronary syndromes; CHD, coronary heart disease; HF, heart failure; HR, hazard ratio; MACEs, major adverse cardiovascular events; MI, myocardial infarction; OR, odds ratio; POAF, postoperative atrial fibrillation; PCI, percutaneous coronary intervention; RR, relative risk; UA, unstable angina. ∗ Adverse cardiovascular events: ACS, CV mortality, HF, MI, resuscitated cardiac arrest, stroke, UA, urgent hospitalization for angina and ventricular arrhythmias. ^†^ Major CV events: ACS, out-of-hospital cardiac arrest, CV mortality, resuscitated cardiac arrest, MI, stroke, or urgent hospitalization for angina, leading to coronary revascularization, ischemic stroke, or ischemia-driven coronary revascularization.

**Fig. 3:**
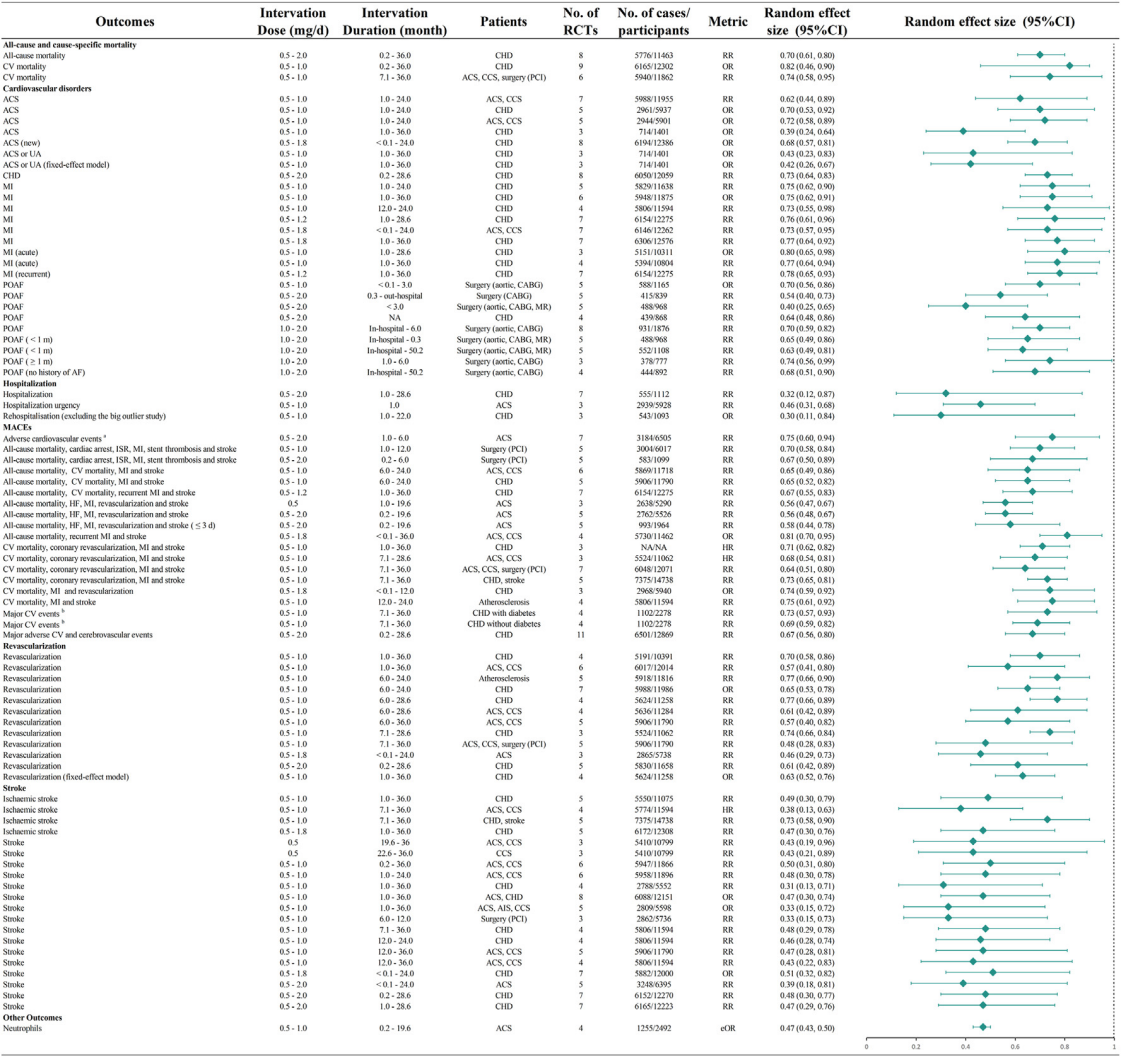
**Summary of associations between the efficacy of colchicine use in different atherosclerotic cardiovascular disease patients and different outcomes, with statistical significance.** High certainty evidence according to Grading of Recommendations, Assessment, Development and Evaluations (GRADE), from randomised controlled trials on outcomes of colchicine based medications in people with adverse events, all-cause and cause-specific mortality. Only associations for which an equivalent effect size was available are displayed. CABG, coronary artery bypass grafting; CHD, coronary heart disease; CV, cardiovascular; eOR, equivalent odds ratio; m, month; RR, relative risk.

**Fig. 4:**
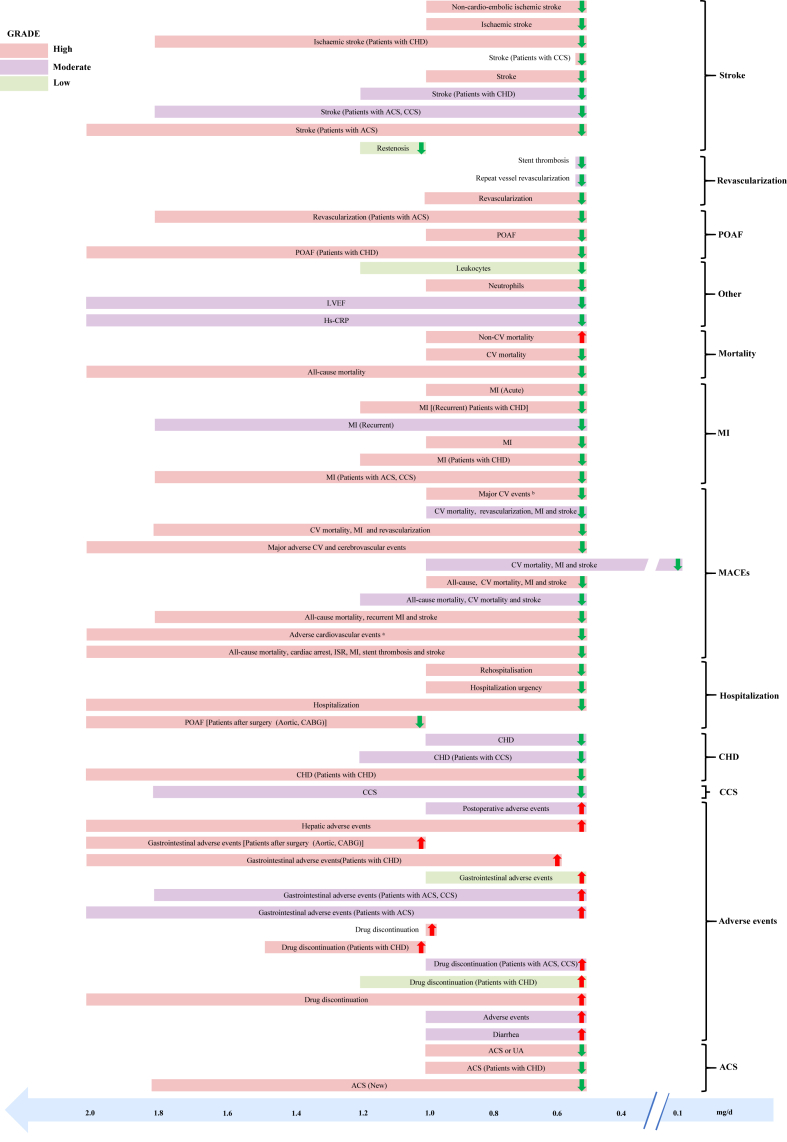
**Results across the dose of colchicine use, with statistical significance.** The horizontal bar length represents the measurement of colchicine, the green down arrow represents the protective factor, and the red up arrow represents the adverse factor. ACS, acute coronary syndrome; CABG, coronary artery bypass grafting; CCS, chronic coronary syndromes; CHD, coronary heart disease; GRADE, Grading of Recommendations, Assessment, Development and Evaluation; MACEs, major adverse cardiovascular events; MI, myocardial infarction; POAF, postoperative atrial fibrillation; PCI, percutaneous coronary intervention; UA, unstable angina. ∗ Adverse cardiovascular events: ACS, CV mortality, HF, MI, resuscitated cardiac arrest, stroke, UA, urgent hospitalization for angina and ventricular arrhythmias. ^†^ Major CV events: ACS, out-of-hospital cardiac arrest, CV mortality, resuscitated cardiac arrest, MI, stroke, or urgent hospitalization for angina, leading to coronary revascularization, ischemic stroke, or ischemia-driven coronary revascularization.

Using a predefined grading system for evidence quality, high-certainty evidence was observed in 19 (11%) of studies evaluating primary endpoints (adverse reactions), 57 (34%) of studies examining drug benefit outcomes (CV disorders and MACEs), and 93 (55%) of studies addressing secondary endpoints (Supplementary Table S4). Figs. 2 and 3 respectively showed the relationship between the efficacy and safety of colchicine use among different patients with ASCVD in different outcomes. Fig. 4 depicted the results across the dose of colchicine use. In the following sections, we mainly described the risks and benefits of colchicine treatment and prevention effects among different populations, mainly focusing on the associations with statistical significance.

### Associations of primary outcomes

#### CV disorders

In this analysis, a total of 37 associations were identified, with 26 (70%) classified as high and 11 (30%) as moderate certainty of evidence, with all associations indicating protective effect. Notably, the use of colchicine compared with placebo demonstrated therapeutic benefits with high-level certainty for acute coronary syndrome (ACS) (n = 2) and CHD (n = 1). In patients with ACS, a modest difference in efficacy was observed between the control group receiving no colchicine or placebo (RR: 0.62, 95% CI: 0.44–0.89, *I*^*2*^: 48%) and the group treated with placebo alone (OR: 0.72, 95% CI: 0.58–0.89, *I*^*2*^: 0%). For patients with CHD, compared with placebo group, a therapeutic benefit was demonstrated (RR: 0.73, 95% CI: 0.64–0.83, *I*^*2*^: 0%) with colchicine doses ranging from 0.5 to 2.0 mg daily over a wide treatment duration span (0.2–28.6 months). Furthermore, among CHD patients, colchicine demonstrated secondary preventive effects in contrast to the placebo-treated participants among ACS or unstable angina (UA) (n = 7), myocardial infarction (MI) (n = 6), and acute myocardial infarction (AMI) (n = 3). In postoperative patients, colchicine also significantly lowered the risk of postoperative atrial fibrillation (POAF) (n = 9).

#### MACEs

Among the 31 associations identified for this outcome, 19 (61%), 11 (36%), and 1 (3%) were supported by high, moderate, and low-certainty evidence, respectively, with all associations demonstrating a protective effect. The definition of MACEs varied across studies, introducing some heterogeneity. In this study, MACEs primarily encompassed all-cause mortality, cardiac arrest, in-stent re-stenosis, MI, stent thrombosis, stroke, urgent hospitalisation for angina, and ventricular arrhythmia, among others. Notably, for patients with ACS, when benchmarked against placebo recipients, the maximum protective effects (RR: 0.56, 95% CI: 0.47–0.67, *I*^*2*^: 0%) were observed for all-cause mortality, heart failure, MI, revascularisation, and stroke when a daily dose of 0.5 mg was administered over a wide duration range (1–19.6 months).

#### Adverse events

Among the 29 associations identified for this outcome, 10 (34%), 11 (38%), and 8 (28%) were supported by high, moderate, and low-certainty evidence, respectively, with all associations indicating harmful effects. The adverse events supported by high-certainty evidence included gastrointestinal complications (n = 6), drug discontinuation (n = 3), and hepatic adverse events (n = 1). In patients with CHD, the use of colchicine was associated with an increased risk of gastrointestinal adverse reactions, withdrawal responses, and liver-related adverse effects. Specifically, when the daily dosage exceeded 1 mg and the treatment duration was relatively prolonged (ranging from 0.2 to 28.6 months), the drug exhibited the most pronounced side effects (RR: 4.63, 95% CI: 2.06–10.38, *I*^*2*^: 36%) compared with placebo group. For postoperative patients, only gastrointestinal adverse reactions were increased, with the highest risk observed when the treatment extended beyond one month after surgery (RR: 2.29, 95% CI: 1.41–3.72, *I*^*2*^: 0%), when benchmarked against placebo recipients.

### Associations of secondary outcomes

#### Stroke

Among the 24 associations identified for this outcome, 20 (83%) and 4 (17%) demonstrated high and moderate-certainty, respectively, with all associations indicating protective effects. For patients with CHD, including ACS and chronic coronary syndrome (CCS), especially for those after PCI, colchicine could lower the risk of stroke. In patients with CHD, colchicine showed superiority over placebo (RR: 0.31, 95% CI: 0.13–0.71, *I*^*2*^: 0%) when the dose was 0.5 to 1.0 mg per day for a long time span (1–36 months).

#### Revascularisation

Among the 20 associations identified for this outcome, 12 (60%), 6 (30%), and 2 (10%) were supported by high, moderate, and low-certainty evidence, respectively, with all associations demonstrating protective effects. Colchicine exhibited a protective effect on vascular remodeling in patients with ACS, CCS, CHD, and atherosclerosis. The most significant protective effect compared with placebo group was observed at daily doses of 0.5–1.8 mg over a treatment duration ranging from less than 0.1–24 months (RR: 0.46, 95% CI: 0.29–0.73, *I*^*2*^: 1%).

#### Hospitalisation

Three associations were identified, all rated as high-certainty evidence and showing protective effects. After excluding the big outlier trial based on study weight, the risk of readmission was minimised in patients with CHD (OR: 0.30, 95% CI: 0.11–0.84, *I*^*2*^: 0%).

#### All-cause and cause-specific mortality

For this outcome, a total of six associations were evaluated, with four (67%) demonstrating high-certainty evidence and two (33%) classified as moderate-certainty evidence. Colchicine significantly reduced the risk of all-cause mortality (RR: 0.70, 95% CI: 0.61–0.80, *I*^*2*^: 3%) and CV mortality (OR: 0.82, 95% CI: 0.46–0.90, *I*^*2*^: 41%) in patients with CHD, when compared with placebo group. Additionally, it lowered the risk of CV mortality in patients following percutaneous coronary intervention (RR: 0.74, 95% CI: 0.58–0.95, *I*^*2*^: 17.9%). However, colchicine was associated with an increased risk of non-CV mortality (RR: 1.53, 95% CI: 1.10–2.14, *I*^*2*^: 23%) in patients with CHD.

#### Other outcomes

For the remaining outcomes, four associations demonstrated statistical significance, with only one association classified as high-certainty evidence. In patients with ACS, a daily colchicine dose of 0.5–1.0 mg exhibited a protective effect over placebo group on neutrophil count (SMD: 0.07, 95% CI: 0.15–0.01, *I*^*2*^: 47%) across a wide treatment duration range (0.2 to 19.6 months).

### Sensitivity analyses

We conducted sensitivity analyses to assess the robustness of the primary analysis. First, excluding meta-analyses with overlapping data (21 associations removed) reduced evidence certainty for all-cause mortality from high to moderate in two studies, while increasing certainty for all-cause and gastrointestinal adverse events from moderate to high in two studies (Supplementary Table S5).

Second, removing studies with small sample sizes (<25th percentile) excluded 87 associations, with 61 remaining consistent with the primary analysis. High-certainty evidence was downgraded to moderate for eight associations, while discontinuation and all-cause mortality outcomes were downgraded to very low certainty (Supplementary Table S6).

Third, excluding studies with higher risk of bias (90 associations excluded) showed unchanged results for 74 associations. High-certainty evidence was downgraded to moderate for nine associations, and one gastrointestinal adverse effect outcome was downgraded to very low certainty (Supplementary Table S7).

Fourth, we further listed findings from such studies to ensure that any unique cancer was considered. We found that colchicine could reduce the risk of coronary heart disease (CHD), stroke, and other diseases in the population with ASCVD, which was consistent with our findings and did not affect the final conclusion (Supplementary Table S8).

### Subgroup analyses based on dose

Subgroup analyses stratified by dose revealed that lower doses (≤0.5 mg/d) were supported by high-certainty evidence for their association with reductions in stroke (n = 6), MACEs (n = 3), MI (n = 3), revascularisation (n = 3), ACS (n = 1), and all-cause mortality (n = 1). Lower doses were also linked to an increased risk of gastrointestinal adverse events (n = 1) and non-CV mortality (n = 1). Conversely, subgroup analyses for higher doses (>0.5 mg/d) demonstrated high-certainty evidence for their association with reductions in MACEs (n = 2), revascularisation (n = 2), CHD (n = 1), and POAF (n = 1). Additionally, higher doses were associated with an elevated risk of drug discontinuation (n = 3) and gastrointestinal adverse events (n = 1). A comparison between the two subgroups indicated that higher doses were associated with more severe adverse reactions but did not expand the therapeutic range or enhance the drug’s efficacy. There was no significant difference compared to the main analysis (Supplementary Table S9).

### Subgroup analyses based on use duration

Subgroup analyses based on treatment duration demonstrated that shorter duration (≤1 month) were supported by high-certainty evidence for reducing POAF (n = 1) and MACEs (n = 1). However, shorter duration were also associated with an increased risk of gastrointestinal adverse events (n = 9) and drug discontinuation (n = 1). In contrast, longer duration (>1 month) of colchicine use showed high-certainty evidence for reductions in stroke (n = 11), MACEs (n = 4), MI (n = 4), revascularisation (n = 3), CHD (n = 2), CV mortality (n = 1), and ACS (n = 1). Overall, the subgroup analysis of treatment duration suggested that prolonged colchicine use may offer greater preventive and therapeutic benefits across a wider range of conditions without a significant increase in adverse reactions. This subgroup analysis was consistent with the main analysis, showing no significant differences in comparison (Supplementary Table S10).

### Subgroup analyses based on geographic region

Regarding regional subgroup analyses, the majority of studies were carried out in the Oceania and Europe. In the Americas, three studies demonstrated that colchicine reduced all-cause mortality risk, while in Asia, one study confirmed its association with gastrointestinal adverse events. In Oceania, particularly in Australia, colchicine use lowered the risk of CV disorders (n = 14), stroke (n = 3), revascularisation (n = 3), MACEs (n = 3), and hospitalisation (n = 2). In Europe, colchicine use was linked to a reduction in POAF but a significant increase in side effects, including gastrointestinal adverse events (n = 3) and drug discontinuation (n = 1). All these associations were supported by high-certainty evidence and were statistically significant. In comparing different regions, this study found that colchicine exhibited higher safety and a broader range of indications in Oceania, whereas its safety profile was less favorable in Europe. In addition, when compared with the main analysis, the drug had no therapeutic effect on ACS in Oceania (Supplementary Table S11).

### Subgroup analyses based on age

The results of the age-based subgroup analysis revealed high-certainty evidence supporting an association between colchicine use and reduced risks of MACEs (n = 15), CV disorders (n = 12), stroke (n = 10), revascularisation (n = 4), hospitalisation (n = 3), all-cause mortality (n = 1), and other outcomes (n = 1) among patients with ASCVD younger than 65 years. Additionally, the use of colchicine in this subgroup was also associated with elevated adverse effects (n = 4). In contrast, limited studies focused on patients with ASCVD older than 65 years, with this study indicating that colchicine use was linked to a reduced risk of CV disorders (n = 3) which was supported by high certainty of evidence. The comparison between these two subgroups partially suggests that colchicine may be more suitable for individuals under 65 years of age, offering broader therapeutic indications, albeit with corresponding side effects. Notably, the age-based subgroup analysis results were largely consistent with the main analysis findings (Supplementary Table S12).

### Subgroup analyses based on disease

Subgroup analyses stratified by patients’ disease revealed distinct risk–benefit profiles associated with colchicine therapy. In patients with coronary artery disease (CAD), robust evidence demonstrated significant risk reductions for CV disorders (n = 3), MACEs (n = 3), revascularisation procedures, stroke incidence, and high-sensitivity C-reactive protein (hs-CRP) levels. Conversely, postoperative patients exhibited divergent outcomes, with high-certainty evidence indicating elevated risks of adverse drug reactions (n = 4) alongside reduced CV risks (n = 5) and stroke prevention. Comparative analysis between subgroups demonstrated that postoperative patients experienced significantly higher rates of treatment-related adverse events without achieving superior therapeutic efficacy compared to CAD patients, underscoring the necessity for judicious risk–benefit evaluation when considering colchicine in surgical populations (Supplementary Table S13). These findings remained consistent with primary analysis results, reinforcing the clinical implications of patient stratification in therapeutic decision-making.

## Discussion

This umbrella review synthesised high-certainty evidence from interventional studies demonstrating colchicine’s secondary preventive benefits in CHD and ACS, significantly reducing risks of acute coronary events (including AMI and unstable angina), stroke, and MACEs. The intervention showed dose-dependent associations with gastrointestinal intolerance and hepatotoxicity, correlating with treatment discontinuation and excess non-CV mortality. We have observed the therapeutic efficacy of colchicine in CHD and ACS, which were all supported by high-certainty evidence. Colchicine exerted its anti-inflammatory effects through multiple mechanisms, including the inhibition of microtubule function,^89^ suppression of NLRP3 inflammasome activation,^90^ reduction in hs-CRP levels,^91^ and attenuation of platelet activity and leukocyte-platelet aggregation. Additionally, it enhanced endothelial function and inhibited the synthesis of inflammatory mediators such as prostaglandin E2, leukotriene B4, tumor necrosis factor-alpha (TNF-α), and thromboxane A2.^92^ Collectively, these mechanisms conferred substantial anti-inflammatory and CV protective effects, making colchicine a promising therapeutic option for managing CHD and atherosclerosis. Moreover, colchicine addressed persistent inflammation and reduced long-term CV complications associated with CHD treatment.^93^ Our findings provided robust evidence supporting colchicine’s efficacy in CHD management without significantly elevating the risk of MACEs. However, these conclusions might be influenced by several factors, including variations in sample sizes, differences in clinical outcome assessments, the timing of anti-inflammatory interventions, and the concurrent use of secondary prevention therapies.^94^^,^^95^ Additionally, heterogeneity among studies, stemming from differences in study design, has not been fully explained. To enhance the precision of these findings, future high-quality studies with larger sample sizes and standardised methodologies are warranted to provide more definitive estimates of the efficacy of anti-inflammatory therapies.

Our study also identified a role for colchicine in secondary prevention in patients with ASCVD. It reduced the risk of acute coronary events, including AMI, UA, and ACS. Additionally, colchicine lowered the risk of stroke and MACEs, shortened hospitalisation duration, mitigated inflammatory cell responses, and improved revascularisation outcomes. These results were supported by high levels of evidence. The potential role of colchicine in preventing postoperative AF was also supported by an umbrella review in 2022 investigating the risk and protective factors for AF after cardiac surgery and valve interventions, aligning with our findings. In addition, a study on post-cardiac injury syndrome (PCIS) demonstrated that colchicine, along with nonsteroidal anti-inflammatory drugs (NSAIDs) such as ibuprofen, was effective in preventing PCIS recurrence, consistent with our research trajectory.^96^ Persistent inflammation has been identified as a key contributor to residual CV risk in patients already receiving guideline-directed medical therapy.^97^ This has driven investigations into the potential of anti-inflammatory therapies to mitigate this risk. For instance, the CV Inflammation Reduction Trial found that methotrexate had no significant effect on either inflammatory marker levels or future CV events in patients with stable atherosclerosis.^98^ Conversely, the Canakinumab Anti-inflammatory Thrombosis Outcomes Study demonstrated that interleukin-1β (IL-1β) antagonist canakinumab reduced the rate of recurrent CV events in patients with a prior MI and elevated hs-CRP levels.^91^ However, our findings suggested that colchicine might not be universally effective in preventing all CVDs. Its clinical application should, therefore, be carefully tailored based on the specific clinical context and the heterogeneity of the underlying disease.

In the current study, the adverse effects of colchicine were mainly reflected in the gastrointestinal tract and more severe non-CV mortality, supported by the high certainty of the evidence. A recent umbrella review focused on the efficacy and safety of ischemic stroke (IS) treatment indicated that colchicine showed serious adverse events, intracranial hemorrhage, or mortality in IS patients, which was in line with our study.^99^ Non-CV mortality was generally more severe compared with gastrointestinal reactions, with infection—predominantly manifesting as pneumonia—being a major contributing factor.^100^ Some studies have suggested that increased susceptibility to infections may be attributed to the immunosuppressive properties of colchicine and its disruption of microtubule formation. Proposed mechanisms include the impairment of the nicotinamide adenine dinucleotide phosphate oxidase-superoxide system,^101^ reduced neutrophil recruitment and adhesion to inflamed tissues,^92^ decreased leukocyte adherence, and reduced emigration induced by leukotriene B4.^102^ Although the precise mechanism underlying the association between colchicine use and the development of infections remained unclear, early monitoring and the implementation of preventive antibiotic therapy might help address this therapeutic challenge.^94^

Furthermore, gastrointestinal intolerance to colchicine was transient.^103^ In case of gastrointestinal symptoms when starting colchicine, it might be useful to temporarily split the daily dose, reduce dairy intake, and add spasmolytic and antidiarrheal agents to the treatment.^103^ The gastrointestinal effects of colchicine were not surprising, considering its mechanism of action. Since colchicine was a microtubule-disassembling agent, it directly affected mitosis and cell replication processes.^104^ Cells with a high turnover rate, such as those of the gastrointestinal tract, are highly susceptible to colchicine effects.^105^^,^^106^ Additionally, our findings showed that anti-inflammatory agents conferred no significant risk of new incident cancers. This might indicate that drug-induced immunosuppression is not sufficient to promote tumorigenesis.

Regarding subgroup analyses, this study suggested that the age and geographic region of patients should be carefully considered when using colchicine, with a preference for patients with ASCVD under 65 years of age and those in Oceania. However, this conclusion may be influenced by the number of available studies, endpoint effects, local policies, or national drug guidelines, necessitating further investigation. In terms of dosage and treatment duration, we recommended a low-dose, long-term regimen, although adjustments should be made based on individual clinical circumstances.

This comprehensive systematic evaluation establishes a novel evidence synthesis paradigm for colchicine in ASCVD, incorporating 48 rigorously conducted meta-analyses encompassing 47 distinct clinical endpoints across the disease continuum–from ACS to stroke secondary prevention with several strengths. Key strengths include: 1) Adhering to established systematic review and evidence synthesis methods, explored potential bias sources. Previous studies focused solely on colchicine’s impact on COVID-19 mortality,^107^ without addressing its role in CVD therapeutics and prevention; 2) Multiple dimensions (efficacy dimension, safety dimension, population subgroup, and region subgroup), the risk–benefit ratio change under different doses and courses is visualised; 3) Exclusive inclusion of RCT-based meta-analyses, yielding 62% high-certainty evidence according to GRADE criteria.

Several limitations in our study need to be acknowledged. Firstly, raw patient-level data from all selected studies could not be obtained; hence, only modified study-level data were extracted and reanalysed in the present umbrella review. Although the assessable endpoints of interest in this umbrella review were objective in the included studies, some studies were still accompanied by a risk of bias. Secondly, the GRADE assessment revealed heterogeneity (*I*^*2*^ > 50%) and imprecision (continuous variables with total population size <400; binary categorical variables with events <300) across multiple meta-analyses. A key contributing factor is the small sample sizes, with 139 (51.29%) meta-analyses including fewer than five original studies, leading to inconsistent findings and imprecise estimates. This issue particularly affected outcomes such as gastrointestinal adverse events, drug discontinuation, ACS, MI, stroke, and neutrophil count. Although CV-related outcomes are conceptually comparable, variations in dosage, treatment duration, and study populations further contributed to heterogeneity and low-certainty evidence, as seen in outcomes like diarrhea, leukocyte counts, and restenosis after percutaneous coronary intervention. Nevertheless, low-certainty evidence does not rule out potential associations, especially as future studies may provide more robust data. Thirdly, we included only systematic reviews with meta-analyses that could be quantitatively analysed; systematic reviews that performed qualitative analysis and meta-analyses lacking study-specific data were excluded. However, we further listed findings from such studies to ensure that any unique cancer was considered. We found that colchicine could reduce the risk of CHD, stroke, and other diseases in the population with ASCVD, which was consistent with our findings and did not affect the final conclusion. Fourthly, the heterogeneity of endpoints and adverse events also introduced potential bias. Additionally, the definition of endpoint events was relatively broad; for instance, most associations did not distinguish between ischemic and hemorrhagic strokes, directly using stroke as the endpoints, which might overestimate or underestimate the effectiveness of colchicine as an intervention, leading to confusion in evaluating treatment outcomes and risks. Fifthly, emerging evidence suggests that COVID-19-induced chronic systemic inflammation may constitute a pathogenic driver of atherosclerotic cardiovascular disease (ASCVD),^108^ particularly in individuals lacking traditional risk factors. Our analysis identified methodological constraints in evaluating temporal associations due to insufficient post-pandemic comparator studies (n < 3), precluding robust assessment of COVID-19’s epidemiological contribution, however, this direction could become one of the entry points for our attention and future research.

Colchicine demonstrated therapeutic benefits in CHD and ACS, serving as an effective agent for secondary prevention of MACEs including AMI, UA, and cerebrovascular incidents. Its mechanism of action involves modulation of inflammatory pathways and enhancement of vascular repair processes, which collectively contribute to reduced hospitalisation requirements and improved revascularisation outcomes. While generally well-tolerated, treatment-emergent gastrointestinal disturbances and hepatic enzyme abnormalities represent the most frequently reported adverse effects, with dose-dependent correlations to therapy discontinuation and non-CV mortality risks. Clinical deployment requires careful consideration of patient-specific factors such as age-related pharmacokinetic variations, regional pharmacogenomic profiles, and physical changes of patients after surgery. Current evidence supports the adoption of conservative dosing regimens with extended treatment duration to optimise the risk–benefit ratio, though therapeutic protocols should remain adaptable to evolving clinical contexts.

## Contributors

R-HB, Q-PM, T-TG, and Q-JW contributed to the study design. R-HB, NZ, X-FJ, Z-HC, JL, and L-DK collection of data. R-HB and NZ accessed and verified the underlying data. R-HB, NZ, X-FJ, and Z-HC analysis of data. R-HB, NZ, X-FJ, Z-HC, Y-HC, J-NS, W-YX, J-XL, X-LB, H-CL, J-YZ, Q-PM, T-TG, and Q-JW wrote the first draft of the manuscript and edited the manuscript. All authors read and approved the final manuscript. R-HB, NZ, X-FJ, and Z-HC contributed equally to this work.

## Data sharing statement

Data sharing requests about the data supporting the conclusions of this article can be directed to the corresponding author.

## Declaration of interests

The authors declare no competing interests.
