# Supplementary material for: Balancing the benefits and risks of colchicine use among patients with atherosclerotic cardiovascular disease: an umbrella review of meta-analyses of randomised controlled trials
Source: eClinicalMedicine. 2025 Jun 5;84:103277. doi: 10.1016/j.eclinm.2025.103277 (PMC12179638; doi:10.1016/j.eclinm.2025.103277)
Supplement: Supplementary Fig. S1 and Tables S1–S12 [file mmc1.docx]

**Supplementary Materials**

| **Supplementary Table** **S1.** Preferred Reporting Items for Systematic Review and Meta-Analysis (PRISMA) checklist |
| --- |
| **Supplementary Table S2.** Search strategy |
| **Supplementary Table S3.** List of excluded records and reasons for exclusion during full-text review |
| **Supplementary Table S4.** The results of GRADE assessment of the evidence certainty on the association between colchicine intervention among patients with atherosclerotic cardiovascular disease |
| **Supplementary Table S5.** Sensitivity analysis results of excluded meta-analyses due to overlap in RCT studies |
| **Supplementary Table S6.** Summary of sensitivity analysis excluding studies with small sample size (< 25th percentile) |
| **Supplementary Table S7.** Summary of sensitivity analysis excluding studies with high risk of bias |
| **Supplementary Table S8.** The summary results of meta-analyses excluded due to lack of data for quantitative synthesis  **Supplementary Table S9.** Subgroup analyses according to the dose of colchicine use on assessed associations |
| **Supplementary Table S10.** Subgroup analyses according to the duration of colchicine use on assessed associations |
| **Supplementary Table S11.** Subgroup analysis according to the region of colchicine use on assessed associations |
| **Supplementary Table S12.** Subgroup analysis according to the age of colchicine use on assessed associations |
| **Supplementary Figure S1.** AMSTAR scores for each published meta-analysis |

## Supplementary Table S1. PRISMA checklist

| **Section and Topic** | **Item ^a^** | **Checklist item** | **Location where item is reported** |
| --- | --- | --- | --- |
| **TITLE** | | |  |
| Title | 1 | Identify the report as a systematic review. | 1-3 |
| **ABSTRACT** | | |  |
| Abstract | 2 | See the PRISMA 2020 for Abstracts checklist. | 40-86 |
| **INTRODUCTION** | | |  |
| Rationale | 3 | Describe the rationale for the review in the context of existing knowledge. | 137-153 |
| Objectives | 4 | Provide an explicit statement of the objective(s) or question(s) the review addresses. | 184-190 |
| **METHODS** | | |  |
| Eligibility criteria | 5 | Specify the inclusion and exclusion criteria for the review and how studies were grouped for the syntheses. | 209-237 |
| Information sources | 6 | Specify all databases, registers, websites, organizations, reference lists and other sources searched or consulted to identify studies. Specify the date when each source was last searched or consulted. | 200-207 |
| Search strategy | 7 | Present the full search strategies for all databases, registers and websites, including any filters and limits used. | 200-207 |
| Selection process | 8 | Specify the methods used to decide whether a study met the inclusion criteria of the review, including how many reviewers screened each record and each report retrieved, whether they worked independently, and if applicable, details of automation tools used in the process. | 200-207 |
| Data collection process | 9 | Specify the methods used to collect data from reports, including how many reviewers collected data from each report, whether they worked independently, any processes for obtaining or confirming data from study investigators, and if applicable, details of automation tools used in the process. | 239-249 |
| Data items | 10a | List and define all outcomes for which data were sought. Specify whether all results that were compatible with each outcome domain in each study were sought (e.g. for all measures, time points, analyses), and if not, the methods used to decide which results to collect. | 239-249 |
|  | 10b | List and define all other variables for which data were sought (e.g. participant and intervention characteristics, funding sources). Describe any assumptions made about any missing or unclear information. | 239-249 |
| Study risk of bias assessment | 11 | Specify the methods used to assess risk of bias in the included studies, including details of the tool(s) used, how many reviewers assessed each study and whether they worked independently, and if applicable, details of automation tools used in the process. | 262-272 |
| Effect measures | 12 | Specify for each outcome the effect measure(s) (e.g. risk ratio, mean difference) used in the synthesis or presentation of results. | 276-283 |
| Synthesis methods | 13a | Describe the processes used to decide which studies were eligible for each synthesis (e.g. tabulating the study intervention characteristics and comparing against the planned groups for each synthesis (item #5)). | 276-283 |
|  | 13b | Describe any methods required to prepare the data for presentation or synthesis, such as handling of missing summary statistics, or data conversions. | 276-283 |
|  | 13c | Describe any methods used to tabulate or visually display results of individual studies and syntheses. | 276-283 |
|  | 13d | Describe any methods used to synthesize results and provide a rationale for the choice(s). If meta-analysis was performed, describe the model(s), method(s) to identify the presence and extent of statistical heterogeneity, and software package(s) used. | 276-283 |
|  | 13e | Describe any methods used to explore possible causes of heterogeneity among study results (e.g. subgroup analysis, meta-regression). | 276-283 |
|  | 13f | Describe any sensitivity analyses conducted to assess robustness of the synthesized results. | 291-297 |
| Reporting bias assessment | 14 | Describe any methods used to assess risk of bias due to missing results in a synthesis (arising from reporting biases). | NA |
| Certainty assessment | 15 | Describe any methods used to assess certainty (or confidence) in the body of evidence for an outcome. | 262-272 |
| **RESULTS** | | |  |
| Study selection | 16a | Describe the results of the search and selection process, from the number of records identified in the search to the number of studies included in the review, ideally using a flow diagram. | 314-319 |
|  | 16b | Cite studies that might appear to meet the inclusion criteria, but which were excluded, and explain why they were excluded. | Supplementary Table S3 |
| Study characteristics | 17 | Cite each included study and present its characteristics. | Table 1 |
| Risk of bias in studies | 18 | Present assessments of risk of bias for each included study. | NA |
| Results of individual studies | 19 | For all outcomes, present, for each study: (a) summary statistics for each group (where appropriate) and (b) an effect estimate and its precision (e.g. confidence/credible interval), ideally using structured tables or plots. | 340-348 |
| Results of syntheses | 20a | For each synthesis, briefly summarize the characteristics and risk of bias among contributing studies. | 340-348 |
|  | 20b | Present results of all statistical syntheses conducted. If meta-analysis was done, present for each the summary estimate and its precision (e.g. confidence/credible interval) and measures of statistical heterogeneity. If comparing groups, describe the direction of the effect. | 351-436 |
|  | 20c | Present results of all investigations of possible causes of heterogeneity among study results. | 351-436 |
|  | 20d | Present results of all sensitivity analyses conducted to assess the robustness of the synthesized results. | 439-457 |
| Reporting biases | 21 | Present assessments of risk of bias due to missing results (arising from reporting biases) for each synthesis assessed. | NA |
| Certainty of evidence | 22 | Present assessments of certainty (or confidence) in the body of evidence for each outcome assessed. | 351-436 |
| **DISCUSSION** | | |  |
| Discussion | 23a | Provide a general interpretation of the results in the context of other evidence. | 560-643 |
|  | 23b | Discuss any limitations of the evidence included in the review. | 646-686 |
|  | 23c | Discuss any limitations of the review processes used. | 646-686 |
|  | 23d | Discuss implications of the results for practice, policy, and future research. | 687-691 |
| **OTHER INFORMATION** | | |  |
| Registration and protocol | 24a | Provide registration information for the review, including register name and registration number, or state that the review was not registered. | 196-197 |
|  | 24b | Indicate where the review protocol can be accessed, or state that a protocol was not prepared. | 196-197 |
|  | 24c | Describe and explain any amendments to information provided at registration or in the protocol. | 196-197 |
| Support | 25 | Describe sources of financial or non-financial support for the review, and the role of the funders or sponsors in the review. | 725-726 |
| Competing interests | 26 | Declare any competing interests of review authors. | 721-722 |
| Availability of data, code and other materials | 27 | Report which of the following are publicly available and where they can be found: template data collection forms; data extracted from included studies; data used for all analyses; analytic code; any other materials used in the review. | NA |
| *^a^ From: Page MJ, McKenzie JE, Bossuyt PM, Boutron I, Hoffmann TC, Mulrow CD, et al. The PRISMA 2020 statement: an updated guideline for reporting systematic reviews. BMJ 2021;372:n71. doi: 10.1136/bmj.n71* | | | |

## Supplementary Table S2. Search strategy

| **Literature search strategy in PubMed** |
| --- |
| #1 "meta-analysis"[Title/Abstract] OR "systematic review"[Title/Abstract] OR "systematic overview"[Title/Abstract] |
| #2 "colchicine"[All Fields] OR "colchicin"[All Fields] OR "colchicines"[All Fields] OR "colchicine’s"[All Fields] |
| #3 "cardiovascular diseases"[MeSH Terms] OR "cardiova"[All Fields] OR "mortality"[All Fields] OR "cardioma"[All Fields] OR "vascula"[All Fields] OR "coronary"[All Fields] OR "heart"[All Fields] OR "thrombosis"[All Fields] OR "hypertension"[All Fields] OR "peripheral"[All Fields] OR "fibrilla"[All Fields] OR "embolism"[All Fields] OR "stroke"[All Fields] OR "ischemia"[All Fields] |
| **Literature search strategy in Web of Science** |
| #1 "meta-analysis" OR "systematic review" OR "systematic overview" |
| #2 "colchicine" OR "colchicin" OR "colchicines" OR "colchicine’s" |
| #3 "cardiovascular diseases" OR "cardiova" OR "mortality" OR "cardioma" OR "vascula" OR "coronary" OR "heart" OR "thrombosis" OR "hypertension" OR "peripheral" OR "fibrilla" OR "embolism" OR "stroke" OR "ischemia" |
| **Literature search strategy in Embase** |
| #1 'meta-analysis':ti,ab OR 'systematic review':ti,ab OR 'systematic overview':ti,ab |
| #2 'colchicine'/exp OR colchicine:ti,ab |
| #3 'cardiovascular disease'/exp OR cardiovascular OR 'mortality'/exp OR mortality OR 'cardioma' OR 'vascula' OR coronary OR 'heart'/exp OR heart OR 'thrombosis'/exp OR thrombosis OR 'hypertension'/exp OR hypertension OR 'peripheral' OR 'fibrilla' OR 'embolism'/exp OR embolism OR 'stroke'/exp OR stroke OR 'ischemia' |
| **Literature search strategy in Cochrane Library** |
| #1 "meta-analysis" OR "systematic review" OR "systematic overview" |
| #2 "colchicine" OR "colchicin" OR "colchicines" OR "colchicine's" |
| #3 "cardiovascular diseases" OR "cardiova" OR "mortality" OR "cardioma" OR "vascula" OR "coronary" OR "heart" OR "thrombosis" OR "hypertension" OR "peripheral" OR "fibrilla" OR "embolism" OR "stroke" OR "ischemia" |

| Supplementary Table S3. List of excluded records and reasons for exclusion during full-text review |
| --- |
| **Not a meta-analysis or systematic review with quantitative synthesis (n=93)** |
| 1. Alam M, Jhamnani S. ROLE OF COLCHICINE IN ACUTE AND CHRONIC CORONARY SYNDROMES: AN UPDATED METAANALYTIC COMPARISON. Journal of the American College of Cardiology 2024, 83(13):2247. |
| 2. Al-Sadawi M, Tokavanich N, Devgun JK et al. PO-03-069 EFFECT OF COLCHICINE ON THE OUTCOMES OF CATHETER ABLATION FOR ATRIAL FIBRILLATION. Heart Rhythm 2024, 21(5):S393. |
| 3. Asllanaj B, Benge E, Dhaliwal A et al. STILL'S THE ONE: A RARE CASE OF ADULT ONSET STILL'S DISEASE. Chest 2023, 164(4):A1527. |
| 4. Banjan T, Ghosh S, Kundu M et al. A systematic review on the efficacy of colchicine in hemorrhagic stroke patients. Journal of the Neurological Sciences 2023, 455. |
| 5. Bates ER. In patients with CAD, adding colchicine to evidence-based therapies reduces MACE without increasing adverse outcomes. Annals of internal medicine 2021, 174(3):Jc30. |
| 6. Bautista J, Bailon D, Te-Rosano A et al. 460 Colchicine in Patients With Acute Myocardial Infarction: A Meta-Analysis of randomised controlled trials. Heart Lung and Circulation 2020, 29:S243. |
| 7. Bayes-Genis A, Adler Y, Bayes de Luna A et al. Colchicine in Pericarditis. European heart journal 2017, 38^19^:1706-1709. |
| 8. Biondi-Zoccai G, Lotrionte M, Imazio M et al. An International Collaborative Meta-Analysis on Pharmacologic Therapy of Acute Pericarditis. Circulation 2010, 122^21^. |
| 9. Blake N, Alonso A, Rai H et al. A meta-analysis of randomised controlled trials investigating the impact of colchicine on major adverse cardiovascular events in acute coronary syndrome. European heart journal 2021, 42:1221-1221. |
| 10. Boczar K, Alqarawi W, Derzi S et al. COLCHICINE THERAPY FOR REDUCTION OF CARDIOVASCULAR MORTALITY POST-ACUTE CORONARY SYNDROME: A SYSTEMATIC REVIEW AND META-ANALYSIS. Canadian Journal of Cardiology 2020, 36(10):S8-S9. |
| 11. Boczar KE, Shin S, Pearson A et al. Anti-Inflammatory Therapies to Prevent Cardiovascular Events: A Systematic Review and Network Meta-Analysis of randomised controlled trials. Circulation 2023, 148. |
| 12. Boczar KE, Shin S, Pearson A et al. Anti-Inflammatory Therapies to Prevent Cardiovascular Events: A Systematic Review and Network MetaAnalysis of randomised controlled trials. Circulation 2023, 148. |
| 13. Briasoulis A, Afonso L. Prevention of pericarditis with colchicine: an updated meta-analysis. Journal of cardiovascular medicine (Hagerstown, Md) 2015, 16(2):144-147. |
| 14. Briasoulis A, Mostafa A, Siddiqui F et al. PREVENTION OF RECURRENT PERICARDITIS WITH COLCHICINE: AN UPDATED META-ANALYSIS. Journal of the American College of Cardiology 2015, 65(10):A1418-A1418. |
| 15. Buda K, Megaly M, Padniewski J et al. SAFETY AND EFFICACY OF COLCHICINE FOR SECONDARY PREVENTION AFTER ACUTE MYOCARDIAL INFARCTION: A SYSTEMATIC REVIEW AND META-ANALYSIS. Journal of the American College of Cardiology 2021, 77^18^:1522-1522. |
| 16. Chow B, Souders P, Tyacke M et al. DELAYED TRAUMATIC PERICARDIAL TAMPONADE FOLLOWING BLUNT CHEST AND MULTISYSTEM TRAUMA. Chest 2019, 156(4):A1500-A1501. |
| 17. Chugh R, Proctor D, Little A et al. LEUKOCYTOCLASTIC VASCULITIS AFTER USTEKINUMAB INDUCTION IN CROHN'S DISEASE: A CASE SERIES AND SYSTEMATIC REVIEW. Gastroenterology 2020, 158(3):S3-S3. |
| 18. Condello F, Sturla M, Liccardo G et al. Colchicine in patients with coronary artery disease: a meta-analysis of randomized trials. European Heart Journal Supplements 2021, 23(G). |
| 19. Costain N, Choi S, Vaillancourt C. Colchicine in acute and recurrent pericarditis: A meta-analysis. Canadian Journal of Emergency Medicine 2016, 18:S64. |
| 20. Diaz-Arocutipa C, Benites-Meza J, Chambergo-Michilot D et al. Efficacy and safety of colchicine after myocardial infarction: a systematic review and meta- analysis. European heart journal 2021, 42:1418-1418. |
| 21. Divinagracia RZ, Vistal G, Tiongco RH. Effect of colchicine on reducing C-reactive protein levels among patients with acute coronary syndrome: a meta-analysis. European heart journal 2021, 42:1297-1297. |
| 22. Duman NC, Karabacak M, Oglu MGI et al. Colchicine use during pregnancy: Case reports. Annals of the Rheumatic Diseases 2019, 78:2082-2083. |
| 23. Dumitrescu C, Mehta K, Singh V. WHEN THE HEART HAS THE FLU: INFLUENZA B RESULTING IN CARDIAC TAMPONADE. Chest 2019, 156(4):A803. |
| 24. EAS 2020 Congress. Atherosclerosis 2020, 315:e1-e282. |
| 25. El Iskandarani M, Shatla I, Khalid M et al. COLCHICINE IN STABLE CORONARY ARTERY DISEASE, A SYSTEMATIC REVIEW AND METANALYSIS OF RANDOMIZED CLINICAL TRIALS. Journal of the American College of Cardiology 2021, 77^18^:179-179. |
| 26. Escalera E, Saver J. Magnitude of Effect of Low Dose Colchicine, a Newly FDA Approved Treatment for Stroke Prevention. Neurology 2024, 102^17^. |
| 27. Ewald H, Gloy VL, Glinz D et al. Colchicine for prevention of cardiovascular events: A systematic review and meta-analysis. Praxis 2015, 104:40-41. |
| 28. Fadhil A, Al Wssawi A, Gumera A et al. COLCHICINE REDUCES MAJOR ADVERSE CARDIOVASCULAR EVENTS IN PATIENTS UNDERGOING PERCUTANEOUS CORONARY INTERVENTION: A META-ANALYSIS OF randomised controlled trials. Journal of the American College of Cardiology 2024, 83(13):919-919. |
| 29. Fomaneg CK, Samonte PEE, Sasondoncillo-Nadal MM et al. Colchicine use for the primary prevention of postpericardiotomy syndrome (CUPP): A meta-analysis. Cardiology (Switzerland) 2014, 128:475. |
| 30. Fong HK, Tan JL, Eniezat M et al. Outcomes of anti-inflammatory agents in coronary artery disease. Catheterization and Cardiovascular Interventions 2020, 95:S204-S205. |
| 31. Furqan M, Abdullah R, Verma B et al. EFFECT OF ANAKINRA ON COLCHICINE RESISTANT AND STEROID DEPENDENT RECURRENT PERICARDITIS: A SYSTEMIC REVIEW AND META-ANALYSIS. Journal of the American College of Cardiology 2019, 73(9):1017-1017. |
| 32. Goh C, Tan YK, Leow A et al. The use of colchicine as an antiinflammatory agent for stroke prevention in patients with coronary artery disease: A systematic review and meta-analysis. European Stroke Journal 2021, 6(1 SUPPL):287-288. |
| 33. Hussain M, Garg R, Matsumura M. A Rare Case Of Salmonella Perimyocarditis In A Young Healthy Male. Journal of Cardiac Failure 2024, 30(1):146. |
| 34. Imazio M. Colchicine and new management stategies for acute and recurrent pericarditis. Cardiology (Switzerland) 2014, 128:435. |
| 35. Imazio M, Brucato A, Belli R et al. Systematic review and meta-analysis on the efficacy and safety of colchicine for pericarditis prevention. European heart journal 2012, 33:440-440. |
| 36. Imazio M, Brucato A, Belli R et al. Colchicine for the prevention of pericarditis: systematic review and meta-analysis. European heart journal 2014, 35:921-921. |
| 37. Imazio M, Brucato A, Cemin R et al. Prevention of the post-pericardiotomy syndrome. A meta-analysis of published randomized trials. Circulation 2012, 125^19^:E770-E771. |
| 38. Imazio M, Brucato A, Cemin R et al. Prevention of the post-pericardiotomy syndrome. A metaanalysis of published randomized trials. Giornale Italiano di Cardiologia 2011, 12(12):e108. |
| 39. Imazio M, Gaita F, LeWinter M. Evaluation and Treatment of Pericarditis: A Systematic Review (vol 314, pg 1498, 2015). Jama-Journal of the American Medical Association 2015, 314^18^:1978-1978. |
| 40. Indraratna P, Virk S, Gurram D. Management of pericarditis in pregnancy: A systematic review and meta-analysis. Heart Lung and Circulation 2017, 26:S297. |
| 41. Ishaq S, Singh S, Upreti P et al. TCT-178 Effect of Colchicine on the Cardiovascular Outcomes in Patients With Acute Coronary Syndromes. In., vol. 84; 2024: B6. |
| 42. Jansen Dirken-Heukensfeldt KJM, Teunissen TAM, Van De Lisdonk EH et al. Clinical features of women with gout arthritis. A systematic review. Clinical rheumatology 2010, 29(6):575-582. |
| 43. Jimenez CS, Sanchez JS, Hayat F et al. TCT-361 Clinical Outcomes in Patients Undergoing Percutaneous Coronary Intervention Treated With Colchicine. Journal of the American College of Cardiology 2021, 78^19^:B148-B149. |
| 44. Kalra K, Jaiswal V, Jaiswal A et al. Efficacy of Colchicine for Prevention of Stroke and Major Adverse Cardiovascular Events in Patients With Coronary Artery Disease: A Meta-Analysis of 15 randomised controlled trials. Stroke 2024, 55. |
| 45. Kasap Cuceoglu M, Sener S, Batu ED et al. Systematic review of childhood-onset polyarteritis nodosa and DADA2. Seminars in arthritis and rheumatism 2021, 51(3):559-564. |
| 46. Kassab K, Chuy KL, Vij A. USE OF COLCHICINE FOR SECONDARY PREVENTION OF CARDIOVASCULAR EVENTS: SYSTEMATIC REVIEW AND META-ANALYSIS. Journal of the American College of Cardiology 2021, 77^18^:24-24. |
| 47. Khan MU, Lone AN, Khan SU et al. Meta-analysis of efficacy and safety of colchicine therapy in patients with acute coronary syndrome. Circulation 2021, 144(SUPPL 1). |
| 48. Khine S, Edupuganti S, Bachuwa G. A Case of Severe Cardiomyopathy Due to Covid-Induced Myocarditis, Completely Resolved after Colchicine and Immunoglobulin Therapy. European journal of case reports in internal medicine 2023, 10(9):003877. |
| 49. Kreidieh O, Kabach M, El Dassouki S et al. COLCHICINE VERSUS PLACEBO FOR PREVENTION OF POST-PROCEDURAL ATRIAL FIBRILLATION: A SYSTEMATIC REVIEW AND META-ANALYSIS. Cardiology 2016, 134:227-227. |
| 50. Kundu A, Sardar P, Ghosh S et al. EFFICACY AND SAFETY OF COLCHICINE FOR PREVENTION OF PERICARDITIS AND CARDIAC TAMPONADE: AN UPDATED META-ANALYSIS OF randomised controlled trials. Journal of the American College of Cardiology 2016, 67(13):1901-1901. |
| 51. Langnas E, Mustafa S, Vira A et al. RAPIDLY PROGRESSING PURULENT PERICARDITIS CAUSED BY NEISSERIA MENINGITIDES INFECTION. Chest 2020, 158(4):A475. |
| 52. Lee JZ, Singh N, Huang J et al. COLCHICINE FOR PREVENTION OF POST-PROCEDURAL ATRIAL FIBRILLATION: A META-ANALYSIS. Journal of the American College of Cardiology 2015, 65(10):A376-A376. |
| 53. Li Z, Zeng Z, Yuan S et al. Perioperative Interventions for Prevention of Postoperative Atrial Fibrillation After Cardiac Surgery: A Systematic Review and Network Meta-Analysis. Circulation 2021, 144. |
| 54. Liang H, Huang D. Comment on "Efficacy and safety of colchicine in patients with coronary artery disease: A systematic review and meta-analysis of randomised controlled trials". British journal of clinical pharmacology 2022, 88(6):3000-3001. |
| 55. Lin C, Li Z, Cai X et al. Anti-inflammatory Therapies in Patients with Established Cardiovascular Disease or High Cardiovascular Risks. Diabetes 2023, 72. |
| 56. Lutschinger LL, Rigopoulos AG, Schlattmann P et al. Correction to: Meta-analysis for the value of colchicine for the therapy of pericarditis and of postpericardiotomy syndrome (BMC Cardiovascular Disorders (2019) 19 (207) DOI: 10.1186/s12872-019-1190-4). BMC cardiovascular disorders 2019, 19(1). |
| 57. Manara M, Bortoluzzi A, Favero M et al. Italian Society of Rheumatology recommendations for the management of gout. Reumatismo 2013, 65(1):4-21. |
| 58. Mendoza R, Bailon D, Bernardo J et al. Colchicine in patients with acute myocardial infarction: an updated meta-analysis of randomised controlled trials. European heart journal 2021, 42:1273-1273. |
| 59. Merriman TR, Phipps-Green A, Cadzow M et al. Confirmation of association of urate transporter SLC17A1 (NPT1) with gout at a genome-wide level of significance. Arthritis and Rheumatism 2011, 63(10). |
| 60. Meune C, Spaulding C, Mahé I et al. Risks versus benefits of NSAIDs including aspirin in myocarditis -: A review of the evidence from animal studies. Drug safety 2003, 26(13):975-981. |
| 61. Michel Noutsias M, Schlattmann P, Aftanski P et al. Meta-analysis on the clinical effects of colchicine in recurrent pericarditis. European Journal of Heart Failure 2016, 18:470. |
| 62. Mohee K, Zhang J, Cleland JG et al. USE OF COLCHICINE IN CARDIOVASCULAR DISEASES - A SYSTEMATIC REVIEW. Heart (British Cardiac Society) 2015, 101:A91-A92. |
| 63. Nguyen C, Gitzel L, Jacubowsky A et al. SCLEROSING PERICARDITIS AND DECOMPENSATED HEART FAILURE AFTER COVID-19 INFECTION. Chest 2023, 164(4):A611-A612. |
| 64. Nogic J, Mehta O, Tong D et al. Correction to: Colchicine in the Management of Acute Coronary Syndrome: A Meta-analysis (Cardiology and Therapy, (2023), 12, 1, (171-181), 10.1007/s40119-022-00298-y). Cardiology and therapy 2023, 12(3):539-541. |
| 65. Papageorgiou N, Briasoulis A, Lazaros G et al. COLCHICINE FOR PREVENTION AND TREATMENT OF CARDIAC DISEASES: AN UPDATED META-ANALYSIS. Journal of the American College of Cardiology 2016, 67(13):1965-1965. |
| 66. Patel J, Lau C, O'Hea JA et al. EPSTEIN-BARR VIRUS AS A RARE CAUSE OF ACUTE PERICARDITIS AND CARDIAC TAMPONADE. In., vol. 166; 2024: A1326. |
| 67. Perkovic A, Webster E, Ridker P et al. The Effects of Anti-Inflammatory Agents on Cardiovascular Outcomes: A Systematic Review and Meta-Analysis of Randomised Controlled Clinical Trials. In., vol. 33; 2024: S539. |
| 68. Ponir C. DIAGNOSIS AND TREATMENT OF A RARE POSTERIORLY LOCULATED PERICARDIAL EFFUSION SECONDARY TO FOCAL PERICARDITIS. Chest 2023, 164(4):A520-A521. |
| 69. Pradhan AD. Time to commence or time out for colchicine in secondary prevention of cardiovascular disease? European heart journal 2021, 42^17^:2776-2779. |
| 70. Putra BP, Putra FN. Expanding the potential benefits of colchicine for preventing postpericardiotomy syndrome and atrial fibrillation complications after cardiac surgery: Meta-analysis of randomised controlled trials. European journal of preventive cardiology 2021, 28(SUPPL 1):i113. |
| 71. Richette P, Doherty M, Pascual E et al. Updated EULAR evidence-based recommendations for the management of gout. Annals of the Rheumatic Diseases 2014, 73. |
| 72. Rivera FB, Whoy Cha S, Aparece JP et al. Efficacy and Safety of Colchicine for the Prevention of Postoperative Atrial Fibrillation Among Patients Undergoing Major Cardiothoracic Surgery: A Meta-analysis and Meta-regression of randomised controlled trials. Journal of cardiovascular pharmacology 2024, 83(3):265-270. |
| 73. Rout A, Garg A, Tantry U et al. Colchicine in coronary artery disease: A meta-analysis of randomised controlled trials. Catheterization and Cardiovascular Interventions 2020, 95:S208-S209. |
| 74. Sabina M, Shah S, Trube J et al. TCT-308 Colchicine for the Prevention of Recurrent Non-Cardioembolic Stroke: A Meta-Analysis of randomised controlled trials. In., vol. 84; 2024: B72. |
| 75. Salih M, Smer A, Elmasry Y et al. COLCHICINE FOR PREVENTION OF POSTOPERATIVE ATRIAL FIBRILLATION: META-ANALYSIS OF randomised controlled trials. Journal of the American College of Cardiology 2015, 65(10):A268-A268. |
| 76. Salih MS, Mlatoum HA, Leung E et al. Colchicine for prevention of pericarditis recurrence: Metaanalysis of randomised controlled trials. Cardiology (Switzerland) 2015, 131:118. |
| 77. Sayad R, Siddiq A, Hashim A et al. Can the current monkeypox affect the heart? A systematic review of case series and case report. BMC cardiovascular disorders 2023, 23(1):328. |
| 78. Schattner A. Colchicine – new horizons for an ancient drug. Review based on the highest hierarchy of evidence. European journal of internal medicine 2022, 96:34-41. |
| 79. Stewart S, Yang K, Atkins K et al. Adverse Events During Colchicine Use: A Systematic Review and Meta-Analysis of Randomized Controlled Trial Events. Arthritis & Rheumatology 2019, 71. |
| 80. Sunjaya AF, Sunjaya AP. Is there value in prescribing colchicine in coronary artery disease for secondary prevention: a meta-analysis of over 5,000 patients. European heart journal 2020, 41:1498-1498. |
| 81. Swamy PM, Mamas MA. Giving Drugs a Second Chance. Cardiovascular revascularization medicine : including molecular interventions 2021, 28:98-99. |
| 82. Tsivgoulis G, Katsanos AH, Palaiodimou L et al. COLCHICINE FOR STROKE PREVENTION IN PATIENTS WITH HISTORY OF CORONARY ARTERY DISEASE: A SYSTEMATIC REVIEW AND META-ANALYSIS. International Journal of Stroke 2020, 15(1_SUPPL):150-150. |
| 83. Tucker B, Houston L, Tucker W et al. Safety and Tolerability of Regular Oral Colchicine—A Systematic Review and Meta-Analysis of Randomised Placebo-Controlled Trials. In., vol. 33; 2024: S371. |
| 84. Upadhaya S, Madala S, Tiwari K. COLCHICINE FOR SECONDARY PREVENTION OF CARDIOVASCULAR ADVERSE EVENTS IN PATIENTS WITH CORONARY ARTERY DISEASE: AN UPDATED META-ANALYSIS. Journal of the American College of Cardiology 2021, 77^18^:17-17. |
| 85. Varotto L, Bonanno C, Caprioglio F. Network meta-analysis evaluating the utility of non-corticosteroid anti-inflammatory therapy in secondary prevention of coronaryartery disease. European Heart Journal, Supplement 2021, 23(SUPPL C):C114-C115. |
| 86. Vela S, Agrawal D, Khurana S et al. Colchicine for primary biliary cirrhosis: A meta-analysis of prospective controlled trials. Gastroenterology 2004, 126(4):A671-A672. |
| 87. Vikash J, Akash J, Nitya B et al. Symptomatology, prognosis and clinical findings of myocarditis as an adverse event of COVID-19 mRNA vaccine: a systematic review. European heart journal 2022, 43. |
| 88. Westphal JG, Schlattmann P, Lutschinger LL et al. Meta-analysis for the value of colchicine for the therapy and prevention of recurrence of pericarditis, and for rehospitalization for pericarditis and postpericardiotomy syndrome. European heart journal 2017, 38:1347-1347. |
| 89. Westphal JG, Schlattmann P, Lutschinger LL et al. Meta-analysis for the value of colchicine for the therapy and prevention of pericarditis and postpericardiotomy syndrome. European Journal of Heart Failure 2017, 19:92-93. |
| 90. Xie S, Galimberti F, Olmastroni E et al. Network meta-analysis comparing the effect of lipid-lowering treatments and colchicine on C-reactive protein concentration. In., vol. 395; 2024. |
| 91. Zaman N, Grewal S, Borgatta L et al. Colchicine for Secondary Prevention of Adverse Outcomes From Cardiovascular Disease: A Systematic Review and Meta-analysis of randomised controlled trials. Circulation 2020, 142. |
| 92. Zhao H, Chen Y, Mao M et al. Correction: A meta‑analysis of colchicine in prevention of atrial fibrillation following cardiothoracic surgery or cardiac intervention. Journal of cardiothoracic surgery 2022, 17(1):285. |
| 93. Zhao H, Chen Y, Mao M et al. Erratum: Correction: A meta‑analysis of colchicine in prevention of atrial fibrillation following cardiothoracic surgery or cardiac intervention (Journal of cardiothoracic surgery (2022) 17 1 (224)). Journal of cardiothoracic surgery 2022, 17(1):285. |
| **Studies that focused on observational studies, laboratory studies, or animal studies (n=26)** |
| 1. Alabed S, Pérez-Gaxiola G, Burls A. Colchicine for children with pericarditis: systematic review of clinical studies. Archives of disease in childhood 2016, 101(10):953-956. |
| 2. Alsabri M, Elsayed SM, Elsnhory AB et al. Efficacy and Safety of Colchicine in Pediatric Pericarditis: A Systematic Review and Future Directions. Pediatric cardiology 2024. |
| 3. Asenjo-Lobos C, Gonzalez L, Bulnes JF et al. Cardiovascular events risk in patients with systemic autoimmune diseases: a prognostic systematic review and meta-analysis. Clinical Research in Cardiology 2023. |
| 4. Borra V, Mahadevan A, Gautam Senapati S et al. The efficacy of colchicine in preventing atrial fibrillation recurrence and pericarditis post-catheter ablation for atrial fibrillation – A systematic review and meta-analysis of prospective studies. IJC Heart and Vasculature 2024, 53. |
| 5. Gao H, Liu S, Qin S et al. Injectable hydrogel-based combination therapy for myocardial infarction: a systematic review and Meta-analysis of preclinical trials. BMC cardiovascular disorders 2024, 24(1). |
| 6. Geisler BP, Zahabi L, Lang AE et al. Repurposing existing medications for coronavirus disease 2019: protocol for a rapid and living systematic review. Systematic Reviews 2021, 10(1). |
| 7. Harlianto NI, Harlianto ZN. Patient characteristics, surgical treatment, and outcomes in spinal gout: a systematic review of 315 cases. European Spine Journal 2023, 32(11):3697-3703. |
| 8. Imazio M, Lazaros G, Picardi E et al. Intravenous human immunoglobulins for refractory recurrent pericarditis: a systematic review of all published cases. Journal of cardiovascular medicine (Hagerstown, Md) 2016, 17(4):263-269. |
| 9. Kolkhir P, Grakhova M, Bonnekoh H et al. Treatment of urticarial vasculitis: A systematic review. The Journal of allergy and clinical immunology 2019, 143(2):458-466. |
| 10. Kommu S, Arepally S. The Effect of Colchicine on Atrial Fibrillation: A Systematic Review and Meta-Analysis. Cureus 2023, 15(2):e35120. |
| 11. Kyriakoulis KG, Kollias A, Diakos GE et al. Chlamydia pneumoniae-associated pleuropericarditis: a case report and systematic review of the literature. BMC pulmonary medicine 2021, 21(1):380. |
| 12. Li W, Sun J, Yu Y et al. Clinical Features of Post Cardiac Injury Syndrome Following Catheter Ablation of Arrhythmias: Systematic Review and Additional Cases. Heart Lung and Circulation 2019, 28(11):1689-1696. |
| 13. Mahalwar G, Kumar A, Agrawal A et al. Pericardial Involvement in Sarcoidosis. The American journal of cardiology 2022, 170:100-104. |
| 14. Merashli M, Bucci T, Pastori D et al. Intima media thickness of carotid arteries in familial Mediterranean fever: a systematic review and meta-analysis. Clinical rheumatology 2022, 41(12):3769-3776. |
| 15. Roddy E, Bajpai R, Forrester H et al. SAFETY OF COLCHICINE OR NSAID PROPHYLAXIS WHEN INITIATING ALLOPURINOL FOR GOUT: PROPENSITY SCORE-MATCHED COHORT STUDIES. Rheumatology (United Kingdom) 2022, 61(SUPPL 1):i90. |
| 16. Santolaya-Perrín R, Jiménez-Díaz G, Galán-Ramos N et al. A randomised controlled trial on the efficacy of a multidisciplinary health care team on morbidity and mortality of elderly patients attending the Emergency Department. Study design and preliminary results. Farmacia Hospitalaria 2016, 40(5):371-384. |
| 17. Schwier NC, Cornelio CK, Boylan PM. A systematic review of the drug-drug interaction between statins and colchicine: Patient characteristics, etiologies, and clinical management strategies. Pharmacotherapy 2022, 42(4):320-333. |
| 18. Schwier NC, Cornelio CK, Boylan PM. A systematic review of the drug–drug interaction between statins and colchicine: Patient characteristics, etiologies, and clinical management strategies. Pharmacotherapy 2022, 42(4):320-333. |
| 19. Sethuramalingam S, Maiti R, Hota D et al. Effect of Colchicine in Reducing Inflammatory Biomarkers and Cardiovascular Risk in Coronary Artery Disease: A Meta-analysis of Clinical Trials. American journal of therapeutics 2023, 30(3):e197-e208. |
| 20. Siak J, Flint N, Shmueli HG et al. The Use of Colchicine in Cardiovascular Diseases: A Systematic Review. The American journal of medicine 2021, 134(6):735-744.e731. |
| 21. Siddiqui MU, Junarta J, Sathyanarayanan S et al. Risk of coronary artery disease in patients with gout on treatment with Colchicine: A systematic review and meta-analysis. International journal of cardiology Heart & vasculature 2023, 45:101191. |
| 22. Soria Jiménez CE, Sánchez JS, Levine MB et al. Clinical outcomes of patients undergoing percutaneous coronary intervention treated with colchicine. REC: Interventional Cardiology 2023, 5(2):110-117. |
| 23. Ullah W, Gowda SN, Fischman D. Safety and Efficacy of Colchicine in Patients With Coronary Artery Disease: A Systematic Review and Meta-Analysis. Cardiovascular revascularization medicine : including molecular interventions 2021, 23:1-6. |
| 24. Wan H, Zeng L, Xiao R et al. Colchicine linked with risk reduction for myocardial infarction in gout patients: systematic review and meta-analysis. Zeitschrift fur Rheumatologie 2022, 81(6):501-506. |
| 25. Xiong H, Huang X, Rao L et al. Efficacy and safety of colchicine in the treatment of acute myocardial infarction: A protocol for systematic review and meta-analysis. Medicine 2021, 100^14^:e25429. |
| 26. Yasuhara J, Masuda K, Aikawa T et al. Myopericarditis after COVID-19 mRNA Vaccination among Adolescents and Young Adults: A Systematic Review and Meta-analysis. JAMA Pediatrics 2023, 177(1):42-52. |
| **Systematic reviews without quantitative analysis (n=6)** |
| 1. Boczar KE, Shin S, Bezzina KA et al. Examining anti-inflammatory therapies in the prevention of cardiovascular events: protocol for a systematic review and network meta-analysis of randomised controlled trials. BMJ open 2022, 12(6):e062702. |
| 2. Fiolet ATL, Nidorf SM, Mosterd A et al. Colchicine in Stable Coronary Artery Disease. Clinical therapeutics 2019, 41(1):30-40. |
| 3. Kundu M, Ghosh S, Shree A et al. A systematic review on the use of Colchicine in Hemorrhagic Stroke. World neurosurgery: X 2024, 23:100314. |
| 4. Madanchi M, Young M, Tersalvi G et al. The impact of colchicine on patients with acute and chronic coronary artery disease. European journal of internal medicine 2024, 125:1-9. |
| 5. McKnight AH, Katzenberger DR, Britnell SR. Colchicine in Acute Coronary Syndrome: A Systematic Review. The Annals of pharmacotherapy 2021, 55(2):187-197. |
| 6. Schattner A. Colchicine - new horizons for an ancient drug. Review based on the highest hierarchy of evidence. European journal of internal medicine 2022, 96:34-41. |
| **Studies that did not report comprehensive data (n=5)** |
| 1. Arboleda V, Hackworth A, Bonnice S et al. The role of aspirin, statins, colchicine, and IL-1 inhibitors in prevention of cardiovascular events: a systematic integrative review. Journal of osteopathic medicine 2024, 124(3):97-106. |
| 2. Somani N, Breur H. The Efficacy of Corticosteroids, NSAIDs, and Colchicine in the Treatment of Pediatric Postoperative Pericardial Effusion. Pediatric cardiology 2022, 43(2):279-289. |
| 3. Ters P, Badgett RG. A living meta-analysis of colchicine for pericarditis. The Annals of pharmacotherapy 2014, 48(10):1398-1399. |
| 4. Tucker B, Goonetilleke N, Patel S et al. Colchicine in atherosclerotic cardiovascular disease. Heart (British Cardiac Society) 2024, 110(9):618-625. |
| 5. Webb CA, Barry AR. Colchicine for Secondary Cardiovascular Prevention: A Systematic Review. Pharmacotherapy 2020, 40(6):575-583. |
| **Meta-analyses including fewer than three randomised controlled trials (n=7)** |
| 1. Bulhões E, Florêncio de Mesquita C, Madeira de Sá Pacheco I et al. Effects of colchicine on the prevention of AF recurrence after atrial ablation: a systematic review and meta-analysis. Journal of interventional cardiac electrophysiology : an international journal of arrhythmias and pacing 2024, 67(8):1951-1958. |
| 2. Imazio M, Brucato A, Markel G et al. Meta-analysis of randomized trials focusing on prevention of the postpericardiotomy syndrome. The American journal of cardiology 2011, 108(4):575-579. |
| 3. Jaiswal S, Liu X-b, Wei Q-c et al. Effect of corticosteroids on atrial fibrillation after catheter ablation: a meta-analysis. Journal of Zhejiang University-Science B 2018, 19(1):57-64. |
| 4. Nogic J, Mehta O, Tong D et al. Colchicine in the Management of Acute Coronary Syndrome: A Meta-analysis. Cardiology and therapy 2023, 12(1):171-181. |
| 5. Ou Z, Wang F, Chen Y et al. Comparative Efficacy of Colchicine and Intensive Low-density Lipoprotein Cholesterol Lowering in Patients with Atherosclerotic Diseases receiving Statins: A Network Meta-analysis of randomised controlled trials. Cardiovascular drugs and therapy 2024. |
| 6. Wiysonge CS, Ntsekhe M, Thabane L et al. Interventions for treating tuberculous pericarditis. The Cochrane database of systematic reviews 2017, 9(9):Cd000526. |
| 7. Wudexi I, Shokri E, Abo-Aly M et al. Comparative Effectiveness of Anti-Inflammatory Drug Treatments in Coronary Heart Disease Patients: A Systematic Review and Network Meta-Analysis. Mediators of inflammation 2021, 2021:5160728. |
| **Not in English (n=2)** |
| 1. Caldeira D, Vaz-Carneiro A, Costa J. Cochrane Corner: Colchicine in acute and recurrent pericarditis. Revista portuguesa de cardiologia : orgao oficial da Sociedade Portuguesa de Cardiologia = Portuguese journal of cardiology : an official journal of the Portuguese Society of Cardiology 2015, 34(11):697-699. |
| 2. Masson W, Lobo M, Lavalle-Cobo A et al. ¿La colchicina puede prevenir el infarto agudo de miocardio? Revisión sistemática y metaanálisis. Revista argentina de cardiología 2021, 89(1):42-49. |
| **Patients without Atherosclerotic cardiovascular disease (n=26)** |
| 1. Hemkens LG, Ewald H, Gloy VL et al. Cardiovascular effects and safety of long-term colchicine treatment: Cochrane review and meta-analysis. Heart (British Cardiac Society) 2016, 102(8):590-596. |
| 2. Salih M, Smer A, Charnigo R et al. Colchicine for prevention of post-cardiac procedure atrial fibrillation: Meta-analysis of randomised controlled trials. International journal of cardiology 2017, 243:258-262. |
| 3. Lee JZ, Singh N, Howe CL et al. Colchicine for Prevention of Post-Operative Atrial Fibrillation: A Meta-Analysis. JACC Clinical electrophysiology 2016, 2(1):78-85. |
| 4. Lennerz C, Barman M, Tantawy M et al. Colchicine for primary prevention of atrial fibrillation after open-heart surgery: Systematic review and meta-analysis. International journal of cardiology 2017, 249:127-137. |
| 5. Imazio M, Brucato A, Belli R et al. Colchicine for the prevention of pericarditis: what we know and what we do not know in 2014 - systematic review and meta-analysis. Journal of cardiovascular medicine (Hagerstown, Md) 2014, 15(12):840-846. |
| 6. Li YL, Qiao SB, Wang JY et al. Colchicine in addition to conventional therapy for pericarditis recurrence : An update meta-analysis. Herz 2016, 41(7):630-638. |
| 7. Trivedi C, Sadadia M. Colchicine in prevention of atrial fibrillation following cardiac surgery: systematic review and meta-analysis. Indian journal of pharmacology 2014, 46(6):590-595. |
| 8. Jain H, Odat RM, Dey D et al. Colchicine Prevents Post-Ablation Atrial Fibrillation Recurrence: A Systematic Review and Meta-Analysis. Cardiology in review 2024. |
| 9. Wang MX, Deng XL, Mu BY et al. Effect of colchicine in prevention of pericardial effusion and atrial fibrillation: a meta-analysis. Internal and emergency medicine 2016, 11(6):867-876. |
| 10. Agarwal SK, Vallurupalli S, Uretsky BF et al. Effectiveness of colchicine for the prevention of recurrent pericarditis and post-pericardiotomy syndrome: an updated meta-analysis of randomized clinical data. European heart journal Cardiovascular pharmacotherapy 2015, 1(2):117-125. |
| 11. Tian X, Zhang N, Korantzopoulos P et al. Efficacy and safety of colchicine for atrial fibrillation prevention: An updated meta-analysis of randomised controlled trials. International journal of cardiology 2024, 406:132068. |
| 12. Imazio M, Brucato A, Forno D et al. Efficacy and safety of colchicine for pericarditis prevention. Systematic review and meta-analysis. Heart (British Cardiac Society) 2012, 98^14^:1078-1082. |
| 13. Lotrionte M, Biondi-Zoccai G, Imazio M et al. International collaborative systematic review of controlled clinical trials on pharmacologic treatments for acute pericarditis and its recurrences. American heart journal 2010, 160(4):662-670. |
| 14. Lutschinger LL, Rigopoulos AG, Schlattmann P et al. Meta-analysis for the value of colchicine for the therapy of pericarditis and of postpericardiotomy syndrome. BMC cardiovascular disorders 2019, 19(1):207. |
| 15. Avondo S, Andreis A, Casula M et al. Pharmacologic treatment of acute and recurrent pericarditis: a systematic review and meta-analysis of controlled clinical trials. Panminerva medica 2021, 63(3):314-323. |
| 16. Raval J, Nagaraja V, Eslick GD et al. The role of colchicine in pericarditis - a systematic review and meta-analysis of randomised trials. Heart Lung and Circulation 2015, 24(7):660-666. |
| 17. Melendo-Viu M, Marchán-Lopez Á, Guarch CJ et al. A systematic review and meta-analysis of randomised controlled trials evaluating pharmacologic therapies for acute and recurrent pericarditis. Trends in cardiovascular medicine 2023, 33(5):319-326. |
| 18. Eun J, Smith A. Safety and efficacy of colchicine therapy in the prevention of recurrent pericarditis. American journal of health-system pharmacy : AJHP : official journal of the American Society of Health-System Pharmacists 2014, 7115:1277-1281. |
| 19. Giacinto O, Minati A, Lusini M et al. Treatment and Prophylaxis of Post-pericardiotomy Syndrome in Cardiac Surgery Patients: a Systematic Review. Cardiovascular drugs and therapy 2023, 37(4):771-779. |
| 20. Hernandez-Sómerson MA, Montoya-Agudelo F, Huertas-Rodriguez G. Efficacy and safety of drugs in residual cardiovascular risk: A systematic review of the literature. International journal of cardiology Cardiovascular risk and prevention 2024, 22:200298. |
| 21. Imazio M, Gaita F, LeWinter M. Evaluation and Treatment of Pericarditis: A Systematic Review. Jama 2015, 314^14^:1498-1506. |
| 22. Imazio M, Trinchero R, Shabetai R. Pathogenesis, management, and prevention of recurrent pericarditis. Journal of cardiovascular medicine (Hagerstown, Md) 2007, 8(6):404-410. |
| 23. Jacob KA, Nathoe HM, Dieleman JM et al. Inflammation in new-onset atrial fibrillation after cardiac surgery: a systematic review. European journal of clinical investigation 2014, 44(4):402-428. |
| 24. Sivera F, Wechalekar MD, Andrés M et al. Interleukin-1 inhibitors for acute gout. Cochrane Database of Systematic Reviews 2014, 2014(9). |
| 25. Sun H, Huang C, Li L et al. Time to benefit of colchicine in patients with cardiovascular disease: A pooled analysis of randomised controlled trials. Heliyon 2024, 10(9):e30408. |
| 26. Trout GO, Hoz RDL, Alfaro LM et al. Management of pericardial effusion: systematic review of literature. Revista Colombiana de Cardiologia 2018, 25(2):138-144. |

**Supplementary Table S4. The results of GRADE assessment of the evidence certainty on the association between colchicine intervention among patients with atherosclerotic cardiovascular disease**

| **Outcomes** | **Patients** | **Author, year,  reference** | **No. of  RCTs** | **Downgrade factors** | | | | | **Quality ^i^** |
| --- | --- | --- | --- | --- | --- | --- | --- | --- | --- |
|  |  |  |  | **Risk of bias** | **Indirectness** | **Inconsistency** | **Imprecision** | **Publication bias** |  |
| **Adverse events** |  |  |  |  |  |  |  |  |  |
| Adverse events | Surgery (aortic, CABG) | Wang et.al, 2022^1^ | 3 | No serious | No serious | No serious | No serious | Serious limitation ^h^ | Moderate |
| Adverse events | ACS | Abrantes et.al, 2021^2^ | 6 | No serious | No serious | Serious limitation ^e^ | No serious | Unclear | Moderate |
| Adverse events | CHD | Shrestha, 2022^3^ | 3 | No serious | No serious | No serious | No serious | No serious | High |
| Cutaneous adverse events | CHD | Andreis et.al, 2022^4^ | 3 | No serious | No serious | No serious | No serious | No serious | High |
| Diarrhea | ACS, CCS | Ma et.al, 2022^5^ | 5 | No serious | No serious | Serious limitation ^e^ | No serious | No serious | Moderate |
| Diarrhea | CHD | Xiang et.al, 2021^6^ | 5 | No serious | No serious | Serious limitation ^f^ | No serious | Unclear | Low |
| Drug discontinuation | CHD | Andreis et.al, 2021^7^ | 3 | No serious | No serious | Serious limitation ^e^ | No serious | No serious | Moderate |
| Drug discontinuation | CHD | Papageorgiou et.al, 2017^8^ | 5 | No serious | No serious | Serious limitation ^e^ | No serious | No serious | Moderate |
| Drug discontinuation | ACS, CCS | Grajek et.al, 2021^9^ | 10 | No serious | No serious | Serious limitation ^e^ | No serious | Unclear | Moderate |
| Drug discontinuation | CHD | Verma et.al, 2015^10^ | 3 | No serious | No serious | Serious limitation ^e^ | No serious | No serious | Moderate |
| Drug discontinuation | ACS, CCS | Grajek et.al, 2021^9^ | 4 | No serious | No serious | Serious limitation ^e^ | No serious | Unclear | Moderate |
| Drug discontinuation | CHD | Kofler et.al, 2021^11^ | 11 | No serious | No serious | Serious limitation ^f^ | No serious | No serious | Low |
| Drug discontinuation | CHD | Andreis et.al, 2021^7^ | 8 | No serious | No serious | No serious | No serious | No serious | High |
| Drug discontinuation | CHD | Andreis et.al, 2021^7^ | 4 | No serious | No serious | No serious | No serious | No serious | High |
| Drug discontinuation | CHD | Andreis et.al, 2021^7^ | 3 | No serious | No serious | No serious | No serious | No serious | High |
| Drug discontinuation | Surgery (aortic, CABG) | Agarwal et.al, 2023^12^ | 3 | No serious | No serious | No serious | No serious | No serious | High |
| Drug discontinuation (due to adverse events) | CHD | Verma et.al, 2015^10^ | 3 | No serious | No serious | Serious limitation ^e^ | No serious | No serious | Moderate |
| Drug discontinuation (without PROBE studies) | ACS, CCS | Grajek et.al, 2021^9^ | 8 | No serious | No serious | Serious limitation ^e^ | No serious | Unclear | Moderate |
| Gastrointestinal adverse events | CHD | Andreis et.al, 2022^4^ | 4 | No serious | No serious | No serious | No serious | No serious | High |
| Gastrointestinal adverse events | CHD | Shrestha, 2022^3^ | 9 | No serious | No serious | Serious limitation ^e^ | No serious | Serious limitation ^h^ | Low |
| Gastrointestinal adverse events | ACS, CCS | Abrantes et.al, 2021^2^ | 9 | Serious limitation ^c^ | No serious | Serious limitation ^e^ | No serious | Unclear | Low |
| Gastrointestinal adverse events | CHD | Verma et.al, 2015^10^ | 3 | No serious | No serious | No serious | No serious | No serious | High |
| Gastrointestinal adverse events | CHD | Xia et.al, 2021^13^ | 4 | No serious | No serious | No serious | No serious | Unclear | High |
| Gastrointestinal adverse events | CHD | Sen et.al, 2021^14^ | 3 | No serious | No serious | No serious | No serious | No serious | High |
| Gastrointestinal adverse events | ACS, CCS, surgery (PCI) | Xu et.al, 2022^15^ | 5 | No serious | No serious | No serious | No serious | No serious | High |
| Gastrointestinal adverse events | ACS, CCS | Ma et.al, 2022^5^ | 14 | No serious | No serious | Serious limitation ^e^ | No serious | No serious | Moderate |
| Gastrointestinal adverse events | CHD | Kofler et.al, 2021^11^ | 12 | No serious | No serious | Serious limitation ^f^ | No serious | No serious | Low |
| Gastrointestinal adverse events | CHD | Tien et.al, 2021^16^ | 8 | No serious | No serious | Serious limitation ^f^ | No serious | Unclear | Low |
| Gastrointestinal adverse events | CHD | Chen et.al, 2023^17^ | 12 | No serious | No serious | Serious limitation ^f^ | No serious | No serious | Low |
| Gastrointestinal adverse events | ACS | Bao et.al, 2022^18^ | 9 | No serious | No serious | Serious limitation ^e^ | No serious | Unclear | Moderate |
| Gastrointestinal adverse events | ACS | Younas et.al, 2024^19^ | 7 | No serious | No serious | Serious limitation ^f^ | No serious | No serious | Low |
| Gastrointestinal adverse events | ACS | Diaz-Arocutipa et.al, 2021^20^ | 5 | No serious | No serious | Serious limitation ^e^ | No serious | Unclear | Moderate |
| Gastrointestinal adverse events | CHD | Andreis et.al, 2021^7^ | 10 | No serious | No serious | Serious limitation ^e^ | No serious | No serious | Moderate |
| Gastrointestinal adverse events | ACS | Zhou et.al, 2023^21^ | 5 | No serious | No serious | Serious limitation ^e^ | No serious | No serious | Moderate |
| Gastrointestinal adverse events | CHD | Chen et.al, 2022^22^ | 10 | No serious | No serious | No serious | No serious | Unclear | High |
| Gastrointestinal adverse events | CHD | Andreis et.al, 2021^7^ | 4 | No serious | No serious | No serious | No serious | No serious | High |
| Gastrointestinal adverse events | CHD | Andreis et.al, 2021^7^ | 4 | No serious | No serious | No serious | No serious | No serious | High |
| Gastrointestinal adverse events | Surgery (aortic, CABG) | Agarwal et.al, 2023^12^ | 6 | No serious | No serious | Serious limitation ^e^ | No serious | No serious | Moderate |
| Gastrointestinal adverse events | Surgery (aortic, CABG) | Agarwal et.al, 2023^12^ | 5 | No serious | No serious | No serious | No serious | No serious | High |
| Gastrointestinal adverse events | CHD | Andreis et.al, 2022^4^ | 3 | No serious | No serious | No serious | No serious | Serious limitation ^h^ | Moderate |
| Gastrointestinal adverse events (surgery < 1 m) | Surgery (aortic, CABG) | Agarwal et.al, 2023^12^ | 3 | No serious | No serious | Serious limitation ^e^ | No serious | No serious | Moderate |
| Gastrointestinal adverse events (surgery ≥ 1 m) | Surgery (aortic, CABG) | Agarwal et.al, 2023^12^ | 3 | No serious | No serious | No serious | No serious | No serious | High |
| Hematology adverse events | CHD | Andreis et.al, 2022^4^ | 9 | No serious | No serious | No serious | No serious | No serious | High |
| Hepatic adverse events | CHD | Andreis et.al, 2022^4^ | 3 | No serious | No serious | No serious | No serious | No serious | High |
| Postoperative adverse events | PCI | Wei et.al, 2023^23^ | 5 | No serious | No serious | Serious limitation ^e^ | No serious | No serious | Moderate |
| ISR | ACS, surgery (PCI) | Aw et.al, 2022^24^ | 3 | No serious | No serious | Serious limitation ^e^ | No serious | Serious limitation ^h^ | Low |
| Myalgias | CHD | Andreis et.al, 2022^4^ | 3 | No serious | No serious | No serious | No serious | No serious | High |
| **All-cause and cause-specific mortality** |  |  |  |  |  |  |  |  |  |
| All-cause mortality | CHD | Akl et.al, 2024^25^ | 8 | No serious | No serious | Serious limitation ^e^ | No serious | No serious | Moderate |
| All-cause mortality | CHD | Verma et.al, 2015^10^ | 3 | No serious | No serious | No serious | No serious | No serious | High |
| All-cause mortality | ACS, AIS, surgery (PCI) | Masson et.al, 2020^26^ | 7 | No serious | No serious | No serious | No serious | No serious | High |
| All-cause mortality | ACS, CCS | Niu et.al, 2022^27^ | 3 | No serious | No serious | No serious | No serious | No serious | High |
| All-cause mortality | CHD | Sattar et.al, 2022^28^ | 5 | No serious | No serious | No serious | No serious | Unclear | High |
| All-cause mortality | CHD | Niu et.al, 2022^27^ | 4 | No serious | Serious limitation ^d^ | No serious | No serious | No serious | Moderate |
| All-cause mortality | CCS | Shrestha, 2022^3^ | 3 | No serious | No serious | Serious limitation ^e^ | No serious | No serious | Moderate |
| All-cause mortality | CHD, surgery (PCI) | Xu et.al, 2022^15^ | 5 | No serious | No serious | No serious | No serious | No serious | High |
| All-cause mortality | CHD, stroke | Fiolet et.al, 2024^29^ | 6 | No serious | No serious | No serious | No serious | No serious | High |
| All-cause mortality | CHD | Sen et.al, 2021^14^ | 4 | No serious | No serious | Serious limitation ^e^ | No serious | No serious | Moderate |
| All-cause mortality | ACS, CCS, surgery (PCI) | Aw et.al, 2022^24^ | 5 | No serious | No serious | No serious | No serious | Serious limitation ^h^ | Moderate |
| All-cause mortality | ACS | Wang et.al, 2021^30^ | 3 | No serious | Serious limitation ^d^ | No serious | No serious | No serious | Moderate |
| All-cause mortality | ACS, CCS | Grajek et.al, 2021^9^ | 8 | No serious | No serious | No serious | No serious | Unclear | High |
| All-cause mortality | ACS, CCS | Wang et.al, 2021^30^ | 4 | No serious | Serious limitation ^d^ | No serious | No serious | No serious | Moderate |
| All-cause mortality | ACS, CCS | Ma et.al, 2022^5^ | 13 | No serious | No serious | No serious | No serious | No serious | High |
| All-cause mortality | CHD | Kofler et.al, 2021^11^ | 13 | No serious | No serious | No serious | No serious | No serious | High |
| All-cause mortality | Surgery (PCI) | Fu etal, 2021^31^ | 4 | No serious | No serious | No serious | No serious | No serious | High |
| All-cause mortality | CHD | Liao et.al, 2021^32^ | 5 | No serious | No serious | No serious | No serious | Unclear | High |
| All-cause mortality | ACS | Bao et.al, 2022^18^ | 5 | No serious | No serious | No serious | No serious | Unclear | High |
| All-cause mortality | ACS | Younas et.al, 2024^19^ | 8 | No serious | No serious | No serious | No serious | No serious | High |
| All-cause mortality | ACS | Shrestha, 2022^3^ | 3 | No serious | No serious | No serious | No serious | No serious | High |
| All-cause mortality | ACS | Diaz-Arocutipa et.al, 2021^20^ | 5 | No serious | No serious | No serious | No serious | Unclear | High |
| All-cause mortality | CHD | Andreis et.al, 2022^4^ | 11 | No serious | No serious | No serious | No serious | Unclear | High |
| All-cause mortality | CHD | Chen et.al, 2022^22^ | 11 | No serious | No serious | No serious | No serious | Unclear | High |
| All-cause mortality | CHD | Xiang et.al, 2021^6^ | 8 | No serious | No serious | No serious | No serious | No serious | High |
| All-cause mortality | CHD | Bytyçi et.al, 2022^33^ | 9 | No serious | No serious | No serious | No serious | Unclear | High |
| All-cause mortality | ACS | Ullah et.al, 2021^34^ | 3 | No serious | No serious | No serious | No serious | No serious | High |
| All-cause mortality (excluding smaller studies) | ACS | Shrestha, 2022^3^ | 3 | No serious | No serious | Serious limitation ^e^ | No serious | No serious | Moderate |
| All-cause mortality (excluding the outlier study) | CCS | Shrestha, 2022^3^ | 3 | No serious | No serious | No serious | No serious | No serious | High |
| All-cause mortality (excluding the outlier study) | ACS | Shrestha, 2022^3^ | 3 | No serious | No serious | No serious | No serious | No serious | High |
| All-cause mortality (fixed-effect model) | CCS | Shrestha, 2022^3^ | 3 | No serious | No serious | Serious limitation ^e^ | No serious | No serious | Moderate |
| All-cause mortality (fixed-effect model) | ACS | Shrestha, 2022^3^ | 3 | No serious | No serious | No serious | No serious | No serious | High |
| All-cause mortality (follow-up time < 6 m) | CHD | Chen et.al, 2023^17^ | 3 | No serious | No serious | No serious | No serious | No serious | High |
| All-cause mortality (follow-up time ≥ 6 m) | CHD | Chen et.al, 2023^17^ | 6 | No serious | No serious | No serious | No serious | No serious | High |
| All-cause mortality (postoperative) | Surgery (PCI) | Wei et.al, 2023^23^ | 3 | No serious | No serious | No serious | No serious | No serious | High |
| All-cause mortality (pre- and post-operative) | Surgery (PCI) | Wei et.al, 2023^23^ | 3 | No serious | No serious | No serious | No serious | No serious | High |
| CV mortality | CHD | Kofler et.al, 2021^11^ | 9 | No serious | No serious | No serious | No serious | No serious | High |
| CV mortality | ACS | Ullah et.al, 2021^34^ | 5 | No serious | No serious | No serious | No serious | No serious | High |
| CV mortality | ACS | Diaz-Arocutipa et.al, 2021^20^ | 4 | No serious | No serious | No serious | No serious | Unclear | High |
| CV mortality | CHD | Akl et.al, 2024^25^ | 8 | No serious | No serious | No serious | No serious | No serious | High |
| CV mortality | ACS, CCS | Ma et.al, 2022^5^ | 9 | No serious | No serious | No serious | No serious | Serious limitation ^h^ | Moderate |
| CV mortality | ACS, AIS, surgery (PCI) | Masson et.al, 2020^26^ | 6 | No serious | No serious | Serious limitation ^e^ | No serious | No serious | Moderate |
| CV mortality | ACS, CCS | Grajek et.al, 2021^9^ | 6 | No serious | No serious | No serious | No serious | Unclear | High |
| CV mortality | ACS, CCS | Niu et.al, 2022^27^ | 3 | No serious | No serious | No serious | No serious | Serious limitation ^h^ | Moderate |
| CV mortality | CHD | Sattar et.al, 2022^28^ | 4 | No serious | No serious | No serious | No serious | Unclear | High |
| CV mortality | ACS, CCS | Abrantes et.al, 2021^2^ | 6 | No serious | No serious | No serious | No serious | Unclear | High |
| CV mortality | CHD | Xia et.al, 2021^13^ | 5 | No serious | No serious | No serious | No serious | Unclear | High |
| CV mortality | CHD | Niu et.al, 2022^27^ | 4 | No serious | No serious | No serious | No serious | Serious limitation ^h^ | Moderate |
| CV mortality | ACS, CCS | Grajek et.al, 2021^9^ | 5 | No serious | No serious | No serious | No serious | Unclear | High |
| CV mortality | CCS | Shrestha, 2022^3^ | 3 | No serious | No serious | Serious limitation ^e^ | No serious | No serious | Moderate |
| CV mortality | ACS, CCS | Samuel et.al, 2021^35^ | 3 | No serious | No serious | No serious | No serious | No serious | High |
| CV mortality | CHD | Sen et.al, 2021^14^ | 4 | No serious | No serious | No serious | No serious | No serious | High |
| CV mortality | ACS, CCS, surgery (PCI) | Xu et.al, 2022^15^ | 6 | No serious | No serious | No serious | No serious | No serious | High |
| CV mortality | CHD, stroke | Fiolet et.al, 2024^29^ | 6 | No serious | No serious | No serious | No serious | No serious | High |
| CV mortality | CHD | Chen et.al, 2023^17^ | 4 | No serious | No serious | No serious | No serious | No serious | High |
| CV mortality | ACS | Wang et.al, 2021^30^ | 3 | No serious | Serious limitation ^d^ | No serious | No serious | No serious | Moderate |
| CV mortality | ACS, CCS | Wang et.al, 2021^30^ | 4 | No serious | Serious limitation ^d^ | No serious | No serious | No serious | Moderate |
| CV mortality | CHD | Chen et.al, 2022^22^ | 9 | No serious | No serious | No serious | No serious | Unclear | High |
| CV mortality | ACS | Bao et.al, 2022^18^ | 4 | No serious | No serious | No serious | No serious | Unclear | High |
| CV mortality | CHD | Andreis et.al, 2022^4^ | 9 | No serious | No serious | No serious | No serious | Unclear | High |
| CV mortality | CHD | Bytyçi et.al, 2022^33^ | 7 | No serious | No serious | No serious | No serious | Unclear | High |
| CV mortality (fixed-effect model) | ACS | Shrestha, 2022^3^ | 3 | No serious | No serious | Serious limitation ^e^ | No serious | No serious | Moderate |
| Non-CV mortality | CHD | Akl et.al, 2024^25^ | 8 | No serious | No serious | Serious limitation ^e^ | No serious | No serious | Moderate |
| Non-CV mortality | ACS, CCS | Ma et.al, 2022^5^ | 7 | No serious | No serious | No serious | No serious | No serious | High |
| Non-CV mortality | CHD | Andreis et.al, 2021^7^ | 3 | No serious | No serious | No serious | No serious | No serious | High |
| Non-CV mortality | CHD | Sen et.al, 2021^14^ | 4 | No serious | No serious | No serious | No serious | No serious | High |
| Non-CV mortality | CHD, stroke | Fiolet et.al, 2024^29^ | 5 | No serious | No serious | No serious | No serious | No serious | High |
| Non-CV mortality | ACS, CCS | Xu et.al, 2022^15^ | 4 | No serious | No serious | No serious | No serious | No serious | High |
| Non-CV mortality | CHD | Xia et.al, 2021^13^ | 4 | No serious | No serious | No serious | No serious | Unclear | High |
| Non-CV mortality | CHD | Chen et.al, 2022^22^ | 9 | No serious | No serious | No serious | No serious | Unclear | High |
| **Cardiovascular disorders** |  |  |  |  |  |  |  |  |  |
| ACS | ACS | Ullah et.al, 2021^34^ | 6 | No serious | No serious | No serious | No serious | No serious | High |
| ACS | ACS, CCS | Abrantes et.al, 2021^2^ | 7 | No serious | No serious | No serious | No serious | Unclear | High |
| ACS | CHD | Chen et.al, 2023^17^ | 5 | No serious | No serious | No serious | No serious | No serious | High |
| ACS | ACS, CCS | Akl et.al, 2024^25^ | 5 | No serious | No serious | No serious | No serious | No serious | High |
| ACS | CHD | Shrestha, 2022^3^ | 3 | No serious | No serious | No serious | No serious | No serious | High |
| ACS | ACS | Alberto et.al, 2021^36^ | 5 | No serious | No serious | No serious | No serious | Unclear | High |
| ACS (new) | CHD | Chen et.al, 2023^17^ | 8 | No serious | No serious | No serious | No serious | No serious | High |
| ACS or UA | CHD | Shrestha, 2022^3^ | 3 | No serious | No serious | No serious | No serious | No serious | High |
| ACS or UA (fixed-effect model) | CHD | Shrestha, 2022^3^ | 3 | No serious | No serious | No serious | No serious | No serious | High |
| CHD | CHD | Xu et.al, 2022^15^ | 3 | No serious | No serious | Serious limitation ^e^ | No serious | No serious | Moderate |
| CHD | CCS | Casula et.al, 2022^37^ | 3 | No serious | No serious | Serious limitation ^e^ | No serious | No serious | Moderate |
| CHD | CHD | Andreis et.al, 2021^7^ | 8 | No serious | No serious | No serious | No serious | No serious | High |
| CCS | CCS | Alberto et.al, 2021^36^ | 3 | No serious | No serious | Serious limitation ^e^ | No serious | Unclear | Moderate |
| Cardiac arrest | ACS | Younas et.al, 2024^19^ | 3 | No serious | No serious | No serious | No serious | No serious | High |
| MI | CHD | Sattar et.al, 2022^28^ | 5 | No serious | No serious | No serious | No serious | Unclear | High |
| MI | CHD | Akl et.al, 2024^25^ | 6 | No serious | No serious | No serious | No serious | No serious | High |
| MI | ACS, AIS, surgery (PCI) | Masson et.al, 2020^26^ | 5 | No serious | No serious | Serious limitation ^e^ | No serious | No serious | Moderate |
| MI | ACS | Niu et.al, 2022^27^ | 3 | No serious | No serious | No serious | No serious | Serious limitation ^h^ | Moderate |
| MI | CHD | Niu et.al, 2022^27^ | 4 | No serious | No serious | No serious | No serious | Serious limitation ^h^ | Moderate |
| MI | CHD | Niu et.al, 2022^27^ | 3 | No serious | No serious | No serious | No serious | Serious limitation ^h^ | Moderate |
| MI | ACS, CHD | Samuel et.al, 2021^35^ | 4 | No serious | No serious | Serious limitation ^e^ | No serious | No serious | Moderate |
| MI | CHD | Al-Atta et.al, 2021^38^ | 4 | Unclear | No serious | Serious limitation ^e^ | No serious | No serious | Moderate |
| MI | CHD | Xia et.al, 2021^13^ | 4 | No serious | No serious | No serious | No serious | Unclear | High |
| MI | ACS, CCS | Grajek et.al, 2021^9^ | 4 | No serious | No serious | No serious | No serious | Unclear | High |
| MI | CHD | Andreis et.al, 2022^4^ | 7 | No serious | No serious | No serious | No serious | Unclear | High |
| MI | ACS, CCS | Grajek et.al, 2021^9^ | 7 | No serious | No serious | No serious | No serious | Unclear | High |
| MI | ACS, CCS | Ma et.al, 2022^5^ | 9 | No serious | No serious | Serious limitation ^e^ | No serious | No serious | Moderate |
| MI | CHD | Chen et.al, 2022^22^ | 7 | No serious | No serious | No serious | No serious | Unclear | High |
| MI | ACS | Zhou et.al, 2023^21^ | 4 | No serious | No serious | No serious | No serious | No serious | High |
| MI (postoperative) | PCI | Wei et.al, 2023^23^ | 3 | No serious | No serious | No serious | No serious | No serious | High |
| MI (acute) | CHD | Shrestha, 2022^3^ | 3 | No serious | No serious | No serious | No serious | No serious | High |
| MI (acute) | CHD | Xiang et.al, 2021^6^ | 4 | No serious | No serious | No serious | No serious | Unclear | High |
| MI (recurrent) | ACS | Diaz-Arocutipa et.al, 2021^20^ | 4 | No serious | No serious | No serious | No serious | Unclear | High |
| MI (recurrent) | CHD | Bytyçi et.al, 2022^33^ | 7 | No serious | No serious | No serious | No serious | Unclear | High |
| MI (recurrent) | ACS | Wang et.al, 2021^30^ | 4 | No serious | Serious limitation ^d^ | No serious | No serious | No serious | Moderate |
| MI (recurrent) | ACS, CCS | Wang et.al, 2021^30^ | 5 | No serious | Serious limitation ^d^ | No serious | No serious | No serious | Moderate |
| MI (recurrent) | ACS | Bao et.al, 2022^18^ | 7 | No serious | No serious | No serious | No serious | Unclear | High |
| MI (recurrent) | ACS | Younas et.al, 2024^19^ | 6 | No serious | No serious | No serious | No serious | No serious | High |
| POAF | Surgery (aortic, CABG) | Ge et.al, 2022^39^ | 5 | No serious | No serious | No serious | No serious | No serious | High |
| POAF | Surgery (aortic, CABG) | Wang et.al, 2022^1^ | 4 | No serious | No serious | No serious | No serious | Serious limitation ^h^ | Moderate |
| POAF | Surgery (CABG) | Kirov et.al, 2024^40^ | 5 | No serious | No serious | No serious | No serious | No serious | High |
| POAF | Surgery (aortic, CABG, MR) | Zhao et.al, 2022^41^ | 5 | No serious | No serious | No serious | No serious | No serious | High |
| POAF | CHD | Teo et.al, 2021^42^ | 4 | No serious | No serious | No serious | No serious | Unclear | High |
| POAF | Surgery (aortic, CABG) | Agarwal et.al, 2023^12^ | 8 | No serious | No serious | No serious | No serious | No serious | High |
| POAF (< 1 m) | Surgery (aortic, CABG, MR) | Zhao et.al, 2022^41^ | 5 | No serious | No serious | No serious | No serious | No serious | High |
| POAF (< 1 m) | Surgery (aortic, CABG, MR) | Agarwal et.al, 2023^12^ | 5 | No serious | No serious | No serious | No serious | No serious | High |
| POAF (≥ 1 m) | Surgery (aortic, CABG) | Agarwal et.al, 2023^12^ | 3 | No serious | No serious | No serious | No serious | No serious | High |
| POAF (no history of AF) | Surgery (aortic, CABG) | Agarwal et.al, 2023^12^ | 4 | No serious | No serious | No serious | No serious | No serious | High |
| **Hospitalization** |  |  |  |  |  |  |  |  |  |
| Hospitalization | ACS, CCS | Abrantes et.al, 2021^2^ | 4 | No serious | No serious | Serious limitation ^e^ | No serious | Unclear | Moderate |
| Hospitalization | CHD | Bytyçi et.al, 2022^33^ | 7 | No serious | No serious | No serious | No serious | Unclear | High |
| Hospitalization urgency | ACS | Younas et.al, 2024^19^ | 3 | No serious | No serious | No serious | No serious | No serious | High |
| Rehospitalization | CHD | Shrestha, 2022^3^ | 4 | No serious | No serious | Serious limitation ^e^ | No serious | No serious | Moderate |
| Rehospitalisation (excluding the outlier study) | CHD | Shrestha, 2022^3^ | 3 | No serious | No serious | No serious | No serious | No serious | High |
| **MACEs** |  |  |  |  |  |  |  |  |  |
| ACS, AF, mortality, revascularization and stroke | Surgery (CABG, PCI) | Chen et.al, 2023^17^ | 7 | Serious limitation ^c^ | No serious | No serious | No serious | No serious | Moderate |
| ACS, CHD, post-angioplasty and stroke | CHD | Verma et.al, 2015^10^ | 4 | No serious | No serious | No serious | No serious | No serious | High |
| Adverse cardiovascular events a | ACS | Younas et.al, 2024^19^ | 7 | No serious | No serious | No serious | No serious | No serious | High |
| All-cause mortality, cardiac arrest, ISR, MI, stent thrombosis and stroke | Surgery (PCI) | Wei et.al, 2023^23^ | 5 | No serious | No serious | No serious | No serious | No serious | High |
| All-cause mortality, cardiac arrest, ISR, MI, stent thrombosis and stroke | Surgery (PCI) | Wei et.al, 2023^23^ | 5 | No serious | No serious | No serious | No serious | No serious | High |
| All-cause mortality and stroke (post surgery) | Surgery (aortic, CABG, MR) | Ge et.al, 2022^39^ | 6 | No serious | No serious | No serious | No serious | No serious | High |
| All-cause mortality, CV mortality, MI and stroke | ACS, CCS | Abrantes et.al, 2021^2^ | 6 | No serious | No serious | No serious | No serious | Unclear | High |
| All-cause mortality, CV mortality, MI and stroke | CHD | Xia et.al, 2021^13^ | 5 | No serious | No serious | No serious | No serious | No serious | High |
| All-cause mortality, CV mortality, recurrent MI and stroke | CHD | Bytyçi et.al, 2022^33^ | 7 | No serious | No serious | No serious | No serious | Unclear | High |
| All-cause mortality, ACS, CV mortality, revascularization and stroke | CHD | Shrestha, 2022^3^ | 4 | No serious | No serious | Serious limitation ^e^ | No serious | No serious | Moderate |
| All-cause mortality, CV mortality and stroke | CHD | Aw et.al, 2022^24^ | 7 | No serious | No serious | No serious | No serious | Serious limitation ^h^ | Moderate |
| All-cause mortality, HF, MI, revascularization and stroke | ACS | Zhou et.al, 2023^21^ | 3 | No serious | No serious | No serious | No serious | No serious | High |
| All-cause mortality, HF, MI, revascularization and stroke | ACS | Zhou et.al, 2023^21^ | 5 | No serious | No serious | No serious | No serious | No serious | High |
| All-cause mortality, HF, MI, revascularization and stroke | ACS | Zhou et.al, 2023^21^ | 3 | No serious | No serious | No serious | No serious | No serious | High |
| All-cause mortality, HF, MI, revascularization and stroke (≤ 3 d) | ACS | Zhou et.al, 2023^21^ | 5 | No serious | No serious | No serious | No serious | No serious | High |
| All-cause mortality, recurrent MI and stroke | ACS, CCS | Wang et.al, 2021^30^ | 4 | No serious | Serious limitation ^d^ | No serious | No serious | No serious | Moderate |
| All-cause mortality, recurrent MI, revascularization and stroke | ACS, CCS | Wang et.al, 2021^30^ | 3 | No serious | Serious limitation ^d^ | No serious | No serious | No serious | Moderate |
| CV mortality, coronary revascularization, MI and stroke | Stroke, TIA | Fiolet et.al, 2024^29^ | 3 | No serious | No serious | No serious | No serious | No serious | High |
| CV mortality, coronary revascularization, MI and stroke | CHD | Condello et.al, 2021^43^ | 3 | No serious | Serious limitation ^d^ | Serious limitation ^e^ | No serious | No serious | Low |
| CV mortality, coronary revascularization, MI and stroke | ACS, CCS, surgery (PCI) | Xu et.al, 2022^15^ | 5 | No serious | No serious | Serious limitation ^e^ | No serious | No serious | Moderate |
| CV mortality, coronary revascularization, MI and stroke | CHD | Teo et.al, 2021^42^ | 3 | No serious | No serious | No serious | No serious | Unclear | High |
| CV mortality, coronary revascularization, MI and stroke | CHD | Masson et.al, 2021^44^ | 4 | No serious | Serious limitation ^d^ | No serious | No serious | No serious | Moderate |
| CV mortality, coronary revascularization, MI and stroke | ACS, CCS | Samuel et.al, 2021^35^ | 3 | No serious | No serious | No serious | No serious | No serious | High |
| CV mortality, coronary revascularization, MI and stroke | ACS, CCS, surgery (PCI) | Xu et.al, 2022^15^ | 7 | No serious | No serious | No serious | No serious | No serious | High |
| CV mortality, coronary revascularization, MI and stroke | CHD, stroke | Fiolet et.al, 2024^29^ | 5 | No serious | No serious | No serious | No serious | No serious | High |
| CV mortality, coronary revascularization, MI and stroke | CHD | Al-Atta et.al, 2021^38^ | 4 | Unclear | No serious | Serious limitation ^e^ | No serious | No serious | Moderate |
| CV mortality, MI and revascularization | CHD | Liao et.al, 2021^32^ | 3 | No serious | No serious | No serious | No serious | Unclear | High |
| CV mortality, MI and stroke | CHD | Akl et.al, 2024^25^ | 5 | No serious | No serious | Serious limitation ^e^ | No serious | No serious | Moderate |
| CV mortality, MI and stroke | CHD | Akl et.al, 2024^25^ | 3 | Serious limitation ^c^ | No serious | No serious | Serious limitation ^g^ | No serious | Low |
| CV mortality, MI and stroke | ACS, CCS | Ma et.al, 2022^5^ | 7 | No serious | No serious | Serious limitation ^e^ | No serious | No serious | Moderate |
| CV mortality, MI and stroke | CHD | Akl et.al, 2024^25^ | 3 | No serious | No serious | No serious | Serious limitation ^g^ | No serious | Moderate |
| CV mortality, MI and stroke | CHD | Akl et.al, 2024^25^ | 5 | Serious limitation ^c^ | No serious | Serious limitation ^e^ | No serious | No serious | Low |
| CV mortality, MI and stroke | ACS, CCS | Akl et.al, 2024^25^ | 6 | No serious | No serious | Serious limitation ^e^ | No serious | No serious | Moderate |
| CV mortality, MI and stroke | Atherosclerosis | Fiolet et.al, 2021^45^ | 4 | No serious | No serious | No serious | No serious | Unclear | High |
| CV mortality, MI,PCI, revascularization and stroke | ACS, CCS | Grajek et.al, 2021^9^ | 5 | No serious | No serious | Serious limitation ^e^ | No serious | Unclear | Moderate |
| Major CV events **^b^** | CHD with diabetes | Kuzemczak et.al, 2021^46^ | 4 | Unclear | No serious | No serious | No serious | No serious | High |
| Major CV events **^b^** | CHD without diabetes | Kuzemczak et.al, 2021^46^ | 4 | Unclear | No serious | No serious | No serious | No serious | High |
| Major adverse CV and cerebrovascular events | CHD | Andreis et.al, 2022^4^ | 11 | No serious | No serious | No serious | No serious | Unclear | High |
| **Revascularization** |  |  |  |  |  |  |  |  |  |
| Revascularization | CHD | Teo et.al, 2021^42^ | 3 | No serious | No serious | Serious limitation ^e^ | No serious | Unclear | Moderate |
| Revascularization | CHD | Xiang et.al, 2021^28^ | 4 | No serious | No serious | No serious | No serious | Unclear | High |
| Revascularization | ACS, CCS | Akl et.al, 2024^25^ | 4 | No serious | No serious | Serious limitation ^e^ | No serious | No serious | Moderate |
| Revascularization | ACS, CCS | Grajek et.al, 2021^9^ | 6 | No serious | No serious | No serious | No serious | Unclear | High |
| Revascularization | Atherosclerosis | Fiolet et.al, 2021^45^ | 5 | No serious | No serious | No serious | No serious | Unclear | High |
| Revascularization | CHD | Chen et.al, 2023^17^ | 7 | No serious | No serious | No serious | No serious | No serious | High |
| Revascularization | CHD | Chen et.al, 2022^22^ | 4 | No serious | No serious | No serious | No serious | Unclear | High |
| Revascularization | ACS, CCS | Abrantes et.al, 2021^2^ | 4 | No serious | No serious | No serious | No serious | Unclear | High |
| Revascularization | ACS, CCS | Grajek et.al, 2021^9^ | 5 | No serious | No serious | No serious | No serious | Unclear | High |
| Revascularization | ACS, CCS | Samuel et.al, 2021^35^ | 3 | No serious | No serious | Serious limitation ^e^ | No serious | Serious limitation ^h^ | Low |
| Revascularization | CHD | Niu et.al, 2022^27^ | 3 | No serious | No serious | No serious | No serious | No serious | High |
| Revascularization | ACS, CCS, surgery (PCI) | Xu et.al, 2022^15^ | 5 | No serious | No serious | No serious | No serious | No serious | High |
| Revascularization | CHD | Sattar et.al, 2022^28^ | 3 | No serious | No serious | Serious limitation ^e^ | No serious | Unclear | Moderate |
| Revascularization | ACS, CCS | Wang et.al, 2021^30^ | 3 | No serious | Serious limitation ^d^ | No serious | No serious | No serious | Moderate |
| Revascularization | ACS | Bao et.al, 2022^18^ | 3 | No serious | No serious | No serious | No serious | Unclear | High |
| Revascularization | CHD | Andreis et.al, 2022^4^ | 5 | No serious | No serious | No serious | No serious | Unclear | High |
| Repeat vessel revascularization | CHD | Aw et.al, 2022^24^ | 4 | No serious | No serious | No serious | No serious | Serious limitation ^h^ | Moderate |
| Restenosis after PCI | Surgery (PCI) | Tien et.al, 2021^16^ | 3 | No serious | No serious | Serious limitation ^e^ | Serious limitation ^g^ | Unclear | Low |
| Revascularization (fixed-effect model) | CHD | Shrestha, 2022^3^ | 4 | No serious | No serious | No serious | No serious | No serious | High |
| Stent thrombosis | CHD | Aw et.al, 2022^24^ | 3 | No serious | No serious | No serious | No serious | Serious limitation ^h^ | Moderate |
| **Stroke** |  |  |  |  |  |  |  |  |  |
| Ischaemic stroke | CHD | Xiang et.al, 2021^6^ | 5 | No serious | No serious | No serious | No serious | Unclear | High |
| Ischaemic stroke | ACS, CCS | Samuel et.al, 2021^35^ | 4 | No serious | No serious | No serious | No serious | No serious | High |
| Ischaemic stroke | CHD, stroke | Fiolet et.al, 2024^29^ | 5 | No serious | No serious | No serious | No serious | No serious | High |
| Ischaemic stroke | CHD | Chen et.al, 2022^22^ | 5 | No serious | No serious | No serious | No serious | Unclear | High |
| Non-cardio-embolic ischaemic stroke | CHD | Shrestha, 2022^3^ | 6 | No serious | No serious | No serious | No serious | No serious | High |
| Stroke | ACS, CCS | Ullah et.al, 2021^34^ | 3 | No serious | No serious | No serious | No serious | No serious | High |
| Stroke | CCS | Katsanos et.al, 2021^47^ | 3 | No serious | No serious | No serious | No serious | No serious | High |
| Stroke | ACS, CCS | Ma et.al, 2022^5^ | 6 | No serious | No serious | No serious | No serious | No serious | High |
| Stroke | ACS, AIS | Katsanos et.al, 2021^47^ | 3 | No serious | No serious | No serious | No serious | No serious | High |
| Stroke | ACS, CCS | Abrantes et.al, 2021^2^ | 6 | No serious | No serious | No serious | No serious | Unclear | High |
| Stroke | CHD | Katsanos et.al, 2020^48^ | 4 | No serious | No serious | No serious | No serious | Unclear | High |
| Stroke | ACS, CHD | Akl et.al, 2024^25^ | 8 | No serious | No serious | No serious | No serious | No serious | High |
| Stroke | ACS, AIS, CCS | Masson et.al, 2020^26^ | 5 | No serious | No serious | No serious | No serious | No serious | High |
| Stroke | Surgery (PCI) | Wei et.al, 2023^23^ | 3 | No serious | No serious | No serious | No serious | No serious | High |
| Stroke | CHD | Niu et.al, 2022^27^ | 3 | No serious | No serious | No serious | No serious | Serious limitation ^h^ | Moderate |
| Stroke | ACS, CCS | Xu et.al, 2022^15^ | 5 | No serious | No serious | No serious | No serious | No serious | High |
| Stroke | CHD | Al-Atta et.al, 2021^38^ | 4 | Unclear | No serious | No serious | No serious | No serious | High |
| Stroke | CHD | Sattar et.al, 2022^28^ | 4 | No serious | No serious | No serious | No serious | Unclear | High |
| Stroke | ACS, CCS | Grajek et.al, 2021^9^ | 5 | No serious | No serious | No serious | No serious | Unclear | High |
| Stroke | ACS, CCS | Ullah et.al, 2021^34^ | 4 | No serious | No serious | No serious | No serious | No serious | High |
| Stroke | CHD | Aw et.al, 2022^24^ | 7 | No serious | No serious | No serious | No serious | Serious limitation ^h^ | Moderate |
| Stroke | ACS | Wang et.al, 2021^30^ | 3 | No serious | Serious limitation ^d^ | No serious | No serious | No serious | Moderate |
| Stroke | CHD | Chen et.al, 2023^17^ | 7 | No serious | No serious | No serious | No serious | No serious | High |
| Stroke | ACS, CCS | Wang et.al, 2021^30^ | 5 | No serious | Serious limitation ^d^ | No serious | No serious | No serious | Moderate |
| Stroke | ACS | Bao et.al, 2022^18^ | 5 | No serious | No serious | No serious | No serious | Unclear | High |
| Stroke | CHD | Andreis et.al, 2022^4^ | 7 | No serious | No serious | No serious | No serious | Unclear | High |
| Stroke | ACS | Younas et.al, 2024^19^ | 4 | No serious | No serious | No serious | No serious | No serious | High |
| Stroke | CHD | Bytyçi et.al, 2022^33^ | 7 | No serious | No serious | No serious | No serious | Unclear | High |
| **Other Outcomes** |  |  |  |  |  |  |  |  |  |
| Cancer | CHD | Sen et.al, 2021^14^ | 3 | No serious | No serious | No serious | No serious | No serious | High |
| CRP | ACS | Zhou et.al, 2023^21^ | 4 | No serious | No serious | Serious limitation ^f^ | No serious | No serious | Low |
| CRP | ACS | Zhou et.al, 2023^21^ | 5 | No serious | No serious | Serious limitation ^f^ | No serious | No serious | Low |
| CRP | ACS | Zhou et.al, 2023^21^ | 8 | No serious | No serious | Serious limitation ^e^ | No serious | No serious | Moderate |
| Hs-CRP | ACS, CCS | Grajek et.al, 2021^9^ | 6 | No serious | No serious | Serious limitation ^f^ | No serious | Unclear | Low |
| Hs-CRP | ACS | Younas et.al, 2024^19^ | 6 | No serious | No serious | Serious limitation ^e^ | No serious | No serious | Moderate |
| Hs-CRP | ACS | Diaz-Arocutipa et.al, 2021^20^ | 4 | No serious | No serious | Serious limitation ^e^ | No serious | Unclear | Moderate |
| Infection | CHD | Chen et.al, 2023^17^ | 3 | No serious | No serious | Serious limitation ^f^ | No serious | No serious | Low |
| Infection | CHD | Teo et.al, 2021^42^ | 3 | No serious | No serious | Serious limitation ^e^ | No serious | Unclear | Moderate |
| Leukocytes | ACS | Zhou et.al, 2023^21^ | 3 | No serious | No serious | Serious limitation ^f^ | No serious | No serious | Low |
| LVEF | ACS | Zhou et.al, 2023^21^ | 3 | No serious | No serious | Serious limitation ^e^ | No serious | No serious | Moderate |
| Neutrophils | ACS | Zhou et.al, 2023^21^ | 4 | No serious | No serious | No serious | No serious | No serious | High |
| Pneumonia | CHD | Sen et.al, 2021^14^ | 3 | No serious | No serious | Serious limitation ^e^ | No serious | No serious | Moderate |
| Pneumonia | Atherosclerosis | Fiolet et.al, 2021^45^ | 3 | No serious | No serious | Serious limitation ^e^ | No serious | Unclear | Moderate |
| Pneumonia | CHD | Chen et.al, 2023^17^ | 3 | No serious | No serious | Serious limitation ^e^ | No serious | No serious | Moderate |
| ACS, acute coronary syndrome; AF, atrial fibrillation; AIS, acute ischaemic stroke; CABG, coronary artery bypass grafting; CCS, chronic coronary syndromes; CHD, coronary heart disease; CI, confidence interval; CRP, C-reactive protein; CV, cardiovascular; d, day; HF, heart failure; Hs-CRP, high-sensitive C-reactive protein; ISR, in-stent restenosis; LVEF, left ventricular ejection fraction; m, month; MACEs, major adverse cardiac events; MI, myocardial infarction; MR, myocardial revascularization; NA, not available; No., number; PCI, percutaneous coronary intervention; POAF, postoperative atrial fibrillation; PROBE, Prospective, Randomized, Open-label, Blinded Endpoint; RCTs, randomised controlled trials; TIA, transient ischaemic attack; UA, unstable angina. | | | | | | | | | |
| ^a^ Adverse cardiovascular events: ACS, CV mortality, HF, MI, resuscitated cardiac arrest, stroke, UA, urgent hospitalization for angina and ventricular arrhythmias. | | | | | | | | | |
| ^b^ Major CV events: ACS, out-of-hospital cardiac arrest, CV mortality, resuscitated cardiac arrest, MI, stroke, or urgent hospitalization for angina, leading to coronary revascularization, ischaemic stroke, or ischemia-driven coronary revascularization. | | | | | | | | | |
| ^c^ Downgraded by one level for the risk of bias: more than 50% of the studies were of high risk of bias according to Cochrane quality assessment tool for randomized clinical trials. | | | | | | | | | |
| ^d^ Downgraded by one level for indirectness: there were differences in population, intervention, comparison and outcome among different associations of the included original studies. | | | | | | | | | |
| ^e^ Downgraded by one level for inconsistency: *I^2^* > 50% or *I^2^* > 75% and heterogeneity was mainly explained. | | | | | | | | | |
| ^f^ Downgraded by two levels for inconsistency: *I*^2^ > 75% and heterogeneity was not explained. | | | | | | | | | |
| ^g^ Downgraded by one level for imprecision: the number of cases < 200. | | | | | | | | | |
| ^h^ Downgraded by one level for publication bias: asymmetry on funnel plot, the *P* of Deek test < 0.1, the *P* of Egger' s test or Begg' s test < 0.05. | | | | | | | | | |
| ^i^ Since all the included studies were meta-analyses of randomized clinical trials, the certainty of evidence for all outcomes was rated as high by default, and then downgraded according to prespecified criteria. The quality was rated as high, moderate, low, and very low. | | | | | | | | | |

**Supplementary Table S5. Sensitivity analysis results of excluded meta-analyses due to overlap in randomised controlled trials studies**

| **Outcomes** | **Intervention Dose (mg/d)** | **Intervention Duration (month)** | **Comparison** | **Patients** | **Author, year,  reference** | **Primary analysis** | | | **Sensitivity analysis** | | |
| --- | --- | --- | --- | --- | --- | --- | --- | --- | --- | --- | --- |
|  |  |  |  |  |  | **No. of RCTs** | **Random effect  size (95% CI)** | **GRADE** | **No. of RCTs** | **Random effect  size (95% CI)** | **GRADE** |
| Adverse events | 0.5 - 1.0 | 1.0 - 24.0 | Placebo | ACS | Abrantes et.al, 2021^2^ | 6 | 1.10 (0.90, 1.35) | Moderate | 3 | 0.97 (0.89, 1.07) | High |
| All-cause mortality | 0.5 - 1.0 | 1.0 - 28.6 | Placebo | CHD | Teo et.al, 2021^42^ | 3 | 1.20 (0.76, 1.90) | Moderate | 3 | 1.20 (0.76, 1.90) | Moderate |
| All-cause mortality | 0.5 - 1.0 | 6.0 - 28.6 | Placebo | CHD | Niu et.al, 2022^27^ | 10 | 1.00 (0.94, 1.07) | Moderate | 4 | 1.00 (0.91, 1.09) | Moderate |
| All-cause mortality | 0.5 - 1.0 | 6.0 - 36.0 | Placebo | CHD | Grajek et.al, 2021^9^ | 6 | 1.09 (0.89, 1.33) | High | 5 | 1.04 (0.64, 1.69) | High |
| All-cause mortality | 0.5 - 1.0 | 7.1 - 36.0 | Placebo | CHD | Al-Atta et.al, 2021^38^ | 6 | 1.09 (0.89, 1.33) | High | 4 | 1.04 (0.61, 1.78) | Moderate |
| All-cause mortality | 0.5 - 1.0 | 7.1 - 36.0 | Placebo | CHD | Samuel et.al, 2021^35^ | 6 | 1.09 (0.89, 1.33) | High | 4 | 1.04 (0.61, 1.78) | Moderate |
| All-cause mortality | 0.5 - 2.0 | 0.2 - 28.6 | Placebo | CHD | Andreis et.al, 2021^7^ | 11 | 1.01 (0.71, 1.43) | High | 5 | 1.14 (0.77, 1.68) | High |
| All-cause mortality | 0.5 - 2.0 | 0.2 - 36.0 | Placebo | CHD | Tien et.al, 2021^16^ | 9 | 0.80 (0.56, 1.15) | High | 9 | 0.80 (0.56, 1.15) | High |
| All-cause mortality | 0.5 - 2.0 | 0.2 - 36.0 | Placebo | CHD | Xiang et.al, 2021^6^ | 11 | 1.09 (0.85, 1.40) | High | 5 | 1.03 (0.80, 1.32) | High |
| All-cause mortality (excluding the outlier study) | NA | NA | Placebo | ACS, AIS | Masson et al, 2020^43^ | 6 | 1.09 (0.89, 1.33) | High | 5 | 0.33 (0.15, 0.72) | High |
| All-cause mortality, recurrent MI and stroke | 0.5 - 1.8 | < 0.1 - 36.0 | Placebo | CHD | Wang et.al, 2021^30^ | 4 | 0.81 (0.70, 0.95) | Moderate | 4 | 0.81 (0.70, 0.95) | Moderate |
| Cancer | 0.5 - 1.0 | 12.0 - 24.0 | Placebo | CHD | Fiolet et.al, 2021^45^ | 3 | 0.98 (0.80, 1.21) | High | 3 | 0.98 (0.80, 1.21) | High |
| Gastrointestinal adverse events | 0.5 - 2.0 | 0.2 - 28.6 | Placebo | CHD | Andreis et.al, 2021^7^ | 10 | 2.00 (1.37, 2.93) | Moderate | 9 | 1.70 (1.20, 2.40) | High |
| Hospitalization | 0.5 - 1.0 | 1.0 - 12.0 | Placebo | CHD | Andreis et.al, 2022^4^ | 3 | 0.32 (0.12, 0.87) | High | 7 | 0.32 (0.12, 0.87) | High |
| MI | 0.5 - 1.0 | 1.0 - 36.0 | Placebo | CHD | Teo et.al, 2021^42^ | 6 | 0.75 (0.62, 0.91) | High | 4 | 0.72 (0.52, 1.00) | High |
| MI (acute) | 0.5 - 1.0 | 1.0 - 28.6 | Placebo | CHD | Shrestha, 2022^3^ | 3 | 0.80 (0.65,0.98) | High | 3 | 0.80 (0.65, 0.98) | High |
| Revascularization | 0.5 - 1.0 | 6.0 - 24.0 | No colchicine, placebo | CHD | Xia et.al, 2021^13^ | 5 | 0.61 (0.42, 0.89) | High | 4 | 0.61 (0.42, 0.89) | High |
| Revascularization | 0.5 - 1.0 | 7.1 - 28.6 | Placebo | CHD | Xu et.al, 2022^15^ | 5 | 0.61 (0.42, 0.89) | High | 4 | 0.61 (0.42, 0.89) | High |
| Revascularization | 0.5 - 2.0 | 0.2 - 28.6 | Placebo | CHD | Andreis et.al, 2022^4^ | 5 | 0.61 (0.42, 0.89) | High | 4 | 0.61 (0.42, 0.89) | High |
| Stroke | 0.5 - 1.0 | 6.0 - 24.0 | Placebo | CHD | Xia et.al, 2021^13^ | 5 | 0.47 (0.28, 0.81) | High | 5 | 0.47 (0.28, 0.81) | High |
| Stroke | 0.5 - 1.8 | 1.0 - 36.0 | Placebo | CHD | Kofler et.al, 2021^11^ | 7 | 0.50 (0.31, 0.81) | High | 7 | 0.50 (0.31, 0.81) | High |
| ACS, acute coronary syndrome; AIS, acute ischaemic stroke; CHD, coronary heart disease; CI, confidence interval; GRADE, Grading of Recommendations Assessment, Development, and Evaluation; m, month; MI, myocardial infarction; NA, not available; No., number; RCTs, randomised controlled trials. | | | | | | | | | | | |

| **Supplementary Table S6. Summary of sensitivity analysis excluding studies with small sample size (< 25^th^ percentile)** | | | | | | | | | |
| --- | --- | --- | --- | --- | --- | --- | --- | --- | --- |
| **Outcomes** | **Patients** | **Author, year,  reference** | **Metric** | **Primary analysis** | | | **Sensitivity analysis** | | |
|  |  |  |  | **No. of  RCTs** | **Random effect  size (95% CI)** | **GRADE** | **No. of RCTs** | **Random effect  size (95% CI)** | **GRADE** |
| **Adverse events** |  |  |  |  |  |  |  |  |  |
| Diarrhea | ACS, CCS | Ma et.al, 2022^5^ | RR | 5 | 3.26 (1.29, 8.25) | Moderate | 4 | 1.11 (1.00, 1.23) | Very low |
| Drug discontinuation | CHD | Papageorgiou et.al, 2017^8^ | OR | 5 | 7.59 (3.40, 16.93) | Moderate | 3 | 10.66 (3.05, 37.29) | High |
| Drug discontinuation | ACS, CCS | Grajek et.al, 2021^9^ | RR | 10 | 1.60 (1.06, 2.42) | Moderate | 8 | 0.75 (0.62, 0.91) | Moderate |
| Drug discontinuation | CHD | Andreis et.al, 2021^7^ | RR | 8 | 1.61 (1.11, 2.35) | High | 6 | 1.63 (1.07, 2.49) | Very low |
| Drug discontinuation (without PROBE studies) | ACS, CCS | Grajek et.al, 2021^9^ | RR | 8 | 1.34 (0.97, 1.84) | Moderate | 7 | 0.75 (0.62, 0.91) | Moderate |
| Gastrointestinal adverse events | ACS, CCS | Ma et.al, 2022^5^ | RR | 14 | 2.07 (1.45, 2.95) | Moderate | 11 | 1.11 (1.00, 1.23) | Very low |
| Gastrointestinal adverse events | ACS | Bao et.al, 2022^18^ | RR | 9 | 1.89 (1.25, 2.84) | Moderate | 7 | 1.35 (0.96, 1.88) | High |
| Gastrointestinal adverse events | ACS | Diaz-Arocutipa et.al, 2021^20^ | RR | 5 | 2.49 (0.48, 12.99) | Moderate | 4 | 1.35 (0.96, 1.88) | High |
| Gastrointestinal adverse events | CHD | Andreis et.al, 2021^7^ | RR | 10 | 2.00 (1.37, 2.93) | Moderate | 8 | 1.95 (1.30, 2.84) | Very low |
| Gastrointestinal adverse events | ACS | Zhou et.al, 2023^21^ | RR | 5 | 2.99 (1.14, 7.82) | Moderate | 4 | 2.89 (1.92, 4.35) | High |
| Gastrointestinal adverse events | CHD | Chen et.al, 2022^22^ | RR | 10 | 2.15 (1.40, 3.31) | High | 8 | 2.89 (1.92, 4.35) | High |
| Gastrointestinal adverse events | Surgery (aortic, CABG) | Agarwal et.al, 2023^12^ | RR | 6 | 2.20 (1.38, 3.51) | Moderate | 5 | 0.65 (0.48, 0.89) | High |
| Gastrointestinal adverse events | Surgery (aortic, CABG) | Agarwal et.al, 2023^12^ | RR | 5 | 1.64 (1.24, 2.19) | High | 4 | 0.45 (0.30, 0.66) | High |
| Postoperative adverse events | PCI | Wei et.al, 2023^23^ | RR | 5 | 1.55 (1.04, 2.32) | Moderate | 4 | 1.11 (1.00, 1.23) | Very low |
| **All-cause and cause-specific mortality** |  |  |  |  |  |  |  |  |  |
| All-cause mortality | ACS, AIS, surgery (PCI) | Masson et.al, 2020^26^ | OR | 7 | 0.85 (0.50, 1.42) | High | 6 | 0.62 (0.48, 0.80) | High |
| All-cause mortality | ACS, CCS | Ma et.al, 2022^5^ | RR | 13 | 1.00 (0.70, 1.42) | High | 10 | 1.09 (0.85, 1.39) | High |
| All-cause mortality | CHD | Kofler et.al, 2021^11^ | OR | 13 | 0.96 (0.65, 1.41) | High | 8 | 2.89 (1.92, 4.35) | High |
| All-cause mortality | Surgery (PCI) | Fu etal, 2021^31^ | OR | 4 | 0.89 (0.60, 1.32) | High | 3 | 0.94 (0.62, 1.43) | High |
| All-cause mortality | CHD | Liao et.al, 2021^32^ | OR | 5 | 0.93 (0.63, 1.36) | High | 4 | 0.98 (0.65, 1.47) | High |
| All-cause mortality | ACS | Younas et.al, 2024^19^ | RR | 8 | 1.00 (0.72, 1.39) | High | 7 | 2.89 (1.92, 4.35) | High |
| All-cause mortality | ACS | Diaz-Arocutipa et.al, 2021^20^ | RR | 5 | 1.06 (0.61, 1.85) | High | 4 | 1.35 (0.96, 1.88) | High |
| All-cause mortality | CHD | Andreis et.al, 2022^4^ | RR | 11 | 1.01 (0.71, 1.43) | High | 8 | 1.07 (0.83, 1.37) | High |
| All-cause mortality | CHD | Chen et.al, 2022^22^ | OR | 11 | 1.09 (0.85, 1.40) | High | 8 | 2.89 (1.92, 4.35) | High |
| All-cause mortality | CHD | Xiang et.al, 2021^6^ | RR | 8 | 0.70 (0.61, 0.80) | High | 6 | 2.89 (1.92, 4.35) | High |
| All-cause mortality | CHD | Bytyçi et.al, 2022^33^ | RR | 9 | 1.05 (0.71, 1.53) | High | 6 | 1.35 (0.96, 1.88) | High |
| CV mortality | CHD | Kofler et.al, 2021^11^ | OR | 9 | 0.82 (0.46, 0.90) | High | 6 | 2.89 (1.92, 4.35) | High |
| CV mortality | ACS | Ullah et.al, 2021^34^ | OR | 5 | 0.91 (0.53, 1.59) | High | 3 | 0.45 (0.30, 0.66) | High |
| CV mortality | ACS | Diaz-Arocutipa et.al, 2021^20^ | RR | 4 | 0.91 (0.52, 1.61) | High | 3 | 1.35 (0.96, 1.88) | High |
| CV mortality | ACS, CCS | Ma et.al, 2022^5^ | RR | 9 | 0.91 (0.49, 1.68) | Moderate | 8 | 0.92 (0.62, 1.36) | Low |
| CV mortality | ACS, AIS, surgery (PCI) | Masson et.al, 2020^26^ | OR | 6 | 0.42 (0.07, 2.61) | Moderate | 3 | 0.62 (0.48, 0.80) | High |
| CV mortality | ACS, CCS, surgery (PCI) | Xu et.al, 2022^15^ | RR | 6 | 0.74 (0.58, 0.95) | High | 5 | 2.89 (1.92, 4.35) | High |
| CV mortality | CHD | Chen et.al, 2022^22^ | OR | 9 | 0.80 (0.54, 1.18) | High | 7 | 2.89 (1.92, 4.35) | High |
| CV mortality | CHD | Andreis et.al, 2022^4^ | RR | 9 | 0.73 (0.45, 1.21) | High | 6 | 2.89 (1.92, 4.35) | High |
| CV mortality | CHD | Bytyçi et.al, 2022^33^ | RR | 7 | 0.75 (0.40, 1.43) | High | 6 | 1.35 (0.96, 1.88) | High |
| Non-CV mortality | CHD | Akl et.al, 2024^25^ | Peto OR | 8 | 1.54 (1.10, 2.15) | Moderate | 4 | 0.75 (0.62, 0.91) | Moderate |
| Non-CV mortality | ACS, CCS | Ma et.al, 2022^5^ | RR | 7 | 1.32 (0.91, 1.92) | High | 6 | 1.34 (0.97, 1.87) | High |
| Non-CV mortality | CHD | Chen et.al, 2022^22^ | OR | 9 | 1.38 (1.00, 1.92) | High | 7 | 2.89 (1.92, 4.35) | High |
| **Cardiovascular disorders** |  |  |  |  |  |  |  |  |  |
| ACS | ACS | Ullah et.al, 2021^34^ | OR | 6 | 0.80 (0.51, 1.24) | High | 4 | 0.45 (0.30, 0.66) | High |
| ACS | ACS, CCS | Abrantes et.al, 2021^2^ | RR | 7 | 0.62 (0.44, 0.89) | High | 5 | 0.72 (0.59, 0.86) | Moderate |
| ACS | CHD | Chen et.al, 2023^17^ | OR | 5 | 0.70 (0.53, 0.92) | High | 3 | 0.75 (0.62, 0.91) | Moderate |
| ACS | ACS, CCS | Akl et.al, 2024^25^ | OR | 5 | 0.72 (0.58, 0.89) | High | 3 | 0.62 (0.48, 0.80) | High |
| ACS (new) | CHD | Chen et.al, 2023^17^ | OR | 8 | 0.68 (0.57, 0.81) | High | 6 | 0.75 (0.62, 0.91) | Moderate |
| CHD | CHD | Andreis et.al, 2021^7^ | RR | 8 | 0.73 (0.64, 0.83) | High | 7 | 0.49 (0.30, 0.80) | High |
| MI | CHD | Sattar et.al, 2022^28^ | RR | 5 | 0.75 (0.62, 0.90) | High | 4 | 1.35 (0.96, 1.88) | High |
| MI | CHD | Akl et.al, 2024^25^ | OR | 6 | 0.75 (0.62, 0.91) | High | 5 | 0.75 (0.62, 0.91) | High |
| MI | CHD | Andreis et.al, 2022^4^ | RR | 7 | 0.76 (0.61, 0.96) | High | 6 | 2.89 (1.92, 4.35) | High |
| MI | ACS, CCS | Grajek et.al, 2021^9^ | RR | 7 | 0.73 (0.57, 0.95) | High | 6 | 0.75 (0.62, 0.91) | Moderate |
| MI | ACS, CCS | Ma et.al, 2022^5^ | RR | 9 | 0.60 (0.43, 0.83) | Moderate | 8 | 0.69 (0.59, 0.82) | Moderate |
| MI | CHD | Chen et.al, 2022^22^ | RR | 7 | 0.77 (0.64, 0.92) | High | 6 | 2.89 (1.92, 4.35) | High |
| MI | ACS | Zhou et.al, 2023^21^ | RR | 4 | 0.88 (0.67, 1.15) | High | 3 | 2.89 (1.92, 4.35) | High |
| MI (acute) | CHD | Xiang et.al, 2021^6^ | RR | 4 | 0.77 (0.64, 0.94) | High | 3 | 2.89 (1.92, 4.35) | High |
| MI (recurrent) | ACS | Diaz-Arocutipa et.al, 2021^20^ | RR | 4 | 0.87 (0.62, 1.22) | High | 3 | 1.35 (0.96, 1.88) | High |
| MI (recurrent) | CHD | Bytyçi et.al, 2022^33^ | RR | 7 | 0.78 (0.65, 0.93) | High | 6 | 1.35 (0.96, 1.88) | High |
| MI (recurrent) | ACS | Bao et.al, 2022^18^ | RR | 7 | 0.75 (0.49, 1.14) | High | 6 | 1.35 (0.96, 1.88) | High |
| MI (recurrent) | ACS | Younas et.al, 2024^19^ | RR | 6 | 0.78 (0.57, 1.06) | High | 5 | 2.89 (1.92, 4.35) | High |
| POAF | Surgery (CABG) | Kirov et.al, 2024^40^ | RR | 5 | 0.54 (0.40, 0.73) | High | 3 | 2.89 (1.92, 4.35) | High |
| POAF | Surgery (aortic, CABG, MR) | Zhao et.al, 2022^41^ | RR | 5 | 0.40 (0.25, 0.65) | High | 3 | 0.65 (0.47, 0.90) | High |
| POAF | CHD | Teo et.al, 2021^42^ | RR | 4 | 0.64 (0.48, 0.86) | High | 3 | 2.89 (1.92, 4.35) | High |
| POAF | Surgery (aortic, CABG) | Agarwal et.al, 2023^12^ | RR | 8 | 0.70 (0.59, 0.82) | High | 6 | 0.69 (0.58, 0.82) | High |
| POAF (< 1 m) | Surgery (aortic, CABG, MR) | Zhao et.al, 2022^41^ | RR | 5 | 0.65 (0.49, 0.86) | High | 3 | 0.75 (0.62, 0.91) | Moderate |
| POAF (< 1 m) | Surgery (aortic, CABG, MR) | Agarwal et.al, 2023^12^ | RR | 5 | 0.63 (0.49, 0.81) | High | 4 | 0.64 (0.49, 0.83) | High |
| POAF (no history of AF) | Surgery (aortic, CABG) | Agarwal et.al, 2023^12^ | RR | 4 | 0.68 (0.51, 0.90) | High | 3 | 0.69 (0.52, 0.93) | High |
| **Hospitalization** |  |  |  |  |  |  |  |  |  |
| Rehospitalization | CHD | Shrestha, 2022^3^ | OR | 4 | 0.64 (0.20, 2.06) | Moderate | 3 | 0.10 (0.58, 1.72) | Moderate |
| **MACEs** |  |  |  |  |  |  |  |  |  |
| ACS, AF, mortality, revascularization and stroke | Surgery (CABG, PCI) | Chen et.al, 2023^17^ | OR | 7 | 0.68 (0.44, 1.05) | Moderate | 4 | 0.75 (0.62, 0.91) | Moderate |
| ACS, CHD, post-angioplasty and stroke | CHD | Verma et.al, 2015^10^ | RR | 4 | 0.40 (0.25, 0.65) | High | 3 | 0.39 (0.23, 0.65) | High |
| Adverse cardiovascular events a | ACS | Younas et.al, 2024^19^ | RR | 7 | 0.75 (0.60, 0.94) | High | 5 | 2.89 (1.92, 4.35) | High |
| All-cause mortality, cardiac arrest, ISR, MI, stent thrombosis and stroke | Surgery (PCI) | Wei et.al, 2023^23^ | RR | 5 | 0.70 (0.58, 0.84) | High | 4 | 1.11 (1.00, 1.23) | Very low |
| All-cause mortality, CV mortality, MI and stroke | ACS, CCS | Abrantes et.al, 2021^2^ | RR | 6 | 0.65 (0.49, 0.86) | High | 4 | 1.35 (0.96, 1.88) | High |
| All-cause mortality, CV mortality, recurrent MI and stroke | CHD | Bytyçi et.al, 2022^33^ | RR | 7 | 0.67 (0.55, 0.83) | High | 6 | 1.35 (0.96, 1.88) | High |
| All-cause mortality, CV mortality and stroke | CHD | Aw et.al, 2022^24^ | RR | 7 | 0.73 (0.61, 0.87) | Moderate | 6 | 2.89 (1.92, 4.35) | High |
| All-cause mortality, HF, MI, revascularization and stroke (≤ 3 d) | ACS | Zhou et.al, 2023^21^ | RR | 5 | 0.58 (0.44, 0.78) | High | 4 | 2.89 (1.92, 4.35) | High |
| CV mortality, coronary revascularization, MI and stroke | ACS, CCS, surgery (PCI) | Xu et.al, 2022^15^ | RR | 7 | 0.64 (0.51, 0.80) | High | 6 | 2.89 (1.92, 4.35) | High |
| CV mortality, MI and stroke | ACS, CCS | Ma et.al, 2022^5^ | RR | 7 | 0.54 (0.38, 0.77) | Moderate | 6 | 0.66 (0.57, 0.77) | Low |
| CV mortality, MI and stroke | ACS, CCS | Akl et.al, 2024^25^ | OR | 6 | 0.70 (0.60, 0.83) | Moderate | 5 | 0.73 (0.51, 0.90) | High |
| Major adverse CV and cerebrovascular events | CHD | Andreis et.al, 2022^4^ | RR | 11 | 0.67 (0.56, 0.80) | High | 8 | 2.89 (1.92, 4.35) | High |
| **Revascularization** |  |  |  |  |  |  |  |  |  |
| Revascularization | CHD | Chen et.al, 2023^17^ | OR | 7 | 0.65 (0.53, 0.78) | High | 5 | 0.75 (0.62, 0.91) | Moderate |
| Repeat vessel revascularization | CHD | Aw et.al, 2022^24^ | RR | 4 | 0.47 (0.31, 0.72) | Moderate | 3 | 2.89 (1.92, 4.35) | High |
| **Stroke** |  |  |  |  |  |  |  |  |  |
| Ischaemic stroke | CHD | Xiang et.al, 2021^6^ | RR | 5 | 0.49 (0.30, 0.79) | High | 4 | 2.89 (1.92, 4.35) | High |
| Stroke | ACS, CCS | Ma et.al, 2022^5^ | RR | 6 | 0.50 (0.31, 0.80) | High | 5 | 0.50 (0.31, 0.82) | High |
| Stroke | ACS, CCS | Abrantes et.al, 2021^2^ | RR | 6 | 0.48 (0.30, 0.78) | High | 5 | 0.75 (0.62, 0.91) | Moderate |
| Stroke | CHD | Katsanos et.al, 2020^48^ | RR | 4 | 0.31 (0.13, 0.71) | High | 3 | 2.89 (1.92, 4.35) | High |
| Stroke | ACS, CHD | Akl et.al, 2024^25^ | OR | 8 | 0.47 (0.30, 0.74) | High | 5 | 0.62 (0.48, 0.80) | High |
| Stroke | CHD | Aw et.al, 2022^24^ | RR | 7 | 0.50 (0.31, 0.81) | Moderate | 6 | 2.89 (1.92, 4.35) | High |
| Stroke | CHD | Chen et.al, 2023^17^ | OR | 7 | 0.51 (0.32, 0.82) | High | 6 | 0.75 (0.62, 0.91) | Moderate |
| Stroke | ACS | Bao et.al, 2022^18^ | RR | 5 | 0.39 (0.18, 0.81) | High | 4 | 1.35 (0.96, 1.88) | High |
| Stroke | CHD | Andreis et.al, 2022^4^ | RR | 7 | 0.48 (0.30, 0.77) | High | 6 | 2.89 (1.92, 4.35) | High |
| Stroke | CHD | Bytyçi et.al, 2022^33^ | RR | 7 | 0.47 (0.29, 0.76) | High | 6 | 1.35 (0.96, 1.88) | High |
| ACS, acute coronary syndrome; AF, atrial fibrillation; AIS, acute ischaemic stroke; CABG, coronary artery bypass grafting; CCS, chronic coronary syndromes; CHD, coronary heart disease; CI, confidence interval; CV, cardiovascular; d, day; GRADE, Grading of Recommendations Assessment, Development, and Evaluation; HF, heart failure; ISR, in-stent restenosis; MI, myocardial infarction; MR, myocardial revascularization; No., number; OR, odds ratio; PCI, percutaneous coronary intervention; POAF, postoperative atrial fibrillation; PROBE, Prospective, Randomized, Open-label, Blinded Endpoint; RCTs, randomised controlled trials; RR, risk ratio. | | | | | | | | | |
| ^a^ Adverse cardiovascular events: ACS, CV mortality, HF, MI, resuscitated cardiac arrest, stroke, UA, urgent hospitalization for angina and ventricular arrhythmias. | | | | | | | | | |

**Supplementary Table S7. Summary of sensitivity analysis excluding studies with high risk of bias**

| **Outcomes** | **Patients** | **Author, year,  reference** | **Metric** | **Primary analysis** | | | **Sensitivity analysis** | | |
| --- | --- | --- | --- | --- | --- | --- | --- | --- | --- |
|  |  |  |  | **No. of  RCTs** | **Random effect  size (95% CI)** | **GRADE** | **No. of  RCTs** | **Random effect  size (95% CI)** | **GRADE** |
| **Adverse events** |  |  |  |  |  |  |  |  |  |
| Adverse events | ACS | Abrantes et.al, 2021^2^ | RR | 6 | 1.10 (0.90, 1.35) | Moderate | 5 | 1.02 (0.86, 1.22) | Moderate |
| Diarrhea | ACS, CCS | Ma et.al, 2022^5^ | RR | 5 | 3.26 (1.29, 8.25) | Moderate | 4 | 4.04 (1.09, 15.01) | Very low |
| Drug discontinuation | ACS, CCS | Grajek et.al, 2021^9^ | RR | 10 | 1.60 (1.06, 2.42) | Moderate | 8 | 1.33 (0.97, 1.84) | Moderate |
| Drug discontinuation | CHD | Andreis et.al, 2021^7^ | RR | 8 | 1.61 (1.11, 2.35) | High | 7 | 1.56 (1.07, 2.27) | Moderate |
| Gastrointestinal adverse events | ACS, CCS | Ma et.al, 2022^5^ | RR | 14 | 2.07 (1.45, 2.95) | Moderate | 12 | 1.94 (1.35, 2.77) | Very low |
| Gastrointestinal adverse events | ACS | Bao et.al, 2022^18^ | RR | 9 | 1.89 (1.25, 2.84) | Moderate | 7 | 1.71 (1.12, 2.60) | Low |
| Gastrointestinal adverse events | CHD | Andreis et.al, 2021^7^ | RR | 10 | 2.00 (1.37, 2.93) | Moderate | 8 | 1.48 (1.08, 2.04) | Low |
| Gastrointestinal adverse events | ACS | Zhou et.al, 2023^21^ | RR | 5 | 2.99 (1.14, 7.82) | Moderate | 4 | 2.24 (0.70, 7.18) | Low |
| Gastrointestinal adverse events | CHD | Chen et.al, 2022^22^ | RR | 10 | 2.15 (1.40, 3.31) | High | 8 | 1.80 (1.24, 2.63) | Very low |
| Postoperative adverse events | PCI | Wei et.al, 2023^23^ | RR | 5 | 1.55 (1.04, 2.32) | Moderate | 4 | 1.45 (1.00, 2.09) | Low |
| **All-cause and cause-specific mortality** |  |  |  |  |  |  |  |  |  |
| All-cause mortality | ACS, AIS, surgery (PCI) | Masson et.al, 2020^26^ | OR | 7 | 0.85 (0.50, 1.42) | High | 5 | 0.99 (0.68, 1.46) | High |
| All-cause mortality | CHD | Sattar et.al, 2022^28^ | RR | 5 | 1.14 (0.76, 1.69) | High | 4 | 1.17 (0.81, 1.69) | High |
| All-cause mortality | CHD | Sen et.al, 2021^14^ | RR | 4 | 1.04 (0.61, 1.78) | Moderate | 3 | 1.20 (0.77, 1.88) | Moderate |
| All-cause mortality | ACS, CCS | Grajek et.al, 2021^9^ | RR | 8 | 0.99 (0.68, 1.45) | High | 7 | 1.12 (0.87, 1.43) | High |
| All-cause mortality | ACS, CCS | Ma et.al, 2022^5^ | RR | 13 | 1.00 (0.70, 1.42) | High | 10 | 1.00 (0.71, 1.42) | High |
| All-cause mortality | CHD | Kofler et.al, 2021^11^ | OR | 13 | 0.96 (0.65, 1.41) | High | 10 | 1.13 (0.87, 1.47) | High |
| All-cause mortality | Surgery (PCI) | Fu etal, 2021^31^ | OR | 4 | 0.89 (0.60, 1.32) | High | 3 | 0.94 (0.62, 1.43) | High |
| All-cause mortality | CHD | Liao et.al, 2021^32^ | OR | 5 | 0.93 (0.63, 1.36) | High | 4 | 0.98 (0.65, 1.47) | Moderate |
| All-cause mortality | CHD | Andreis et.al, 2022^4^ | RR | 11 | 1.01 (0.71, 1.43) | High | 10 | 0.10 (0.67, 1.49) | High |
| All-cause mortality | CHD | Chen et.al, 2022^22^ | OR | 11 | 1.09 (0.85, 1.40) | High | 9 | 1.13 (0.87, 1.47) | High |
| All-cause mortality | CHD | Xiang et.al, 2021^6^ | RR | 8 | 0.70 (0.61, 0.80) | High | 4 | 0.73 (0.64, 0.85) | High |
| All-cause mortality | CHD | Bytyçi et.al, 2022^33^ | RR | 9 | 1.05 (0.71, 1.53) | High | 8 | 1.04 (0.67, 1.61) | High |
| All-cause mortality (follow-up time ≥ 6 m) | CHD | Chen et.al, 2023^17^ | OR | 6 | 1.09 (0.85, 1.40) | High | 4 | 1.18 (0.81, 1.71) | High |
| CV mortality | CHD | Kofler et.al, 2021^11^ | OR | 9 | 0.82 (0.46, 0.90) | High | 7 | 0.86 (0.57, 1.29) | High |
| CV mortality | ACS, CCS | Ma et.al, 2022^5^ | RR | 9 | 0.91 (0.49, 1.68) | Moderate | 6 | 0.92 (0.62, 1.36) | Moderate |
| CV mortality | ACS, CCS | Grajek et.al, 2021^9^ | RR | 6 | 0.80 (0.55, 1.18) | High | 5 | 0.84 (0.57, 1.23) | High |
| CV mortality | ACS, CCS | Abrantes et.al, 2021^2^ | RR | 6 | 0.79 (0.53, 1.18) | High | 4 | 0.83 (0.55, 1.24) | High |
| CV mortality | CHD | Xia et.al, 2021^13^ | RR | 5 | 0.79 (0.43, 1.45) | High | 4 | 0.86 (0.57, 1.29) | High |
| CV mortality | ACS, CCS | Grajek et.al, 2021^9^ | RR | 5 | 0.80 (0.53, 1.22) | High | 4 | 0.84 (0.56, 1.27) | High |
| CV mortality | CHD | Sen et.al, 2021^14^ | RR | 4 | 0.71 (0.48, 1.05) | High | 3 | 0.86 (0.57, 1.29) | High |
| CV mortality | CHD | Chen et.al, 2023^17^ | OR | 4 | 0.77 (0.52, 1.15) | High | 3 | 0.85 (0.56, 1.29) | Moderate |
| CV mortality | CHD | Chen et.al, 2022^22^ | OR | 9 | 0.80 (0.54, 1.18) | High | 7 | 0.88 (0.58, 1.31) | High |
| CV mortality | CHD | Andreis et.al, 2022^4^ | RR | 9 | 0.73 (0.45, 1.21) | High | 7 | 0.84 (0.56, 1.25) | High |
| CV mortality | CHD | Bytyçi et.al, 2022^33^ | RR | 7 | 0.75 (0.40, 1.43) | High | 5 | 0.84 (0.56, 1.27) | High |
| Non-CV mortality | ACS, CCS | Ma et.al, 2022^5^ | RR | 7 | 1.32 (0.91, 1.92) | High | 6 | 1.33 (0.93, 1.90) | High |
| Non-CV mortality | CHD | Sen et.al, 2021^14^ | RR | 4 | 1.53 (1.10, 2.14) | High | 3 | 1.43 (0.94, 2.17) | High |
| Non-CV mortality | CHD | Xia et.al, 2021^13^ | RR | 4 | 1.50 (0.93, 2.40) | High | 3 | 1.43 (0.94, 2.17 ) | High |
| Non-CV mortality | CHD | Chen et.al, 2022^22^ | OR | 9 | 1.38 (1.00, 1.92) | High | 7 | 1.40 (0.95, 2.07) | High |
| **Cardiovascular disorders** |  |  |  |  |  |  |  |  |  |
| ACS | ACS | Ullah et.al, 2021^34^ | OR | 6 | 0.80 (0.51, 1.24) | High | 4 | 0.78 (0.49, 1.26) | High |
| ACS | ACS, CCS | Abrantes et.al, 2021^2^ | RR | 7 | 0.62 (0.44, 0.89) | High | 4 | 0.76 (0.59, 0.97) | High |
| ACS | CHD | Chen et.al, 2023^17^ | OR | 5 | 0.70 (0.53, 0.92) | High | 4 | 0.68 (0.51, 0.92) | High |
| ACS | ACS, CCS | Akl et.al, 2024^25^ | OR | 5 | 0.72 (0.58, 0.89) | High | 3 | 0.75 (0.60, 0.95) | High |
| ACS (new) | CHD | Chen et.al, 2023^17^ | OR | 8 | 0.68 (0.57, 0.81) | High | 6 | 0.72 (0.60, 0.87) | High |
| CHD | CHD | Andreis et.al, 2021^7^ | RR | 8 | 0.73 (0.64, 0.83) | High | 7 | 0.72 (0.63, 0.82) | High |
| MI | CHD | Sattar et.al, 2022^28^ | RR | 5 | 0.75 (0.62, 0.90) | High | 3 | 0.76 (0.59, 0.98) | High |
| MI | CHD | Akl et.al, 2024^25^ | OR | 6 | 0.75 (0.62, 0.91) | High | 3 | 0.79 (0.58, 1.06) | High |
| MI | CHD | Xia et.al, 2021^13^ | RR | 4 | 0.73 (0.55, 0.98) | High | 3 | 0.80 (0.66, 0.96) | High |
| MI | ACS, CCS | Grajek et.al, 2021^9^ | RR | 4 | 0.72 (0.52, 1.00) | High | 3 | 0.80 (0.66, 0.96) | Moderate |
| MI | CHD | Andreis et.al, 2022^4^ | RR | 7 | 0.76 (0.61, 0.96) | High | 6 | 0.76 (0.59, 0.97) | High |
| MI | ACS, CCS | Grajek et.al, 2021^9^ | RR | 7 | 0.73 (0.57, 0.95) | High | 5 | 0.79 (0.65, 0.96) | High |
| MI | ACS, CCS | Ma et.al, 2022^5^ | RR | 9 | 0.60 (0.43, 0.83) | Moderate | 8 | 0.59 (0.42, 0.82) | Moderate |
| MI | CHD | Chen et.al, 2022^22^ | RR | 7 | 0.77 (0.64, 0.92) | High | 5 | 0.80 (0.67, 0.96) | High |
| MI | ACS | Zhou et.al, 2023^21^ | RR | 4 | 0.88 (0.67, 1.15) | High | 3 | 1.29 (0.30, 5.63) | Moderate |
| MI (recurrent) | CHD | Bytyçi et.al, 2022^33^ | RR | 7 | 0.78 (0.65, 0.93) | High | 5 | 0.80 (0.67, 0.96) | High |
| MI (recurrent) | ACS | Bao et.al, 2022^18^ | RR | 7 | 0.75 (0.49, 1.14) | High | 6 | 0.75 (0.48, 1.17) | High |
| POAF | Surgery (CABG) | Kirov et.al, 2024^40^ | RR | 5 | 0.54 (0.40, 0.73) | High | 4 | 0.49 (0.34, 0.69) | High |
| POAF | Surgery (aortic, CABG, MR) | Zhao et.al, 2022^41^ | RR | 5 | 0.40 (0.25, 0.65) | High | 4 | 0.66 (0.49, 0.89) | High |
| POAF | CHD | Teo et.al, 2021^42^ | RR | 4 | 0.64 (0.48, 0.86) | High | 3 | 0.65 (0.47, 0.90) | High |
| POAF | Surgery (aortic, CABG) | Agarwal et.al, 2023^12^ | RR | 8 | 0.70 (0.59, 0.82) | High | 7 | 0.73 (0.61, 0.87) | High |
| POAF (< 1 m) | Surgery (aortic, CABG, MR) | Zhao et.al, 2022^41^ | RR | 5 | 0.65 (0.49, 0.86) | High | 4 | 0.66 (0.49, 0.89) | High |
| POAF (< 1 m) | Surgery (aortic, CABG, MR) | Agarwal et.al, 2023^12^ | RR | 5 | 0.63 (0.49, 0.81) | High | 4 | 0.68 (0.51, 0.91) | High |
| **MACEs** |  |  |  |  |  |  |  |  |  |
| ACS, AF, mortality, revascularization and stroke | Surgery (CABG, PCI) | Chen et.al, 2023^17^ | OR | 7 | 0.68 (0.44, 1.05) | Moderate | 3 | 0.88 (0.52, 1.49) | Moderate |
| All-cause mortality, cardiac arrest, ISR, MI, stent thrombosis and stroke | Surgery (PCI) | Wei et.al, 2023^23^ | RR | 5 | 0.70 (0.58, 0.84) | High | 4 | 0.66 (0.51, 0.85) | High |
| All-cause mortality, CV mortality, MI and stroke | ACS, CCS | Abrantes et.al, 2021^2^ | RR | 6 | 0.65 (0.49, 0.86) | High | 3 | 0.76 (0.65, 0.90) | High |
| All-cause mortality, CV mortality, MI and stroke | CHD | Xia et.al, 2021^13^ | RR | 5 | 0.65 (0.52, 0.82) | High | 4 | 0.72 (0.63, 0.83) | Moderate |
| All-cause mortality, CV mortality, recurrent MI and stroke | CHD | Bytyçi et.al, 2022^33^ | RR | 7 | 0.67 (0.55, 0.83) | High | 5 | 0.73 (0.64, 0.83) | High |
| All-cause mortality, ACS, CV mortality, revascularization and stroke | CHD | Shrestha, 2022^1^ | OR | 4 | 0.63 (0.48, 0.83) | Moderate | 3 | 0.71 (0.62, 0.82) | High |
| All-cause mortality, HF, MI, revascularization and stroke (≤ 3 d) | ACS | Zhou et.al, 2023^21^ | RR | 5 | 0.58 (0.44, 0.78) | High | 4 | 0.60 (0.45, 0.60) | Moderate |
| CV mortality, MI and stroke | CHD | Akl et.al, 2024^25^ | Peto OR | 5 | 0.67 (0.58, 0.77) | Moderate | 3 | 0.71 (0.62, 0.83) | Moderate |
| CV mortality, MI and stroke | ACS, CCS | Ma et.al, 2022^5^ | RR | 7 | 0.54 (0.38, 0.77) | Moderate | 6 | 0.53 (0.37, 0.76) | Moderate |
| CV mortality, MI and stroke | ACS, CCS | Akl et.al, 2024^25^ | OR | 6 | 0.70 (0.60, 0.83) | Moderate | 3 | 0.75 (0.62, 0.89) | High |
| CV mortality, MI and stroke | Atherosclerosis | Fiolet et.al, 2021^45^ | RR | 4 | 0.75 (0.61, 0.92) | High | 3 | 0.79 (0.67, 0.93) | High |
| CV mortality, MI,PCI, revascularization and stroke | ACS, CCS | Grajek et.al, 2021^9^ | RR | 5 | 0.70 (0.55, 0.88) | Moderate | 4 | 0.78 (0.67, 0.91) | High |
| Major adverse CV and cerebrovascular events | CHD | Andreis et.al, 2022^4^ | RR | 11 | 0.67 (0.56, 0.80) | High | 8 | 0.72 (0.64, 0.82) | High |
| **Revascularization** |  |  |  |  |  |  |  |  |  |
| Revascularization | ACS, CCS | Grajek et.al, 2021^9^ | RR | 6 | 0.57 (0.41, 0.80) | High | 5 | 0.61 (0.43, 0.87) | High |
| Revascularization | Atherosclerosis | Fiolet et.al, 2021^45^ | RR | 5 | 0.77 (0.66, 0.90) | High | 4 | 0.79 (0.68, 0.91) | High |
| Revascularization | CHD | Chen et.al, 2023^17^ | OR | 7 | 0.65 (0.53, 0.78) | High | 4 | 0.61 (0.42, 0.88) | High |
| Revascularization | ACS, CCS | Abrantes et.al, 2021^2^ | RR | 4 | 0.61 (0.42, 0.89) | High | 3 | 0.58 (0.37, 0.92) | Moderate |
| Revascularization | ACS, CCS | Grajek et.al, 2021^9^ | RR | 5 | 0.57 (0.40, 0.82) | High | 4 | 0.61 (0.42, 0.89) | High |
| **Stroke** |  |  |  |  |  |  |  |  |  |
| Ischaemic stroke | CHD | Xiang et.al, 2021^6^ | RR | 5 | 0.49 (0.30, 0.79) | High | 3 | 0.51 (0.21, 1.23) | High |
| Ischaemic stroke | CHD | Chen et.al, 2022^22^ | RR | 5 | 0.47 (0.30, 0.76) | High | 4 | 0.48 (0.25, 0.92) | High |
| Non-cardio-embolic ischaemic stroke | CHD | Shrestha, 2022^3^ | OR | 6 | 0.48 (0.30, 0.76) | High | 5 | 0.51 (0.31, 0.83) | High |
| Stroke | ACS, CCS | Ma et.al, 2022^5^ | RR | 6 | 0.50 (0.31, 0.80) | High | 5 | 0.50 (0.30, 0.83) | High |
| Stroke | ACS, CCS | Abrantes et.al, 2021^2^ | RR | 6 | 0.48 (0.30, 0.78) | High | 3 | 0.45 (0.24, 0.84) | High |
| Stroke | CHD | Katsanos et.al, 2020^48^ | RR | 4 | 0.31 (0.13, 0.71) | High | 3 | 0.32 (0.13, 0.79) | High |
| Stroke | ACS, CHD | Akl et.al, 2024^25^ | OR | 8 | 0.47 (0.30, 0.74) | High | 4 | 0.48 (0.23, 1.01) | Moderate |
| Stroke | CHD | Sattar et.al, 2022^28^ | RR | 4 | 0.46 (0.28, 0.74) | High | 3 | 0.47 (0.25, 0.87) | High |
| Stroke | ACS, CCS | Grajek et.al, 2021^9^ | RR | 5 | 0.47 (0.28, 0.81) | High | 4 | 0.48 (0.25, 0.92) | High |
| Stroke | CHD | Chen et.al, 2023^17^ | OR | 7 | 0.51 (0.32, 0.82) | High | 6 | 0.55 (0.32, 0.95) | High |
| Stroke | ACS | Bao et.al, 2022^18^ | RR | 5 | 0.39 (0.18, 0.81) | High | 4 | 0.43 (0.17, 1.05) | High |
| ACS, acute coronary syndrome; AF, atrial fibrillation; AIS, acute ischaemic stroke; CABG, coronary artery bypass grafting; CCS, chronic coronary syndromes; CHD, coronary heart disease; CI, confidence interval; CV, cardiovascular; d, day; GRADE, Grading of Recommendations Assessment, Development, and Evaluation; HF, heart failure; ISR, in-stent restenosis; m, month; MI, myocardial infarction; MR, myocardial revascularization; No., number; OR, odds ratio; PCI, percutaneous coronary intervention; POAF, postoperative atrial fibrillation; RCTs, randomised controlled trials; RR, risk ratio. | | | | | | | | | |

**Supplementary Table S8. The summary results of meta-analyses excluded due to lack of data for quantitative synthesis**

| **Author, year,**  **reference** | **No. of RCTs** | **Outcomes** | **Patients** | **Results** | **Conclusions** |
| --- | --- | --- | --- | --- | --- |
| Boczar et.al, 2022 ^49^ | NA | Cardiovascular outcomes | CHD | Decreased risk | Colchicine is associated with lower risks of cardiovascular outcomes |
| Fiolet et.al, 2019 ^50^ | 9 | Stable coronary artery disease | CHD | Decreased risk | Colchicine is associated with lower risks of stable coronary artery disease |
| Kundu et.al, 2024 ^51^ | 1 | Hemorrhagic stroke | Hemorrhagic stroke | Decreased risk | Colchicine is associated with lower risks of hemorrhagic stroke |
| Madanchi et.al, 2024 ^52^ | 14 | Acute and chronic coronary artery disease | CHD | Decreased risk | Colchicine is associated with lower risks of acute and chronic coronary artery disease |
| McKnight et.al, 2021 ^53^ | 9 | ACS | ACS | Decreased risk | Colchicine is associated with lower risks of ACS |
| Schattner et.al, 2022 ^54^ | 8 | Cardiovascular outcomes | ACS, MI, stable coronary disease, stroke | Decreased risk | Colchicine is associated with lower risks of cardiovascular outcomes |

ACS, acute coronary syndrome; AF, atrial fibrillation; CHD, coronary heart disease; MI, myocardial infarction; NA, not available; RCTs, randomised controlled trials.

**Su****pplementary Table S9. Subgroup analyses according to the dose of colchicine use on assessed associations**

| **Outcomes** | **Patients** | **Author, year,  reference** | **Lower dose of colchicine (≤ 0.5mg/d)** | | |  | **Higher dose of colchicine (> 0.5mg/d)** | | |
| --- | --- | --- | --- | --- | --- | --- | --- | --- | --- |
|  |  |  | **No. of RCTs** | **Random effect size (95% CI)** | **GRADE** |  | **No. of RCTs** | **Random effect size (95% CI)** | **GRADE** |
| **Adverse events** |  |  |  |  |  |  |  |  |  |
| Adverse events | ACS | Diaz-Arocutipa et.al, 2021^20^ | 3 | 0.99 (0.90, 1.08) | High |  | 3 | 1.73 (0.77, 3.85) | Very low |
| Diarrhea | ACS, CCS | Ma et.al, 2022^5^ | - | - | - |  | 3 | 4.44 (1.56, 12.61) | Low |
| Diarrhea | CHD | Xiang et.al, 2021^6^ | 3 | 2.10 (0.81, 5.40) | Very low |  | - | - | - |
| Drug discontinuation | ACS, CCS | Grajek et.al, 2021^9^ | 4 | 1.07 (0.67, 1.70) | Low |  | 6 | 2.02 (1.45, 2.80) | Moderate |
| Drug discontinuation | CHD | Kofler et.al, 2021^11^ | 4 | 1.04 (0.73, 1.49) | Low |  | 7 | 2.34 (1.63, 3.36) | High |
| Drug discontinuation | CHD | Andreis et.al, 2021^7^ | - | - | - |  | 6 | 2.07 (1.50, 2.86) | High |
| Drug discontinuation (without PROBE studies) | ACS, CCS | Grajek et.al, 2021^9^ | 3 | 0.96 (0.86, 1.08) | High |  | 5 | 1.99 (1.43, 2.77) | High |
| Gastrointestinal adverse events | CHD | Shrestha, 2022^3^ | 4 | 1.02 (0.79, 1.31) | High |  | 5 | 3.31 (1.26, 8.70) | Low |
| Gastrointestinal adverse events | ACS, CCS | Abrantes et.al, 2021^2^ | 6 | 1.75 (1.00, 3.07) | Low |  | 3 | 1.95 (0.93, 4.10) | Moderate |
| Gastrointestinal adverse events | ACS, CCS, surgery (PCI) | Xu et.al, 2022^15^ | 3 | 1.01 (0.88, 1.16) | High |  | - | - | - |
| Gastrointestinal adverse events | ACS, CCS | Ma et.al, 2022^5^ | 7 | 1.30 (0.94, 1.81) | Low |  | 7 | 2.83 (1.95, 4.11) | Moderate |
| Gastrointestinal adverse events | CHD | Kofler et.al, 2021^11^ | 4 | 1.32 (0.79, 2.21) | Moderate |  | 8 | 3.14 (1.66, 5.45) | Low |
| Gastrointestinal adverse events | CHD | Tien et.al, 2021^16^ | 3 | 2.59 (0.65, 10.32) | Low |  | 5 | 4.88 (2.34, 10.15) | Moderate |
| Gastrointestinal adverse events | CHD | Chen et.al, 2023^17^ | 5 | 1.32 (0.81, 2.14) | Moderate |  | 7 | 2.96 (1.54, 5.68) | Low |
| Gastrointestinal adverse events | ACS | Bao et.al, 2022^18^ | 3 | 1.74 (0.74, 4.10) | Moderate |  | 6 | 2.62 (1.24, 5.53) | Low |
| Gastrointestinal adverse events | ACS | Younas et.al, 2024^19^ | 4 | 3.32 (1.99, 5.55) | High |  | 3 | 0.99 (0.78, 1.26) | High |
| Gastrointestinal adverse events | ACS | Diaz-Arocutipa et.al, 2021^20^ | - | - | - |  | 3 | 5.62 (0.57, 55.38) | Low |
| Gastrointestinal adverse events | CHD | Andreis et.al, 2021^7^ | - | - | - |  | 8 | 2.61 ( 1.53, 4.43) | Moderate |
| Gastrointestinal adverse events | ACS | Zhou et.al, 2023^21^ | - | - | - |  | 3 | 6.74 (1.87, 24.27) | High |
| Gastrointestinal adverse events | CHD | Chen et.al, 2022^22^ | 3 | 2.49 (0.66, 9.36) | Low |  | 7 | 2.48 (1.40, 4.39) | Low |
| Postoperative adverse events | PCI | Wei et.al, 2023^23^ | - | - | - |  | 3 | 2.41 (0.72, 8.09) | Low |
| **All-cause and cause-specific mortality** |  |  |  |  |  |  |  |  |  |
| All-cause mortality | CHD | Sattar et.al, 2022^28^ | - | - | - |  | 3 | 1.41 (0.17, 11.62) | Moderate |
| All-cause mortality | CHD, stroke | Fiolet et.al, 2024^29^ | 5 | 1.07 (0.88, 1.31) | High |  | - | - | - |
| All-cause mortality | CHD | Sen et.al, 2021^14^ | 3 | 0.97 (0.63, 1.49) | Moderate |  | - | - | - |
| All-cause mortality | ACS, CCS, surgery (PCI) | Aw et.al, 2022^24^ | - | - | - |  | 4 | 1.33 (0.28, 6.30) | High |
| All-cause mortality | ACS, CCS | Ma et.al, 2022^5^ | 6 | 1.10 (0.67, 1.80) | Moderate |  | 7 | 0.60 (0.25, 1.45) | High |
| All-cause mortality | Surgery (PCI) | Fu etal, 2021^31^ | - | - | - |  | 3 | 0.50 (0.18, 1.43) | High |
| All-cause mortality | CHD | Liao et.al, 2021^32^ | - | - | - |  | 4 | 0.69 (0.27, 1.78) | High |
| All-cause mortality | ACS | Bao et.al, 2022^18^ | - | - | - |  | 3 | 2.50 (0.56, 11.12) | Moderate |
| All-cause mortality | ACS | Younas et.al, 2024^19^ | 6 | 1.45 (0.52, 4.06) | High |  | - | - | - |
| All-cause mortality | ACS | Diaz-Arocutipa et.al, 2021^20^ | - | - | - |  | 3 | 3.05 (0.66, 14.07) | High |
| All-cause mortality | CHD | Andreis et.al, 2022^4^ | 6 | 1.02 (0.62, 1.68) | Moderate |  | 5 | 0.64 (0.17, 2.45) | Moderate |
| All-cause mortality | CHD | Xiang et.al, 2021^6^ | 4 | 0.66 (0.51, 0.86) | High |  | 4 | 0.68 (0.18, 2.57) | Moderate |
| All-cause mortality | CHD | Bytyçi et.al, 2022^33^ | 5 | 1.04 (0.61, 1.78) | Moderate |  | 4 | 0.94 (0.19, 4.57) | High |
| All-cause mortality (follow-up time ≥ 6 m) | CHD | Chen et.al, 2023^17^ | 3 | 0.96 (0.62, 1.50) | Moderate |  | 3 | 1.39 (0.16, 11.80) | Moderate |
| CV mortality | CHD | Kofler et.al, 2021^11^ | 4 | 0.82 (0.54, 1.25) | Moderate |  | 5 | 1.57 (0.36, 6.96) | Moderate |
| CV mortality | CHD | Akl et.al, 2024^25^ | 3 | 0.65 (0.33, 1.28) | Low |  | - | - | - |
| CV mortality | ACS, CCS | Ma et.al, 2022^5^ | 8 | 0.90 (0.61, 1.34) | Low |  | - | - | - |
| CV mortality | CHD | Niu et.al, 2022^27^ | 3 | 0.87 (0.58, 1.30) | High |  | - | - | - |
| CV mortality | ACS, CCS, surgery (PCI) | Xu et.al, 2022^15^ | 4 | 0.72 (0.51, 1.01) | High |  | - | - | - |
| CV mortality | CHD, stroke | Fiolet et.al, 2024^29^ | 4 | 0.86 (0.61, 1.21) | High |  | - | - | - |
| CV mortality | CHD | Chen et.al, 2023^17^ | 3 | 0.82 (0.54, 1.24) | High |  | - | - | - |
| CV mortality | CHD | Bytyçi et.al, 2022^33^ | - | - | - |  | 4 | 0.89 (0.50, 1.56) | High |
| Non-CV mortality | CHD | Akl et.al, 2024^25^ | 4 | 1.48 (0.92, 2.37) | High |  | - | - | - |
| Non-CV mortality | ACS, CCS | Ma et.al, 2022^5^ | 5 | 1.34 (0.91, 1.99) | High |  | - | - | - |
| Non-CV mortality | CHD | Sen et.al, 2021^14^ | 3 | 1.42 (1.01, 1.99) | High |  | - | - | - |
| Non-CV mortality | CHD, stroke | Fiolet et.al, 2024^29^ | 4 | 1.24 (0.96, 1.61) | High |  | - | - | - |
| Non-CV mortality | ACS, CCS | Xu et.al, 2022^15^ | 3 | 1.32 (0.95, 1.85) | High |  | - | - | - |
| Non-CV mortality | CHD | Chen et.al, 2022^22^ | 4 | 1.33 (0.95, 1.86) | High |  | - | - | - |
| **Cardiovascular disorders** |  |  |  |  |  |  |  |  |  |
| ACS | ACS, CCS | Abrantes et.al, 2021^2^ | 6 | 0.65 (0.43, 0.97) | Moderate |  | - | - | - |
| ACS | CHD | Chen et.al, 2023^17^ | 3 | 0.62 (0.25, 1.50) | High |  | - | - | - |
| ACS | ACS, CCS | Akl et.al, 2024^25^ | - | - | - |  | 3 | 0.60 (0.37, 0.97) | Moderate |
| ACS (new) | CHD | Chen et.al, 2023^17^ | 4 | 0.65 (0.48, 0.87) | High |  | 4 | 0.56 (0.28, 1.11) | High |
| CHD | CHD | Andreis et.al, 2021^7^ | - | - | - |  | 6 | 0.71 (0.51, 0.99) | High |
| MI | CHD | Akl et.al, 2024^25^ | 4 | 0.68 (0.46, 1.00) | Moderate |  | - | - | - |
| MI | CHD | Niu et.al, 2022^27^ | 3 | 0.75 (0.52, 1.07) | Moderate |  | - | - | - |
| MI | ACS, CHD | Samuel et.al, 2021^35^ | 3 | 0.80 (0.62, 1.03) | Moderate |  | - | - | - |
| MI | CHD | Al-Atta et.al, 2021^38^ | 3 | 0.67 (0.44, 1.01) | Moderate |  | - | - | - |
| MI | CHD | Xia et.al, 2021^13^ | 3 | 0.73 (0.51, 1.03) | Moderate |  | - | - | - |
| MI | ACS, CCS | Grajek et.al, 2021^9^ | 3 | 0.72 (0.49, 1.05) | Moderate |  | - | - | - |
| MI | CHD | Andreis et.al, 2022^4^ | 5 | 0.71 (0.52, 0.98) | High |  | - | - | - |
| MI | ACS, CCS | Ma et.al, 2022^5^ | 7 | 0.53 (0.37, 0.78) | Moderate |  | - | - | - |
| MI | CHD | Chen et.al, 2022^22^ | 4 | 0.71 (0.49, 1.03) | High |  | 3 | 0.81 (0.51, 1.31) | High |
| MI (acute) | CHD | Xiang et.al, 2021^6^ | 3 | 0.73 (0.51, 1.04) | Moderate |  | - | - | - |
| MI (recurrent) | CHD | Bytyçi et.al, 2022^33^ | 6 | 0.77 (0.63, 0.94) | High |  | - | - | - |
| MI (recurrent) | ACS, CCS | Wang et.al, 2021^30^ | 3 | 0.71 (0.47, 1.06) | Low |  | - | - | - |
| MI (recurrent) | ACS | Bao et.al, 2022^18^ | 3 | 0.48 (0.18, 1.30) | Moderate |  | 4 | 0.98 (0.58, 1.64) | High |
| MI (recurrent) | ACS | Younas et.al, 2024^19^ | 4 | 0.34 (0.13, 0.89) | High |  | - | - | - |
| POAF | CHD | Teo et.al, 2021^42^ | - | - | - |  | 3 | 0.73 (0.51, 1.04) | High |
| POAF (< 1 m) | Surgery (aortic, CABG, MR) | Zhao et.al, 2022^41^ | - | - | - |  | 4 | 0.61 (0.44, 0.84) | High |
| **Hospitalization** |  |  |  |  |  |  |  |  |  |
| Hospitalization | ACS, CCS | Abrantes et.al, 2021^2^ | 3 | 0.78 (0.53, 1.14) | Low |  | - | - | - |
| **MACEs** |  |  |  |  |  |  |  |  |  |
| Adverse cardiovascular events a | ACS | Younas et.al, 2024^19^ | 5 | 0.73 (0.48, 1.11) | Moderate |  | - | - | - |
| All-cause mortality, cardiac arrest, ISR, MI, stent thrombosis and stroke | Surgery (PCI) | Wei et.al, 2023^23^ | - | - | - |  | 3 | 0.54 (0.38, 0.78) | High |
| All-cause mortality, cardiac arrest, ISR, MI, stent thrombosis and stroke | Surgery (PCI) | Wei et.al, 2023^23^ | - | - | - |  | 4 | 0.83 (0.61, 1.13) | High |
| All-cause mortality, CV mortality, MI and stroke | ACS, CCS | Abrantes et.al, 2021^2^ | 3 | 0.65 (0.44, 0.96) | Low |  | 3 | 0.59 (0.37, 0.95) | Moderate |
| All-cause mortality, CV mortality, MI and stroke | CHD | Xia et.al, 2021^13^ | 3 | 0.64 (0.47, 0.87) | Moderate |  | - | - | - |
| All-cause mortality, CV mortality, recurrent MI and stroke | CHD | Bytyçi et.al, 2022^33^ | 5 | 0.63 (0.50, 0.81) | Moderate |  | - | - | - |
| All-cause mortality, ACS, CV mortality, revascularization and stroke | CHD | Shrestha, 2022^3^ | 3 | 0.61 (0.43, 0.86) | Low |  | - | - | - |
| All-cause mortality, CV mortality and stroke | CHD | Aw et.al, 2022^24^ | - | - | - |  | 5 | 0.69 (0.52, 0.91) | High |
| All-cause mortality, HF, MI, revascularization and stroke | ACS | Zhou et.al, 2023^21^ | - | - | - |  | 3 | 0.68 ( 0.46, 1.01) | Low |
| All-cause mortality, HF, MI, revascularization and stroke (≤ 3 d) | ACS | Zhou et.al, 2023^21^ | - | - | - |  | 3 | 0.68 (0.46, 1.01) | High |
| CV mortality, coronary revascularization, MI and stroke | ACS, CCS, surgery (PCI) | Xu et.al, 2022^15^ | 5 | 0.66 (0.51, 0.85) | High |  | - | - | - |
| CV mortality, coronary revascularization, MI and stroke | CHD, stroke | Fiolet et.al, 2024^29^ | 4 | 0.73 (0.62, 0.85) | High |  | - | - | - |
| CV mortality, coronary revascularization, MI and stroke | CHD | Al-Atta et.al, 2021^38^ | 3 | 0.65 (0.48, 0.87) | Moderate |  | - | - | - |
| CV mortality, MI and stroke | ACS, CCS | Ma et.al, 2022^5^ | 6 | 0.53 (0.37, 0.76) | Moderate |  | - | - | - |
| CV mortality, MI and stroke | CHD | Akl et.al, 2024^25^ | 3 | 0.61 (0.44, 0.85) | Moderate |  | - | - | - |
| CV mortality, MI and stroke | ACS, CCS | Akl et.al, 2024^25^ | 3 | 0.94 (0.60, 1.48) | Moderate |  | - | - | - |
| CV mortality, MI and stroke | Atherosclerosis | Fiolet et.al, 2021^45^ | 3 | 0.73 (0.57, 0.95) | High |  | - | - | - |
| CV mortality, MI,PCI, revascularization and stroke | ACS, CCS | Grajek et.al, 2021^9^ | 3 | 1.08 (0.86, 1.35) | High |  | - | - | - |
| Major CV events **^b^** | CHD with diabetes | Kuzemczak et.al, 2021^46^ | 3 | 0.67 (0.44, 1.03) | Moderate |  | - | - | - |
| Major CV events **^b^** | CHD without diabetes | Kuzemczak et.al, 2021^46^ | 3 | 0.66 (0.47, 0.93) | Moderate |  | - | - | - |
| Major adverse CV and cerebrovascular events | CHD | Andreis et.al, 2022^4^ | 6 | 0.61 (0.48, 0.79) | Moderate |  | 5 | 0.85 (0.52, 1.34) | High |
| **Revascularization** |  |  | - | - | - |  |  |  |  |
| Revascularization | ACS, CCS | Grajek et.al, 2021^9^ | 4 | 0.59 (0.41, 0.85) | High |  | - | - | - |
| Revascularization | Atherosclerosis | Fiolet et.al, 2021^45^ | 4 | 0.80 (0.69, 0.92) | High |  | - | - | - |
| Revascularization | CHD | Chen et.al, 2023^17^ | 4 | 0.66 (0.53, 0.83) | Moderate |  | 3 | 0.38 (0.16, 0.89) | High |
| Revascularization | ACS, CCS | Grajek et.al, 2021^9^ | 3 | 0.60 (0.40, 0.87) | Moderate |  | - | - | - |
| Revascularization | ACS, CCS, surgery (PCI) | Xu et.al, 2022^15^ | 3 | 0.44 (0.21, 0.93) | High |  | - | - | - |
| Revascularization | CHD | Andreis et.al, 2022^4^ | 3 | 0.58 (0.37, 0.92) | Moderate |  | - | - | - |
| Repeat vessel revascularization | CHD | Aw et.al, 2022^24^ | - | - | - |  | 3 | 0.40 (0.17, 0.91) | High |
| **Stroke** |  |  |  |  |  |  |  |  |  |
| Ischaemic stroke | CHD | Xiang et.al, 2021^6^ | 4 | 0.47 (0.23, 0.98) | High |  | - | - | - |
| Ischaemic stroke | ACS, CCS | Samuel et.al, 2021^35^ | 3 | 0.39 (0.12, 0.67) | Moderate |  | - | - | - |
| Ischaemic stroke | CHD, stroke | Fiolet et.al, 2024^29^ | 4 | 0.65 (0.43, 0.98) | High |  | - | - | - |
| Stroke | ACS, CCS | Ma et.al, 2022^5^ | 5 | 0.50 (0.30, 0.83) | High |  | - | - | - |
| Stroke | ACS, CHD | Akl et.al, 2024^25^ | 4 | 0.45 (0.25, 0.82) | High |  | 4 | 0.90 (0.06, 12.73) | Low |
| Stroke | ACS, AIS, CCS | Masson et.al, 2020^26^ | - | - | - |  | 4 | 0.80 (0.15, 4.36) | Moderate |
| Stroke | ACS, CCS | Xu et.al, 2022^15^ | 3 | 0.82 (0.54, 1.24) | Moderate |  | - | - | - |
| Stroke | CHD | Aw et.al, 2022^24^ | 3 | 0.44 (0.19, 1.01) | High |  | 4 | 0.62 (0.19, 2.08) | High |
| Stroke | CHD | Chen et.al, 2023^17^ | 3 | 0.61 (0.33, 1.13) | Moderate |  | 4 | 0.70 (0.20, 2.38) | High |
| Stroke | ACS | Bao et.al, 2022^18^ | 4 | 0.46 (0.27, 0.77) | High |  | 3 | 0.64 (0.21, 2.02) | High |
| **Other Outcomes** |  |  |  |  |  |  |  |  |  |
| CRP | ACS | Zhou et.al, 2023^21^ | - | - | - |  | 4 | 0.57 (0.36, 0.91) | Low |
| Hs-CRP | ACS, CCS | Grajek et.al, 2021^9^ | 4 | 0.74 (0.49, 1.13) | Moderate |  | - | - | - |
| Hs-CRP | ACS | Younas et.al, 2024^19^ | 4 | 0.61 (0.40, 0.94) | low |  | - | - | - |
| ACS, acute coronary syndrome; AIS, acute ischaemic stroke; CABG, coronary artery bypass grafting; CCS, chronic coronary syndromes; CHD, coronary heart disease; CI, confidence interval; CRP, C-reactive protein; CV, cardiovascular; d, day; GRADE, Grading of Recommendations, Assessment, Development and Evaluation; Hs-CRP, high-sensitive C-reactive protein; ISR, in-stent restenosis; MACEs, major adverse cardiac events; MI, myocardial infarction; MR, myocardial revascularization; No., number; PCI, percutaneous coronary intervention; POAF, postoperative atrial fibrillation; PROBE, Prospective, Randomized, Open-label, Blinded Endpoint; RCTs, randomised controlled trials. | | | | | | | | | |
| ^a^ Adverse cardiovascular events: ACS, CV mortality, HF, MI, resuscitated cardiac arrest, stroke, UA, urgent hospitalization for angina and ventricular arrhythmias. | | | | | | | | | |
| ^b^ Major CV events: ACS, out-of-hospital cardiac arrest, CV mortality, resuscitated cardiac arrest, MI, stroke, or urgent hospitalization for angina, leading to coronary revascularization, ischaemic stroke, or ischemia-driven coronary revascularization. | | | | | | | | | |

**Supplementary Table S10. Subgroup analyses according to the duration of colchicine use on assessed associations**

| **Outcomes** | **Patients** | **Author, year,  reference** | **Shorter duration of colchicine (≤ 1 month)** | | |  | **Longer duration of colchicine (> 1 month)** | | |
| --- | --- | --- | --- | --- | --- | --- | --- | --- | --- |
|  |  |  | **No. of RCTs** | **Random effect size (95% CI)** | **GRADE** |  | **No. of RCTs** | **Random effect size (95% CI)** | **GRADE** |
| **Adverse events** |  |  |  |  |  |  |  |  |  |
| Adverse events | ACS | Abrantes et.al, 2021^2^ | - | - | - |  | 5 | 1.13 (0.90, 1.42) | Moderate |
| Drug discontinuation | CHD | Papageorgiou et.al, 2017^8^ | 4 | 8.45 (3.05, 23.41) | High |  | - | - | - |
| Drug discontinuation | ACS, CCS | Grajek et.al, 2021^9^ | 3 | 1.56 (0.34, 7.13) | High |  | 6 | 1.36 (0.98, 1.92) | Low |
| Drug discontinuation | CHD | Kofler et.al, 2021^11^ | 5 | 2.50 (0.78, 8.00) | High |  | 6 | 1.43 (0.99, 2.07) | Very low |
| Drug discontinuation | CHD | Andreis et.al, 2021^7^ | 3 | 2.95 (1.11, 7.86) | Moderate |  | 5 | 1.37 (0.98, 1.93) | Moderate |
| Drug discontinuation (without PROBE studies) | ACS, CCS | Grajek et.al, 2021^9^ | - | - | - |  | 6 | 1.36 (0.97, 1.91) | Low |
| Gastrointestinal adverse events | CHD | Shrestha, 2022^3^ | 4 | 4.49 (0.83, 24.2) | Moderate |  | 5 | 1.12 (0.88, 1.43) | Moderate |
| Gastrointestinal adverse events | ACS, CCS | Abrantes et.al, 2021^2^ | 3 | 2.99 (1.14, 7.86) | Low |  | 6 | 1.48 (0.10, 2.18) | Very low |
| Gastrointestinal adverse events | ACS, CCS, surgery (PCI) | Xu et.al, 2022^15^ | - | - | - |  | 4 | 1.06 (0.90, 1.24) | High |
| Gastrointestinal adverse events | ACS, CCS | Ma et.al, 2022^5^ | 7 | 2.91 (1.86, 4.58) | High |  | 7 | 1.50 (1.06, 2.14) | Very low |
| Gastrointestinal adverse events | CHD | Kofler et.al, 2021^11^ | 6 | 3.75 (2.02, 6.97) | High |  | 6 | 1.44 (0.98, 2.13) | Low |
| Gastrointestinal adverse events | CHD | Tien et.al, 2021^16^ | 4 | 4.36 (1.75, 10.87) | High |  | 4 | 3.82 (1.02, 14.27) | Very low |
| Gastrointestinal adverse events | CHD | Chen et.al, 2023^17^ | 6 | 3.53 (1.88, 6.62) | High |  | 6 | 1.43 (0.97, 2.09) | Low |
| Gastrointestinal adverse events | ACS | Bao et.al, 2022^18^ | 6 | 2.81 (1.77, 4.46) | High |  | 3 | 1.12 (0.83, 1.50) | Moderate |
| Gastrointestinal adverse events | ACS | Younas et.al, 2024^19^ | 6 | 2.97 (1.25, 7.06) | Very low |  | - | - | - |
| Gastrointestinal adverse events | ACS | Diaz-Arocutipa et.al, 2021^20^ | 3 | 6.52 (1.16, 36.60) | Moderate |  | - | - | - |
| Gastrointestinal adverse events | CHD | Andreis et.al, 202115 | 4 | 2.97 (1.69, 5.21) | High |  | 6 | 1.40 (1.02, 1.94) | Low |
| Gastrointestinal adverse events | ACS | Zhou et.al, 2023^21^ | 4 | 4.00 (1.75, 9.16) | High |  | - | - | - |
| Gastrointestinal adverse events | CHD | Chen et.al, 2022^22^ | 5 | 3.05 (1.65, 5.66) | High |  | 5 | 1.51 (0.98, 2.31) | Very low |
| Gastrointestinal adverse events | CHD | Andreis et.al, 2021^7^ | 3 | 3.87 (1.19, 12.60) | Moderate |  | - | - | - |
| Gastrointestinal adverse events | Surgery (aortic, CABG) | Agarwal et.al, 2023^12^ | 3 | 1.62 (1.21, 2.18) | High |  | - | - | - |
| Postoperative adverse events | PCI | Wei et.al, 2023^23^ | - | - | - |  | 3 | 1.40 (0.94, 2.09) | Low |
| **All-cause and cause-specific mortality** |  |  |  |  |  |  |  |  |  |
| All-cause mortality | ACS, AIS, surgery (PCI) | Masson et.al, 2020^26^ | - | - | - |  | 5 | 0.84 (0.58, 1.22) | High |
| All-cause mortality | ACS, CCS, surgery (PCI) | Aw et.al, 2022^24^ | - | - | - |  | 4 | 1.20 (0.40, 3.57) | High |
| All-cause mortality | ACS, CCS | Grajek et.al, 2021^9^ | - | - | - |  | 7 | 0.98 (0.64, 1.49) | High |
| All-cause mortality | ACS, CCS | Wang et.al, 2021^30^ | - | - | - |  | 3 | 1.21 (0.76, 1.92) | Moderate |
| All-cause mortality | ACS, CCS | Ma et.al, 2022^5^ | - | - | - |  | 7 | 1.04 (0.67, 1.63) | High |
| All-cause mortality | CHD | Kofler et.al, 2021^11^ | - | - | - |  | 6 | 0.99 (0.60, 1.62) | High |
| All-cause mortality | CHD | Liao et.al, 2021^32^ | - | - | - |  | 3 | 0.98 (0.65, 1.48) | High |
| All-cause mortality | ACS | Bao et.al, 2022^18^ | - | - | - |  | 3 | 1.83 (0.58, 5.74) | Moderate |
| All-cause mortality | ACS | Younas et.al, 2024^19^ | 4 | 1.00 (0.72, 1.39) | High |  | - | - | - |
| All-cause mortality | ACS | Diaz-Arocutipa et.al, 2021^20^ | 3 | 0.98 (0.13, 6.91) | Moderate |  | - | - | - |
| All-cause mortality | CHD | Andreis et.al, 2022^4^ | - | - | - |  | 7 | 0.98 (0.63, 1.54) | High |
| All-cause mortality | CHD | Xiang et.al, 2021^6^ | 4 | 0.77 (0.62, 0.97) | Moderate |  | 4 | 0.53 (0.30, 0.92) | Moderate |
| All-cause mortality | CHD | Bytyçi et.al, 2022^33^ | - | - | - |  | 5 | 1.04 (0.64, 1.69) | High |
| CV mortality | CHD | Kofler et.al, 2021^11^ | - | - | - |  | 4 | 0.86 (0.57, 1.29) | High |
| CV mortality | CHD | Akl et.al, 2024^25^ | - | - | - |  | 5 | 0.76 (0.42, 1.36) | Moderate |
| CV mortality | ACS, CCS | Grajek et.al, 2021^9^ | - | - | - |  | 5 | 0.84 (0.57, 1.23) | High |
| CV mortality | ACS, CCS | Abrantes et.al, 2021^2^ | - | - | - |  | 5 | 0.83 (0.56, 1.24) | High |
| CV mortality | ACS, CCS, surgery (PCI) | Xu et.al, 2022^15^ | - | - | - |  | 4 | 0.73 (0.55, 0.98) | High |
| CV mortality | CHD | Chen et.al, 2023^17^ | - | - | - |  | 3 | 0.85 (0.56, 1.29) | Moderate |
| CV mortality | ACS, CCS | Wang et.al, 2021^30^ | - | - | - |  | 3 | 0.86 (0.57, 1.30) | High |
| CV mortality | CHD | Chen et.al, 2022^22^ | - | - | - |  | 4 | 0.87 (0.58, 1.32) | Moderate |
| CV mortality | ACS | Bao et.al, 2022^18^ | - | - | - |  | 3 | 1.02 (0.57, 1.81) | Moderate |
| CV mortality | CHD | Andreis et.al, 2022^4^ | - | - | - |  | 6 | 0.61 (0.29, 1.28) | High |
| CV mortality | CHD | Bytyçi et.al, 2022^33^ | - | - | - |  | 3 | 0.25 (0.03, 2.03) | Moderate |
| Non-CV mortality | CHD | Akl et.al, 2024^25^ | - | - | - |  | 4 | 1.83 (0.98, 3.41) | Low |
| Non-CV mortality | ACS, CCS | Ma et.al, 2022^5^ | - | - | - |  | 5 | 1.33 (0.93, 1.90) | High |
| **Cardiovascular disorders** |  |  |  |  |  |  |  |  |  |
| ACS | ACS, CCS | Abrantes et.al, 2021^2^ | 3 | 0.53 (0.09, 3.32) | Moderate |  | 4 | 0.62 (0.42, 0.92) | Moderate |
| ACS | ACS | Alberto et.al, 2021^36^ | - | - | - |  | 3 | 0.56 (0.42, 0.76) | High |
| ACS (new) | CHD | Chen et.al, 2023^17^ | 4 | 0.47 (0.09, 2.33) | High |  | 3 | 0.72 (0.60, 0.87) | High |
| CHD | CHD | Andreis et.al, 2021^7^ | - | - | - |  | 5 | 0.72 (0.63, 0.82) | High |
| MI | CHD | Sattar et.al, 2022^28^ | - | - | - |  | 4 | 0.68 (0.48, 0.96) | Moderate |
| MI | CHD | Akl et.al, 2024^25^ | - | - | - |  | 4 | 0.72 (0.53, 0.97) | High |
| MI | CHD | Al-Atta et.al, 2021^38^ | - | - | - |  | 3 | 0.67 (0.44, 1.01) | Moderate |
| MI | ACS, CCS | Grajek et.al, 2021^9^ | 3 | 0.26 (0.04, 1.67) | High |  | 4 | 0.72 (0.52, 1.00) | High |
| MI | ACS, CCS | Ma et.al, 2022^5^ | 4 | 0.89 (0.55, 1.46) | High |  | 5 | 0.53 (0.35, 0.80) | Very low |
| MI | ACS | Zhou et.al, 2023^21^ | 3 | 0.50 (0.15, 1.69) | High |  | - | - | - |
| MI (recurrent) | CHD | Bytyçi et.al, 2022^33^ | 3 | 0.84 (0.50, 1.41) | High |  | 4 | 0.74 (0.57, 0.96) | High |
| MI (recurrent) | ACS, CCS | Wang et.al, 2021^30^ | - | - | - |  | 4 | 0.71 (0.51, 1.00) | High |
| MI (recurrent) | ACS | Bao et.al, 2022^18^ | 4 | 0.66 (0.25, 1.73) | Moderate |  | 3 | 0.75 (0.46, 1.23) | High |
| MI (recurrent) | ACS | Younas et.al, 2024^19^ | 5 | 0.75 (0.51, 1.10) | High |  | - | - | - |
| POAF | Surgery (CABG) | Kirov et.al, 2024^40^ | 3 | 0.52 (0.37, 0.73) | High |  | - | - | - |
| POAF | Surgery (aortic, CABG) | Agarwal et.al, 2023^12^ | 4 | 0.64 (0.50, 0.83) | Moderate |  | - | - | - |
| **Hospitalization** |  |  |  |  |  |  |  |  |  |
| Hospitalization | ACS, CCS | Abrantes et.al, 2021^2^ | - | - | - |  | 3 | 0.90 (0.76, 1.07) | High |
| **MACEs** |  |  |  |  |  |  |  |  |  |
| ACS, AF, mortality, revascularization and stroke | Surgery (CABG, PCI) | Chen et.al, 2023^17^ | 3 | 0.86 (0.49, 1.51) | High |  | - | - | - |
| ACS, CHD, post-angioplasty and stroke | CHD | Verma et.al, 2015^10^ | - | - | - |  | 3 | 0.39 (0.23, 0.65) | High |
| Adverse cardiovascular events a | ACS | Younas et.al, 2024^19^ | 5 | 0.68 (0.52, 0.90) | High |  | - | - | - |
| All-cause mortality, cardiac arrest, ISR, MI, stent thrombosis and stroke | Surgery (PCI) | Wei et.al, 2023^23^ | - | - | - |  | 3 | 0.66 (0.50, 0.87) | High |
| All-cause mortality, cardiac arrest, ISR, MI, stent thrombosis and stroke | Surgery (PCI) | Wei et.al, 2023^23^ | - | - | - |  | 3 | 0.64 (0.36, 1.14) | Moderate |
| All-cause mortality and stroke (post surgery) | Surgery (aortic, CABG, MR) | Ge et.al, 2022^39^ | 3 | 1.22 (0.53, 2.84) | High |  | - | - | - |
| All-cause mortality, CV mortality, MI and stroke | ACS, CCS | Abrantes et.al, 2021^2^ | - | - | - |  | 4 | 0.64 (0.47, 0.88) | Moderate |
| All-cause mortality, CV mortality and stroke | CHD | Aw et.al, 2022^24^ | - | - | - |  | 5 | 0.70 (0.57, 0.86) | High |
| All-cause mortality, HF, MI, revascularization and stroke | ACS | Zhou et.al, 2023^21^ | 3 | 0.58 (0.31, 1.07) | High |  | - | - | - |
| All-cause mortality, recurrent MI and stroke | ACS, CCS | Wang et.al, 2021^30^ | - | - | - |  | 3 | 0.82 (0.70, 0.95) | High |
| CV mortality, coronary revascularization, MI and stroke | ACS, CCS, surgery (PCI) | Xu et.al, 2022^15^ | - | - | - |  | 4 | 0.63 (0.48, 0.82) | Moderate |
| CV mortality, coronary revascularization, MI and stroke | ACS, CCS, surgery (PCI) | Xu et.al, 2022^15^ | - | - | - |  | 5 | 0.64 (0.50, 0.82) | Moderate |
| CV mortality, coronary revascularization, MI and stroke | CHD, stroke | Fiolet et.al, 2024^29^ | - | - | - |  |  |  |  |
| CV mortality, coronary revascularization, MI and stroke | CHD | Al-Atta et.al, 2021^38^ | - | - | - |  | 3 | 0.65 (0.48, 0.87) | Moderate |
| CV mortality, MI and stroke | CHD | Akl et.al, 2024^25^ | - | - | - |  | 4 | 0.61 (0.47, 0.80) | Moderate |
| CV mortality, MI and stroke | ACS, CCS | Ma et.al, 2022^5^ | - | - | - |  | 5 | 0.51 (0.34, 0.75) | Very low |
| CV mortality, MI and stroke | ACS, CCS | Akl et.al, 2024^25^ | - | - | - |  | 4 | 0.64 (0.47, 0.88) | Moderate |
| CV mortality, MI,PCI, revascularization and stroke | ACS, CCS | Grajek et.al, 2021^9^ | - | - | - |  | 4 | 0.64 (0.49, 0.82) | Moderate |
| Major adverse CV and cerebrovascular events | CHD | Andreis et.al, 2022^4^ | 4 | 0.69 (0.37, 1.30) | High |  | 7 | 0.65 (0.53, 0.80) | High |
| **Revascularization** |  |  |  |  |  |  |  |  |  |
| Revascularization | CHD | Xiang et.al, 2021^6^ | 3 | 0.51 (0.32,0.81) | Moderate |  | - | - | - |
| Revascularization | ACS, CCS | Akl et.al, 2024^25^ | - | - | - |  | 3 | 0.58 (0.38, 0.88) | Low |
| Revascularization | ACS, CCS | Grajek et.al, 2021^9^ | - | - | - |  | 5 | 0.57 (0.40, 0.81) | High |
| Revascularization | CHD | Chen et.al, 2023^17^ | - | - | - |  | 5 | 0.61 (0.45, 0.82) | High |
| **Stroke** |  |  |  |  |  |  |  |  |  |
| Ischaemic stroke | CHD | Xiang et.al, 2021^6^ | - | - | - |  | 3 | 0.65 (0.39, 1.19) | High |
| Ischaemic stroke | CHD | Chen et.al, 2022^22^ | - | - | - |  | 4 | 0.46 (0.27, 0.77) | High |
| Non-cardio-embolic ischaemic stroke | CHD | Shrestha, 2022^3^ | - | - | - |  | 5 | 0.49 (0.29, 0.81) | High |
| Stroke | ACS, CCS | Ma et.al, 2022^5^ | - | - | - |  | 4 | 0.47 (0.29, 0.78) | High |
| Stroke | ACS, CCS | Abrantes et.al, 2021^2^ | - | - | - |  | 5 | 0.47 (0.27, 0.81) | High |
| Stroke | CHD | Katsanos et.al, 2020^48^ | - | - | - |  | 3 | 0.31 (0.12, 0.78) | High |
| Stroke | ACS, CHD | Akl et.al, 2024^25^ | - | - | - |  | 5 | 0.47 (0.28, 0.79) | Moderate |
| Stroke | ACS, AIS, CCS | Masson et.al, 2020^26^ | 3 | 0.80 (0.15, 4.36) | Moderate |  | - | - | - |
| Stroke | CHD | Al-Atta et.al, 2021^38^ | - | - | - |  | 3 | 0.43 (0.21, 0.91) | High |
| Stroke | CHD | Aw et.al, 2022^24^ | - | - | - |  | 5 | 0.47 (0.27, 0.82) | High |
| Stroke | CHD | Chen et.al, 2023^17^ | - | - | - |  | 4 | 0.32 (0.15, 0.69) | High |
| Stroke | ACS, CCS | Wang et.al, 2021^30^ | - | - | - |  | 4 | 0.46 (0.27, 0.76) | High |
| Stroke | ACS | Bao et.al, 2022^18^ | 3 | 1.30 (0.25, 6.81) | High |  | - | - | - |
| Stroke | CHD | Andreis et.al, 2022^4^ | - | - | - |  | 5 | 0.46 (0.29, 0.76) | High |
| Stroke | ACS | Younas et.al, 2024^19^ | 3 | 0.65 (0.17, 2.43) | High |  | - | - | - |
| Stroke | CHD | Bytyçi et.al, 2022^33^ | 3 | 0.43 (0.12,1.59) | High |  | 4 | 0.47 (0.28. 0.80) | High |
| **Other Outcomes** |  |  |  |  |  |  |  |  |  |
| CRP | ACS | Zhou et.al, 2023^21^ | 4 | 0.59 (0.28, 1.25) | Very low |  | - | - | - |
| Hs-CRP | ACS, CCS | Grajek et.al, 2021^9^ | 5 | 0.60 (0.33, 1.63) | High |  | - | - | - |
| Hs-CRP | ACS | Younas et.al, 2024^19^ | 6 | 0.46 (0.22, 0.94) | Moderate |  | - | - | - |
| ACS, acute coronary syndrome; AIS, acute ischaemic stroke; CABG, coronary artery bypass grafting; CCS, chronic coronary syndromes; CHD, coronary heart disease; CI, confidence interval; CRP, C-reactive protein; CV, cardiovascular; d, day; GRADE, Grading of Recommendations, Assessment, Development and Evaluation; Hs-CRP, high-sensitive C-reactive protein; ISR, in-stent restenosis; MACEs, major adverse cardiac events; MI, myocardial infarction; MR, myocardial revascularization; No., number; PCI, percutaneous coronary intervention; POAF, postoperative atrial fibrillation; PROBE, Prospective, Randomized, Open-label, Blinded Endpoint; RCTs, randomised controlled trials. | | | | | | | | | |
| ^a^ Adverse cardiovascular events: ACS, CV mortality, HF, MI, resuscitated cardiac arrest, stroke, UA, urgent hospitalization for angina and ventricular arrhythmias. | | | | | |  |  |  |  |
| ^b^ Major CV events: ACS, out-of-hospital cardiac arrest, CV mortality, resuscitated cardiac arrest, MI, stroke, or urgent hospitalization for angina, leading to coronary revascularization, ischaemic stroke, or ischemia-driven coronary revascularization. | | | | | | | | | |

| **Supplementary Table S11. Subgroup analysis according to the region of colchicine use on assessed associations** | | | | | | | | | | | | | | | |  |
| --- | --- | --- | --- | --- | --- | --- | --- | --- | --- | --- | --- | --- | --- | --- | --- | --- |
| **Outcomes** | **Intervention Dose (mg/d)** | **Intervention Duration (month)** | **Patients** | **Americas** | | | **Oceania** | | | **Europe** | | | **Asia** | | |  |
|  |  |  |  | **No.**  **of RCTs** | **Random effect size (95% CI)** | **GRADE** | **No.**  **of RCTs** | **Random effect size (95% CI)** | **GRADE** | **No.**  **of RCTs** | **Random effect size (95% CI)** | **GRADE** | **No.**  **of RCTs** | **Random effect size (95% CI)** | **GRADE** | |
|  |  |  |  |  |  |  |  |  |  |  |  |  |  |  |  |  |
| **Adverse events** |  |  |  |  |  |  |  |  |  |  |  |  |  |  |  | |
| Adverse events (in cutaneous) | 0.5 - 2.0 | 0.2 - 28.6 | CHD | - | - | - | 5 | 0.52 (0.35, 0.78) | Moderate | 3 | 0.78 (0.29, 2.09) | Moderate | - | - | - | |
| Drug discontinuation | 0.5 - 1.0 | 1.0 - 24.0 | ACS, CCS | - | - | - | 5 | 1.70 (0.80, 3.60) | Moderate | 3 | 2.21 (1.18, 4.12) | High | - | - | - | |
| Drug discontinuation | 0.5 - 1.8 | < 0.1 - 24.0 | ACS, CCS | - | - | - | 3 | 0.55 (0.36, 0.84) | Moderate | - | - | - | - | - | - | |
| Drug discontinuation | 0.5 - 1.2 | 0.2 - 36.0 | CHD | - | - | - | 5 | 1.81 (0.80, 4.09) | Moderate | 4 | 3.04 (1.26, 7.36) | High | - | - | - | |
| Drug discontinuation | 0.5 - 2.0 | 0.2 - 28.6 | CHD | - | - | - | 3 | 1.43 (0.84, 2.43) | Moderate | - | - | - | - | - | - | |
| Drug discontinuation | 1.0 | 0.2 - 6.0 | CHD | - | - | - | - | - | - | 3 | 2.48 (1.04, 5.94) | High | - | - | - |  |
| Drug discontinuation (without PROBE studies) | 0.5 - 1.0 | 1.0 - 24.0 | ACS, CCS | - | - | - | 4 | 1.30 (0.78, 2.17) | Moderate |  |  |  | - | - | - |  |
| Gastrointestinal adverse events | 0.5 - 1.0 | 0.2 - 36.0 | CHD | - | - | - | 5 | 1.24 (0.83, 1.87) | High | 3 | 8.86 (1.40, 56.20) | Moderate | - | - | - |  |
| Gastrointestinal adverse events | 0.5 - 1.0 | 1.0 - 24.0 | ACS, CCS | - | - | - | 5 | 1.62 (0.94, 2.80) | Low | - | - | - | - | - | - |  |
| Gastrointestinal adverse events | 0.5 - 1.0 | 7.1 - 28.6 | ACS, CCS, surgery (PCI) | - | - | - | 3 | 1.12 (0.91, 1.38) | High | - | - | - | - | - | - |  |
| Gastrointestinal adverse events | 0.5 - 1.8 | 0.2 - 36.0 | ACS, CCS | - | - | - | 5 | 1.49 (0.94, 2.35) | Low | 5 | 4.65 (1.77, 12.19) | High | - | - | - |  |
| Gastrointestinal adverse events | 0.5 - 1.8 | 0.2 - 36.0 | CHD | - | - | - | 5 | 1.59 (0.94, 2.71) | Low | 4 | 7.08 (1.90, 26.38) | High | - | - | - |  |
| Gastrointestinal adverse events | 0.5 - 1.8 | 0.2 - 36.0 | CHD | - | - | - | - | - | - | 3 | 7.89 (1.40, 44.59) | Low | - | - | - |  |
| Gastrointestinal adverse events | 0.5 - 2.0 | < 0.1 - 24.0 | CHD | 3 | 2.07 (0.80, 5.37) | Low | 5 | 1.58 (0.93, 2.68) | Low | 3 | 8.72 (1.29, 59.06) | Moderate | - | - | - |  |
| Gastrointestinal adverse events | 0.5 - 2.0 | < 0.1 - 24.0 | ACS | - | - | - | 3 | 1.45 (0.90, 2.33) | High | 3 | 11.06 (2.60, 46.98) | Moderate | - | - | - |  |
| Gastrointestinal adverse events | 0.5 - 2.0 | 0.2 - 19.6 | ACS | - | - | - | - | - | - | 3 | 6.74 (1.87, 24.27) | Moderate | - | - | - |  |
| Gastrointestinal adverse events | 0.5 - 2.0 | 0.2 - 36.0 | CHD | - | - | - | 3 | 3.33 (0.99, 11.15) | Moderate | 3 | 6.81 (1.26, 36.72) | Low | - | - | - |  |
| Gastrointestinal adverse events | 1.0 - 2.0 | 0.2 - 6.0 | CHD | - | - | - | - | - | - | 3 | 3.95 (1.34, 11.58) | High | - | - | - |  |
| Gastrointestinal adverse events | 0.5 - 2.0 | 0.2 - 28.6 | CHD | - | - | - | 3 | 1.14 (0.91, 1.42) | High | 4 | 2.88 (1.69, 4.88) | High | - | - | - |  |
| Gastrointestinal adverse events | 1.0 - 2.0 | In-hospital - 6.0 | Surgery (aortic, CABG) | - | - | - | - | - | - | - | - | - | 3 | 3.28 (1.58, 6.81) | High |  |
| Gastrointestinal adverse events | 1.0 - 2.0 | In-hospital - 6.0 | Surgery (aortic, CABG) | - | - | - | - | - | - | 3 | 1.62 (1.21, 2.18) | High | - | - | - |  |
| **All-cause and cause-specific mortality** |  |  |  |  |  |  |  |  |  |  |  |  |  |  |  |  |
| All-cause mortality | 0.5 - 1.0 | 7.1 - 36.0 | CHD, stroke | - | - | - | 3 | 1.23 (0.45, 3.40) | Moderate | - | - | - | - | - | - |  |
| All-cause mortality | 0.5 - 1.8 | < 0.1 - 24.0 | ACS, CCS | - | - | - | 3 | 1.17 (0.35, 3.97) | Moderate | - | - | - | - | - | - |  |
| All-cause mortality | 0.5 - 1.8 | 0.2 - 36.0 | ACS, CCS | 3 | 0.53 (0.20, 1.43) | High | - | - | - | - | - | - | - | - | - |  |
| All-cause mortality | 0.5 - 1.8 | 0.2 - 36.0 | CHD | 3 | 0.50 (0.18, 1.43) | High | - | - | - | - | - | - | - | - | - |  |
| All-cause mortality | 0.5 - 2.0 | 0.2 - 28.6 | CHD | 3 | 0.95 (0.63, 1.42) | High | - | - | - | 3 | 0.79 (0.16, 3.82) | Moderate | - | - | - |  |
| All-cause mortality | 0.5 - 2.0 | 0.2 - 36.0 | CHD | - | - | - | 3 | 0.54 (0.29, 1.01) | Moderate | 3 | 0.97 (0.20, 4.73) | Low | - | - | - |  |
| All-cause mortality (fixed-effect model) | 0.5 - 1.0 | 6.0 - 36.0 | CCS | - | - | - | 4 | 0.57 (0.32, 1.00) | High | - | - | - | - | - | - |  |
| All-cause mortality (follow-up time ≥ 6 m) | 0.5 - 1.0 | 6.0 - 24.0 | CHD | - | - | - | 3 | 1.18 (0.35, 4.01) | Moderate | - | - | - | - | - | - |  |
| CV mortality | 0.5 - 1.0 | 1.0 - 28.6 | CHD | - | - | - | 3 | 0.64 (0.16, 2.59) | Moderate | - | - | - | - | - | - |  |
| CV mortality | 0.5 - 1.0 | 7.1 - 36.0 | CHD | - | - | - | 3 | 0.98 (0.39, 2.48) | Moderate | - | - | - | - | - | - |  |
| CV mortality | 0.5 - 1.0 | 7.1 - 36.0 | CHD, stroke | - | - | - | 4 | 0.81 (0.40, 1.35) | High | - | - | - | - | - | - |  |
| CV mortality | 0.5 - 1.8 | 1.0 - 36.0 | CHD | - | - | - | 3 | 0.97 (0.39, 2.47) | Moderate | - | - | - | - | - | - |  |
| Non-CV mortality | 0.5 - 1.0 | 1.0 - 28.6 | CHD | - | - | - | 3 | 3.19 (0.95, 10.74) | Moderate | - | - | - | - | - | - |  |
| Non-CV mortality | 0.5 - 1.0 | 1.0 - 36.0 | ACS, CCS | - | - | - | 3 | 1.43 (0.63, 3.26) | High | - | - | - | - | - | - |  |
| Non-CV mortality | 0.5 - 1.0 | 7.1 - 36.0 | CHD | - | - | - | 3 | 2.61 (0.75, 9.14) | High | - | - | - | - | - | - |  |
| Non-CV mortality | 0.5 - 1.0 | 7.1 - 36.0 | CHD, stroke | - | - | - | 3 | 1.48 (0.70, 2.80) | High | - | - | - | - | - | - |  |
| Non-CV mortality | 0.5 - 1.8 | 1.0 - 36.0 | CHD | - | - | - | 3 | 1.44 (0.63, 3.31) | High | - | - | - | - | - | - |  |
| **Cardiovascular disorders** |  |  |  |  |  |  |  |  |  |  |  |  |  |  |  |  |
| ACS | 0.5 - 1.0 | 1.0 - 22.0 | ACS | - | - | - | 3 | 1.03 (0.23, 4.55) | High | - | - | - | - | - | - |  |
| ACS | 0.5 - 1.0 | 1.0 - 24.0 | ACS, CCS | - | - | - | 5 | 0.52 (0.32, 0.84) | High | - | - | - | - | - | - |  |
| ACS | 0.5 - 1.0 | 1.0 - 24.0 | CHD | - | - | - | 3 | 0.54 (0.33, 0.88) | High | - | - | - | - | - | - |  |
| ACS | 0.5 - 1.0 | 1.0 - 24.0 | ACS, CCS | - | - | - | 3 | 0.55 (0.34, 0.91) | High | - | - | - | - | - | - |  |
| ACS | 0.5 - 1.0 | 1.0 - 36.0 | CHD | - | - | - | 3 | 0.44 (0.23, 0.83) | High | - | - | - | - | - | - |  |
| ACS (new) | 0.5 - 1.8 | < 0.1 - 24.0 | CHD | - | - | - | 5 | 0.57 (0.39, 0.84) | High | - | - | - | - | - | - |  |
| CHD | 0.5 - 2.0 | 0.2 - 28.6 | CHD | - | - | - | 3 | 0.70 (0.59, 0.83) | High | - | - | - | - | - | - |  |
| MI | 0.5 - 1.0 | 1.0 - 24.0 | CHD | - | - | - | 3 | 0.55 (0.34, 0.90) | High | - | - | - | - | - | - |  |
| MI | 0.5 - 1.0 | 1.0 - 36.0 | CHD | - | - | - | 4 | 0.58 (0.38, 0.89) | High | - | - | - | - | - | - |  |
| MI | 0.5 - 1.0 | 7.1 - 36.0 | CHD, ACS | - | - | - | 3 | 0.69 (0.52, 0.91) | Moderate | - | - | - | - | - | - |  |
| MI | 0.5 - 1.0 | 7.1 - 36.0 | CHD | - | - | - | 3 | 0.55 (0.34, 0.87) | Moderate | - | - | - | - | - | - |  |
| MI | 0.5 - 1.0 | 12.0 - 24.0 | CHD | - | - | - | 3 | 0.63 (0.44, 0.91) | High | - | - | - | - | - | - |  |
| MI | 0.5 - 1.0 | 12.0 - 36.0 | ACS, CCS | - | - | - | 3 | 0.59 (0.36, 0.97) | High | - | - | - | - | - | - |  |
| MI | 0.5 - 1.2 | 1.0 - 28.6 | CHD | - | - | - | 4 | 0.58 (0.37, 0.92) | High | - | - | - | - | - | - |  |
| MI | 0.5 - 1.8 | < 0.1 - 24.0 | ACS, CCS | - | - | - | 4 | 0.60 (0.39, 0.92) | High | - | - | - | - | - | - |  |
| MI | 0.5 - 1.8 | 0.2 - 36.0 | ACS, CCS | - | - | - | 5 | 0.49 (0.30, 0.81) | High | - | - | - | - | - | - |  |
| MI (recurrent) | 0.5 - 1.2 | 1.0 - 36.0 | CHD | - | - | - | 4 | 0.66 (0.51, 0.85) | High | - | - | - | - | - | - |  |
| MI (recurrent) | 0.5 - 1.8 | < 0.1 - 36.0 | ACS, CCS | - | - | - | 3 | 0.57 (0.33, 0.93) | Moderate | - | - | - | - | - | - |  |
| POAF | 1.0 - 2.0 | In-hospital - 6.0 | Surgery (aortic, CABG) | - | - | - | - | - | - | 3 | 0.69 (0.53, 0.89) | High | 3 | 0.79 (0.56, 1.12) | Moderate |  |
| **Hospitalization** |  |  |  |  |  |  |  |  |  |  |  |  |  |  |  |  |
| Hospitalization | 0.5 - 1.0 | 1.0 - 28.6 | ACS, CCS | - | - | - | 3 | 0.54 (0.20, 1.50) | Moderate | - | - | - | - | - | - |  |
| Hospitalization | 0.5 - 2.0 | 1.0 - 28.6 | CHD | - | - | - | 3 | 0.32 (0.12, 0.89) | High | - | - | - | - | - | - |  |
| Rehospitalisation | 0.5 - 1.0 | 1.0 - 22.0 | CHD | - | - | - | 3 | 0.31 (0.11, 0.90) | High | - | - | - | - | - | - |  |
| **MACEs** |  |  |  |  |  |  |  |  |  |  |  |  |  |  |  |  |
| ACS, AF, mortality, revascularization and stroke | 0.5 - 2.0 | < 0.1 - 6.0 | Surgery (CABG, PCI) | - | - | - | - | - | - | 3 | 0.74 (0.27, 2.04) | High | - | - | - |  |
| Adverse cardiovascular events a | 0.5 - 2.0 | 1.0 - 6.0 | ACS | - | - | - | - | - | - | 3 | 0.89 (0.65, 1.23) | High | - | - | - |  |
| All-cause mortality, CV mortality, MI and stroke | 0.5 - 1.0 | 6.0 - 24.0 | ACS, CCS | - | - | - | 3 | 0.55 (0.36, 0.86) | Moderate | - | - | - | - | - | - |  |
| All-cause mortality, CV mortality, recurrent MI and stroke | 0.5 - 1.2 | 1.0 - 36.0 | CHD | - | - | - | 4 | 0.54 (0.36, 0.81) | Moderate | - | - | - | - | - | - |  |
| All-cause mortality, ACS, CV mortality, revascularization and stroke | 0.5 - 1.0 | 12.0 - 36.0 | CHD | - | - | - | 3 | 0.54 (0.34, 0.87) | Moderate | - | - | - | - | - | - |  |
| All-cause mortality, HF, MI, revascularization and stroke | 0.5 - 2.0 | 0.2 - 19.6 | ACS | - | - | - | - | - | - | 3 | 0.85 (0.49, 1.49) | Moderate | - | - | - |  |
| CV mortality, coronary revascularization, MI and stroke | 0.5 | 7.1 - 36.0 | ACS, CCS, surgery (PCI) | - | - | - | 3 | 0.52 (0.33, 0.84) | Moderate | - | - | - | - | - | - |  |
| CV mortality, coronary revascularization, MI and stroke | 0.5 - 1.0 | 12.0 - 36.0 | CHD | - | - | - | 3 | 0.64 (0.33, 1.26) | High | - | - | - | - | - | - |  |
| CV mortality, coronary revascularization, MI and stroke | 0.5 - 1.0 | 7.1 - 36.0 | CHD, stroke | - | - | - | 3 | 0.60 (0.44, 0.81) | High | - | - | - | - | - | - |  |
| CV mortality, coronary revascularization, MI and stroke | 0.5 - 1.0 | 7.1 - 36.0 | CHD | - | - | - | 3 | 0.57 (0.38, 0.86) | Moderate | - | - | - | - | - | - |  |
| CV mortality, MI and stroke | ≤ 0.5 | 1.0 - 28.6 | CHD | - | - | - | 3 | 0.41 (0.25, 0.70) | High | - | - | - | - | - | - |  |
| CV mortality, MI and stroke | 0.5 - 1.0 | > 1.0 | CHD | - | - | - | 3 | 0.53 (0.34, 0.82) | Moderate | - | - | - | - | - | - |  |
| CV mortality, MI and stroke | 0.5 - 1.0 | 1.0 - 36.0 | ACS, CCS | - | - | - | 4 | 0.50 (0.29, 0.89) | Moderate | - | - | - | - | - | - |  |
| CV mortality, MI and stroke | 0.5 - 1.0 | 1.0 - 36.0 | ACS, CCS | - | - | - | 3 | 1.22 (0.41, 3.62) | Moderate | - | - | - | - | - | - |  |
| CV mortality, MI and stroke | 0.5 - 1.0 | 12.0 - 24.0 | Atherosclerosis | - | - | - | 3 | 0.68 (0.53, 0.88) | High | - | - | - | - | - | - |  |
| CV mortality, MI and stroke | 0.5 - 1.0 | 0.2 - 36.0 | ACS, CCS | - | - | - | 4 | 0.47 (0.26, 0.85) | Moderate | - | - | - | - | - | - |  |
| **Revascularization** |  |  |  |  |  |  |  |  |  |  |  |  |  |  |  |  |
| Revascularization | 0.5 - 1.0 | 1.0 - 36.0 | ACS, CCS | - | - | - | 4 | 0.51 (0.28, 0.91) | High | - | - | - | - | - | - |  |
| Revascularization | 0.5 - 1.0 | 6.0 - 24.0 | Atherosclerosis | - | - | - | 3 | 0.66 (0.45, 0.95) | High | - | - | - | - | - | - |  |
| Revascularization | 0.5 - 1.0 | 6.0 - 24.0 | CHD | - | - | - | 3 | 0.58 (0.35 ,0.96) | High | - | - | - | - | - | - |  |
| Revascularization | 0.5 - 1.0 | 6.0 - 36.0 | ACS, CCS | - | - | - | 3 | 0.52 (0.27, 0.97) | Low | - | - | - | - | - | - |  |
| **Stroke** |  |  |  |  |  |  |  |  |  | - | - | - | - | - | - |  |
| Ischaemic stroke | 0.5 - 1.0 | 1.0 - 36.0 | CHD | - | - | - | 3 | 0.61 (0.33, 1.11) | High | - | - | - | - | - | - |  |
| Ischaemic stroke | 0.5 - 1.0 | 7.1 - 36.0 | ACS, CCS | - | - | - | 3 | 0.54 (0.17, 0.91) | High | - | - | - | - | - | - |  |
| Ischaemic stroke | 0.5 - 1.0 | 7.1 - 36.0 | CHD, stroke | - | - | - | 3 | 0.59 (0.34, 1.03) | High | - | - | - | - | - | - |  |
| Ischaemic stroke | 0.5 - 1.8 | 1.0 - 36.0 | CHD | - | - | - | 3 | 0.57 (0.32, 1.01) | Moderate | - | - | - | - | - | - |  |
| Stroke | 0.5 - 1.0 | 0.2 - 36.0 | ACS, CCS | - | - | - | 4 | 0.58 (0.33, 1.02) | High | - | - | - | - | - | - |  |
| Stroke | 0.5 - 1.0 | 1.0 - 24.0 | ACS, CCS | - | - | - | 4 | 0.56 (0.32, 0.98) | High | - | - | - | - | - | - |  |
| Stroke | 0.5 - 1.0 | 7.1 - 36.0 | CHD | - | - | - | 3 | 0.59 (0.33, 1.04) | High | - | - | - | - | - | - |  |
| Stroke | 0.5 - 1.2 | 7.1 - 36.0 | CHD | - | - | - | 4 | 0.56 (0.32, 0.99) | High | - | - | - | - | - | - |  |
| Stroke | 0.5 - 1.8 | < 0.1 - 24.0 | CHD | - | - | - | 4 | 0.62 (0.36, 1.10) | High | - | - | - | - | - | - |  |
| Stroke | 0.5 - 1.8 | < 0.1 - 36.0 | ACS, CCS | - | - | - | 3 | 0.56 (0.32, 1.00) | Low | - | - | - | - | - | - |  |
| Stroke | 0.5 - 2.0 | 1.0 - 28.6 | CHD | - | - | - | 4 | 0.55 (0.31, 0.97) | High | - | - | - | - | - | - |  |
| Stroke | 0.5 - 1.0 | 12 - 36 | ACS, CCS | - | - | - | 3 | 0.57 (0.33, 1.00) | Moderate | - | - | - | - | - | - |  |
| **Other Outcomes** |  |  |  |  |  |  |  |  |  |  |  |  |  |  |  |  |
| Hs-CRP | 0.5 - 2.0 | 0.2 - 6.0 | ACS | - | - | - | - | - | - | 3 | 0.64 (0.34, 1.21) | Moderate | - | - | - |  |
| ACS, acute coronary syndrome; AF, atrial fibrillation; CABG, coronary artery bypass grafting; CCS, chronic coronary syndromes; CHD, coronary heart disease; CI, confidence interval; CV, cardiovascular; GRADE, Grading of Recommendations Assessment, Development, and Evaluation; HF, heart failure; Hs-CRP, high-sensitive C-reactive protein; m, month; MACEs, major adverse cardiac events; MI, myocardial infarction; No., number; PCI, percutaneous coronary intervention; POAF, postoperative atrial fibrillation; PROBE, Prospective, Randomized, Open-label, Blinded Endpoint; RCTs, randomised controlled trials. | | | | | | | | | | | | | | | |  |
| ^a^Adverse cardiovascular events: ACS, CV mortality, HF, MI, resuscitated cardiac arrest, stroke, UA, urgent hospitalization for angina and ventricular arrhythmias. | | | | | | | | | | | | | | | |  |

**Supplementary Table S12. Subgroup analysis according to the age of colchicine use on assessed associations**

| **Outcomes** | **Intervention Dose (mg/d)** | **Intervention Duration (month)** | **Patients** | **Author, year,  reference** | **＜ 65 years old** | | | **≥ 65 years old** | | |
| --- | --- | --- | --- | --- | --- | --- | --- | --- | --- | --- |
|  |  |  |  |  | **No. of RCTs** | **Random effect size (95% CI)** | **GRADE** | **No. of RCTs** | **Random effect size (95% CI)** | **GRADE** |
|  |  |  |  |  |  |  |  |  |  |  |
| **Adverse events** |  |  |  |  |  |  |  |  |  |  |
| Adverse events | 0.5 - 1.0 | 0.2 - 1.0 | Surgery (aortic, CABG) | Wang et.al, 2022^1^ | 3 | 2.61 (1.27, 5.34) | Moderate | - | - | - |
| Adverse events | 0.5 - 1.0 | 1.0 - 36.0 | CHD | Shrestha, 2022^3^ | 3 | 0.98 (0.85, 1.13) | High | - | - | - |
| Diarrhea | 0.5 - 1.0 | 0.2 - 7.1 | ACS, CCS | Ma et.al, 2022^5^ | 5 | 3.24 (1.30, 8.08) | Very low | - | - | - |
| Diarrhea | 0.5 - 1.0 | 1.0 - 36.0 | CHD | Xiang et.al, 2021^6^ | 4 | 2.85 (1.06, 7.62) | Very low | - | - | - |
| Drug discontinuation | 0.5 - 1.0 | 0.2 - 1.0 | CHD | Papageorgiou et.al, 2017^8^ | 3 | 6.29 (2.36, 16.74) | Moderate | - | - | - |
| Drug discontinuation | 0.5 - 1.0 | 1.0 - 24.0 | ACS, CCS | Grajek et.al, 2021^9^ | 8 | 1.61 (0.99, 2.62) | Low | - | - | - |
| Drug discontinuation | 0.5 - 1.0 | 6.0 - 24.0 | ACS, CCS | Grajek et.al, 2021^9^ | 3 | 1.47 (1.01, 2.1) | High | - | - | - |
| Drug discontinuation | 0.5 - 1.2 | 0.2 - 36.0 | CHD | Kofler et.al, 2021^11^ | 9 | 1.92 (1.10, 3.34) | Low | - | - | - |
| Drug discontinuation | 0.5 - 2.0 | 0.2 - 28.6 | CHD | Andreis et.al, 2021^7^ | 6 | 1.94 (1.22, 3.08) | Moderate | - | - | - |
| Drug discontinuation | 1.0 | 0.2 - 6.0 | CHD | Andreis et.al, 2021^7^ | 3 | 2.87 (1.31, 6.30) | High | - | - | - |
| Drug discontinuation (without PROBE studies) | 0.5 - 1.0 | 1.0 - 24.0 | ACS, CCS | Grajek et.al, 2021^9^ | 7 | 1.55 (0.95, 2.53) | Moderate | - | - | - |
| Gastrointestinal adverse events | 0.5 - 1.0 | 0.2 - 36.0 | CHD | Shrestha, 2022^3^ | 7 | 1.66 (1.00, 2.75) | Low | - | - | - |
| Gastrointestinal adverse events | 0.5 - 1.0 | 1.0 - 24.0 | ACS, CCS | Abrantes et.al, 2021^2^ | 7 | 1.75 (1.14, 2.67) | Low | - | - | - |
| Gastrointestinal adverse events | 0.5 - 1.0 | 1.0 - 36.0 | CHD | Verma et.al, 2015^10^ | 3 | 2.43 (1.56, 3.78) | High | - | - | - |
| Gastrointestinal adverse events | 0.5 - 1.0 | 6.0 - 24.0 | CHD | Xia et.al, 2021^13^ | 3 | 1.07 (0.86, 1.33) | High | - | - | - |
| Gastrointestinal adverse events | 0.5 - 1.0 | 7.1 - 28.6 | ACS, CCS, surgery (PCI) | Xu et.al, 2022^15^ | 3 | 1.03 (0.89, 1.20) | Moderate | - | - | - |
| Gastrointestinal adverse events | 0.5 - 1.8 | 0.2 - 36.0 | ACS, CCS | Ma et.al, 2022^5^ | 11 | 2.01 (1.36, 2.97) | Low | 3 | 2.77 (0.82, 9.35) | Low |
| Gastrointestinal adverse events | 0.5 - 1.8 | 0.2 - 36.0 | CHD | Kofler et.al, 2021^11^ | 9 | 2.15 (1.33, 3.48) | Low | 3 | 3.14 (0.83, 11.97) | Low |
| Gastrointestinal adverse events | 0.5 - 1.8 | 0.2 - 36.0 | CHD | Tien et.al, 2021^16^ | 6 | 3.65 (1.37, 9.76) | Very low | - | - | - |
| Gastrointestinal adverse events | 0.5 - 2.0 | < 0.1 - 24.0 | CHD | Chen et.al, 2023^17^ | 9 | 2.03 (1.27, 3.24) | Moderate | 3 | 3.04 (0.82, 11.19) | Low |
| Gastrointestinal adverse events | 0.5 - 2.0 | < 0.1 - 24.0 | ACS | Bao et.al, 2022^18^ | 8 | 1.63 (1.09, 2.44) | Low | - | - | - |
| Gastrointestinal adverse events | 0.5 - 2.0 | 0.2 - 6.0 | ACS | Younas et.al, 2024^19^ | 7 | 1.85 (1.14, 3.00) | Very low | - | - | - |
| Gastrointestinal adverse events | 0.5 - 2.0 | 0.2 - 24.0 | ACS | Diaz-Arocutipa et.al, 2021^20^ | 5 | 1.35 (0.88, 2.05) | Low | - | - | - |
| Gastrointestinal adverse events | 0.5 - 2.0 | 0.2 - 28.6 | CHD | Andreis et.al, 2021^7^ | 6 | 1.67 (1.09, 2.55) | Very low | 4 | 2.07 (1.05, 4.07) | Moderate |
| Gastrointestinal adverse events | 0.5 - 2.0 | 0.2 - 19.6 | ACS | Zhou et.al, 2023^21^ | 5 | 2.96 (1.14, 7.68) | Very low | - | - | - |
| Gastrointestinal adverse events | 0.5 - 2.0 | 0.2 - 36.0 | CHD | Chen et.al, 2022^22^ | 8 | 1.72 (1.17, 2.53) | Low | - | - | - |
| Gastrointestinal adverse events | 1.0 - 2.0 | 0.2 - 6.0 | CHD | Andreis et.al, 2021^7^ | 3 | 2.70 (1.24, 5.90) | Moderate | - | - | - |
| Gastrointestinal adverse events | 1.0 - 2.0 | In-hospital - 6.0 | Surgery (aortic, CABG) | Agarwal et.al, 2023^12^ | 4 | 2.11 (1.06, 4.21) | Moderate | - | - | - |
| Gastrointestinal adverse events | 1.0 - 2.0 | In-hospital - 6.0 | Surgery (aortic, CABG) | Agarwal et.al, 2023^12^ | 4 | 1.59 (1.19, 2.14) | High | - | - | - |
| Gastrointestinal adverse events (surgery < 1 m) | 1.0 - 2.0 | In-hospital - 50.2 | Surgery (aortic, CABG) | Agarwal et.al, 2023^12^ | 3 | 2.03 (0.78, 5.31) | Low | - | - | - |
| Postoperative adverse events | 0.5 - 1.0 | 1.0 - 12.0 | PCI | Wei et.al, 2023^23^ | 5 | 1.55 (1.04, 2.31) | Moderate | - | - | - |
| **All-cause and cause-specific mortality** |  |  |  |  |  |  |  |  |  |  |
| All-cause mortality | 0.5 - 1.0 | 1.0 - 28.6 | CHD | Akl et.al, 2024^25^ | 3 | 1.66 (0.51, 5.44) | Moderate | - | - | - |
| All-cause mortality | 0.5 - 1.0 | 6.0 - 12.0 | ACS, CCS | Niu et.al, 2022^27^ | 3 | 1.82 (0.58, 5.74) | Moderate | - | - | - |
| All-cause mortality | 0.5 - 1.0 | 6.0 - 24.0 | CHD | Sattar et.al, 2022^28^ | 4 | 1.20 (0.40, 3.59) | High | - | - | - |
| All-cause mortality | 0.5 - 1.0 | 7.1 - 36.0 | CHD, surgery (PCI) | Xu et.al, 2022^15^ | 3 | 1.62 (0.44, 5.89) | High | - | - | - |
| All-cause mortality | 0.5 - 1.0 | 7.1 - 36.0 | CHD, stroke | Fiolet et.al, 2024^29^ | - | - | - | 3 | 1.07 (0.82, 1.43) | High |
| All-cause mortality | 0.5 - 1.2 | 7.1 - 36.0 | ACS, CCS, surgery (PCI) | Aw et.al, 2022^24^ | 4 | 1.19 (0.40, 3.56) | High |  |  |  |
| All-cause mortality | 0.5 - 1.8 | < 0.1 - 24.0 | ACS, CCS | Grajek et.al, 2021^9^ | 5 | 1.04 (0.54, 2.02) | High | 3 | 0.80 (0.32, 2.01) | Moderate |
| All-cause mortality | 0.5 - 1.8 | 0.2 - 36.0 | ACS, CCS | Ma et.al, 2022^5^ | 7 | 1.02 (0.61, 1.69) | High | - | - | - |
| All-cause mortality | 0.5 - 1.8 | 0.2 - 36.0 | CHD | Kofler et.al, 2021^11^ | 6 | 0.95 (0.51, 1.77) | High | - | - | - |
| All-cause mortality | 0.5 - 2.0 | < 0.1 | Surgery (PCI) | Fu etal, 2021^31^ | 3 | 0.89 (0.59, 1.32) | Moderate | - | - | - |
| All-cause mortality | 0.5 - 2.0 | < 0.1 - 12.0 | CHD | Liao et.al, 2021^32^ | 4 | 0.92 (0.62, 1.36) | High | - | - | - |
| All-cause mortality | 0.5 - 2.0 | < 0.1 - 24.0 | ACS | Bao et.al, 2022^18^ | 4 | 1.51 (0.64, 3.6) | High | - | - | - |
| All-cause mortality | 0.5 - 2.0 | 0.2 - 6.0 | ACS | Younas et.al, 2024^19^ | 5 | 1.00 (0.72, 1.38) | High | - | - | - |
| All-cause mortality | 0.5 - 2.0 | 0.2 - 22.0 | ACS | Shrestha, 2022^3^ | 3 | 1.63 (0.44, 6.03) | High | - | - | - |
| All-cause mortality | 0.5 - 2.0 | 0.2 - 24.0 | ACS | Diaz-Arocutipa et.al, 2021^20^ | 5 | 1.05 (0.71, 1.57) | High | - | - | - |
| All-cause mortality | 0.5 - 2.0 | 0.2 - 28.6 | CHD | Andreis et.al, 2022^4^ | 6 | 1.02 (0.62, 1.68) | High | - | - | - |
| All-cause mortality | 0.5 - 2.0 | 0.2 - 36.0 | CHD | Chen et.al, 2022^22^ | 5 | 1.13 (0.49, 2.59) | High | - | - | - |
| All-cause mortality | 0.5 - 2.0 | 0.2 - 36.0 | CHD | Xiang et.al, 2021^6^ | 6 | 0.76 (0.61, 0.95) | High | - | - | - |
| All-cause mortality | 0.5 - 2.0 | 1.0 - 28.6 | CHD | Bytyçi et.al, 2022^33^ | 4 | 1.30 (0.55, 3.04) | High | - | - | - |
| All-cause mortality (folLow-up time ≥ 6 m) | 0.5 - 1.0 | 6.0 - 24.0 | CHD | Chen et.al, 2023^17^ | 4 | 1.19 (0.39, 3.64) | High | - | - | - |
| All-cause mortality (preoperative, postoperative) | 0.5 - 2.0 | < 1.0 - 6.0 | Surgery (PCI) | Wei et.al, 2023^23^ | 3 | 0.75 (0.26, 2.12) | High | - | - | - |
| CV mortality | 0.5 - 1.0 | 0.2 - 36.0 | CHD | Kofler et.al, 2021^11^ | 4 | 0.91 (0.52, 1.59) | High | - | - | - |
| CV mortality | 0.5 - 1.0 | 1.0 - 28.6 | CHD | Akl et.al, 2024^25^ | 4 | 0.87 (0.59, 1.33) | High | - | - | - |
| CV mortality | 0.5 - 1.0 | 1.0 - 36.0 | ACS, CCS | Ma et.al, 2022^5^ | 4 | 1.02 (0.60, 1.75) | Moderate | - | - | - |
| CV mortality | 0.5 - 1.0 | 1.0 - 36.0 | ACS, CCS | Grajek et.al, 2021^9^ | 4 | 0.86 (0.51, 1.46) | High | - | - | - |
| CV mortality | 0.5 - 1.0 | 6.0 - 12.0 | ACS, CCS | Niu et.al, 2022^27^ | 3 | 1.01 (0.57, 1.81) | High | - | - | - |
| CV mortality | 0.5 - 1.0 | 6.0 - 24.0 | CHD | Sattar et.al, 2022^28^ | 3 | 0.88 (0.50, 1.56) | High | - | - | - |
| CV mortality | 0.5 - 1.0 | 6.0 - 24.0 | ACS, CCS | Abrantes et.al, 2021^2^ | 4 | 0.86 (0.49, 1.48) | High | - | - | - |
| CV mortality | 0.5 - 1.0 | 6.0 - 24.0 | CHD | Xia et.al, 2021^13^ | 3 | 0.91 (0.52, 1.60) | High | - | - | - |
| CV mortality | 0.5 - 1.0 | 7.1 - 36.0 | CHD, stroke | Fiolet et.al, 2024^29^ | - | - | - | 3 | 0.83 (0.48, 1.45) | High |
| CV mortality | 0.5 - 1.8 | 1.0 - 36.0 | CHD | Chen et.al, 2022^22^ | - | - | - | 3 | 0.53 (0.15, 1.87) | High |
| CV mortality | 0.5 - 1.8 | 1.0 - 36.0 | CHD | Chen et.al, 2022^22^ | 3 | 0.94 (0.53, 1.67) | High | - | - | - |
| CV mortality | 0.5 - 2.0 | 0.2 - 24.0 | ACS | Bao et.al, 2022^18^ | 4 | 0.99 (0.58, 1.70) | High | - | - | - |
| CV mortality | 0.5 - 2.0 | 0.2 - 28.6 | CHD | Andreis et.al, 2022^4^ | 5 | 0.82 (0.47, 1.40) | High | - | - | - |
| Non-CV mortality | 0.5 - 1.0 | 7.1 - 36.0 | CHD, stroke | Fiolet et.al, 2024^29^ | - | - | - | 3 | 1.26 (0.94, 1.67) | High |
| Non-CV mortality | 0.5 - 1.8 | 1.0 - 36.0 | CHD | Chen et.al, 2022^22^ | 3 | 1.43 (0.37, 5.54) | High | - | - | - |
| **Cardiovascular disorders** |  |  |  |  |  |  |  |  |  |  |
| ACS | 0.5 - 1.0 | 1.0 - 22.0 | ACS | Ullah et.al, 2021^34^ | 12 | 0.91 (0.74, 1.12) | High | - | - | - |
| ACS | 0.5 - 1.0 | 1.0 - 24.0 | ACS, CCS | Abrantes et.al, 2021^2^ | 5 | 0.79 (0.56, 1.12) | High | - | - | - |
| ACS | 0.5 - 1.0 | 1.0 - 24.0 | CHD | Chen et.al, 2023^17^ | 5 | 0.70 (0.54, 0.90) | High | - | - | - |
| ACS | 0.5 - 1.0 | 1.0 - 24.0 | ACS, CCS | Akl et.al, 2024^25^ | 5 | 0.71 (0.58, 0.88) | High | - | - | - |
| ACS (new) | 0.5 - 1.8 | < 0.1 - 24.0 | CHD | Chen et.al, 2023^17^ | 5 | 0.72 (0.57, 0.92) | High | 3 | 0.53 (0.30, 0.95) | High |
| CHD | 0.5 - 1.2 | < 0.1 - 36.0 | CCS | Casula et.al, 2022^37^ | - | - | - | 3 | 0.61 (0.38, 0.98) | Moderate |
| CHD | 0.5 - 2.0 | 0.2 - 28.6 | CHD | Andreis et.al, 2021^7^ | 6 | 0.73 (0.60, 0.89) | High | - | - | - |
| Cardiac arrest | 0.5 - 2.0 | 1.0 - 3.0 | ACS | Younas et.al, 2024^19^ | 3 | 0.80 (0.33, 1.94) | High | - | - | - |
| MI | 0.5 - 1.0 | 1.0 - 24.0 | CHD | Sattar et.al, 2022^28^ | 3 | 0.79 (0.54, 1.15) | High | - | - | - |
| MI | 0.5 - 1.0 | 1.0 - 36.0 | CHD | Akl et.al, 2024^25^ | 4 | 0.73 (0.42, 1.26) | Moderate | - | - | - |
| MI | 0.5 - 1.0 | 6.0 - 12.0 | ACS | Niu et.al, 2022^27^ | 3 | 0.66 (0.33, 1.28) | High | - | - | - |
| MI | 0.5 - 1.2 | 1.0 - 28.6 | CHD | Andreis et.al, 2022^4^ | - | - | - | 3 | 0.66 (0.42, 1.05) | Moderate |
| MI | 0.5 - 1.8 | < 0.1 - 24.0 | ACS, CCS | Grajek et.al, 2021^9^ | 4 | 0.86 (0.66, 1.13) | High | 3 | 0.50 (0.23, 1.08) | High |
| MI | 0.5 - 1.8 | 0.2 - 36.0 | ACS, CCS | Ma et.al, 2022^5^ | 6 | 0.53 (0.29, 0.98) | Moderate | 3 | 0.63 (0.39, 1.02) | Low |
| MI | 0.5 - 2.0 | 0.2 - 19.6 | ACS | Zhou et.al, 2023^21^ | 4 | 0.88 (0.67, 1.16) | Moderate | - | - | - |
| MI (postoperative) | 0.5 - 1.0 | 1.0 - 7.1 | PCI | Wei et.al, 2023^23^ | 3 | 0.89 (0.67, 1.17) | High | - | - | - |
| MI (recurrent) | 0.5 - 1.2 | 1.0 - 36.0 | CHD | Bytyçi et.al, 2022^33^ | - | - | - | 3 | 0.69 (0.50, 0.96) | High |
| MI (recurrent) | 0.5 - 1.8 | < 0.1 - 36.0 | ACS, CCS | Wang et.al, 2021^30^ | - | - | - | 3 | 0.56 (0.31, 0.99) | High |
| MI (recurrent) | 0.5 - 2.0 | < 0.1 - 24.0 | ACS | Bao et.al, 2022^18^ | 6 | 0.63 (0.40, 1.01) | Moderate | - | - | - |
| MI (recurrent) | 0.5 - 2.0 | 1.0 - 6.0 | ACS | Younas et.al, 2024^19^ | 6 | 0.79 (0.59, 1.05) | Moderate | - | - | - |
| POAF | 0.5 - 1.0 | < 0.1 - 3.0 | Surgery (aortic, CABG) | Ge et.al, 2022^39^ | 3 | 0.60 (0.41, 0.87) | High | - | - | - |
| POAF | 0.5 - 1.0 | 0.2 - 1.0 | Surgery (aortic, CABG) | Wang et.al, 2022^1^ | 4 | 0.58 (0.38, 0.89) | High | - | - | - |
| POAF | 0.5 - 2.0 | 0.3 - out-hospital | Surgery (CABG) | Kirov et.al, 2024^40^ | 4 | 0.48 (0.34, 0.69) | High | - | - | - |
| POAF | 0.5 - 2.0 | < 3.0 | Surgery (aortic, CABG, MR) | Zhao et.al, 2022^41^ | 3 | 0.72 (0.50, 1.04) | High | - | - | - |
| POAF | 1.0 - 2.0 | In-hospital - 6.0 | Surgery (aortic, CABG) | Agarwal et.al, 2023^12^ | 5 | 0.64 (0.50, 0.82) | High | - | - | - |
| POAF (< 1 m) | 1.0 - 2.0 | In-hospital - 50.2 | Surgery (aortic, CABG, MR) | Agarwal et.al, 2023^12^ | 4 | 0.68 (0.51, 0.90) | High | - | - | - |
| **Hospitalization** |  |  |  |  |  |  |  |  |  |  |
| Hospitalization | 0.5 - 1.0 | 1.0 - 28.6 | ACS, CCS | Abrantes et.al, 2021^2^ | 6 | 0.55 (0.39, 0.78) | High | - | - | - |
| Hospitalization | 0.5 - 2.0 | 1.0 - 28.6 | CHD | Bytyçi et.al, 2022^33^ | 3 | 0.32 (0.11, 0.88) | High | - | - | - |
| Hospitalization urgency | 0.5 - 1.0 | 1.0 | ACS | Younas et.al, 2024^19^ | 3 | 0.45 (0.30, 0.67) | High | - | - | - |
| Rehospitalization | 0.5 - 1.0 | 1.0 - 22.0 | CHD | Shrestha, 2022^3^ | 4 | 0.64 (0.19, 2.07) | Moderate | - | - | - |
| **MACEs** |  |  |  |  |  |  |  |  |  |  |
| ACS, AF, mortality, revascularization and stroke | 0.5 - 2.0 | < 0.1 - 6.0 | Surgery (CABG, PCI) | Chen et.al, 2023^17^ | 4 | 0.63 (0.24, 1.62) | High | - | - | - |
| ACS, CHD, post-angioplasty and stroke | 0.5 - 1.0 | 1.0 - 36.0 | CHD | Verma et.al, 2015^10^ | 3 | 0.70 (0.26, 1.87) | High | - | - | - |
| Adverse cardiovascular events a | 0.5 - 2.0 | 1.0 - 6.0 | ACS | Younas et.al, 2024^19^ | 7 | 0.74 (0.56, 0.94) | High | - | - | - |
| All-cause mortality, cardiac arrest, ISR, MI, stent thrombosis and stroke | 0.5 - 1.0 | 1.0 - 12.0 | Surgery (PCI) | Wei et.al, 2023^23^ | 5 | 0.69 (0.57, 0.84) | High | - | - | - |
| All-cause mortality, cardiac arrest, ISR, MI, stent thrombosis and stroke | 0.5 - 2.0 | 0.2 - 6.0 | Surgery (PCI) | Wei et.al, 2023^23^ | 5 | 0.67 (0.45, 1.00) | High | - | - | - |
| All-cause mortality, CV mortality, MI and stroke | 0.5 - 1.0 | 6.0 - 24.0 | ACS, CCS | Abrantes et.al, 2021^2^ | 3 | 0.79 (0.61, 1.00) | High | - | - | - |
| All-cause mortality, CV mortality, MI and stroke | 0.5 - 1.0 | 6.0 - 24.0 | CHD | Xia et.al, 2021^13^ | 3 | 0.73 (0.60, 0.90) | High | - | - | - |
| All-cause mortality, CV mortality, recurrent MI and stroke | 0.5 - 1.2 | 1.0 - 36.0 | CHD | Bytyçi et.al, 2022^33^ | 4 | 0.73 (0.59, 0.89) | High | 3 | 0.61 (0.38, 0.98) | Moderate |
| All-cause mortality, CV mortality and stroke | 0.5 - 1.2 | 1.0 - 12.0 | CHD | Aw et.al, 2022^24^ | 6 | 0.70 (0.57, 0.85) | High | - | - | - |
| All-cause mortality, HF, MI, revascularization and stroke | 0.5 | 1.0 - 19.6 | ACS | Zhou et.al, 2023^21^ | 3 | 0.56 (0.47, 0.67) | High | - | - | - |
| All-cause mortality, HF, MI, revascularization and stroke | 0.5 - 2.0 | 0.2 - 19.6 | ACS | Zhou et.al, 2023^21^ | 5 | 0.56 (0.48, 0.67) | High | - | - | - |
| All-cause mortality, HF, MI, revascularization and stroke | 0.5 - 2.0 | < 12.0 | ACS | Zhou et.al, 2023^21^ | 3 | 0.58 (0.31, 1.07) | High | - | - | - |
| All-cause mortality, HF, MI, revascularization and stroke (≤ 3 d) | 0.5 - 2.0 | 0.2 - 19.6 | ACS | Zhou et.al, 2023^21^ | 5 | 0.59 (0.44, 0.79) | High | - | - | - |
| CV mortality, coronary revascularization, MI and stroke | 0.5 - 1.0 | 7.1 - 36.0 | ACS, CCS, surgery (PCI) | Xu et.al, 2022^15^ | 5 | 0.73 (0.59, 0.90) | High | - | - | - |
| CV mortality, coronary revascularization, MI and stroke | 0.5 - 1.0 | 7.1 - 36.0 | CHD, stroke | Fiolet et.al, 2024^29^ | - | - | - | 3 | 0.69 (0.54, 0.88) | Moderate |
| CV mortality, MI and stroke | 0.5 - 1.0 | 0.2 - 36.0 | ACS, CCS | Ma et.al, 2022^5^ | 5 | 0.57 (0.63, 0.92) | Moderate | - | - | - |
| CV mortality, MI and stroke | 0.5 - 1.0 | 1.0 | CHD | Akl et.al, 2024^25^ | 3 | 0.54 (0.13, 2.22) | High | - | - | - |
| CV mortality, MI and stroke | 0.5 - 1.0 | > 1.0 | CHD | Akl et.al, 2024^25^ | 3 | 0.72 (0.58, 0.89) | High | - | - | - |
| CV mortality, MI and stroke | 0.5 - 1.0 | 1.0 - 36.0 | ACS, CCS | Akl et.al, 2024^25^ | 4 | 0.74 (0.54, 1.01) | Moderate | - | - | - |
| CV mortality, MI,PCI, revascularization and stroke | 0.5 - 1.8 | < 0.1 - 24.0 | ACS, CCS | Grajek et.al, 2021^9^ | - | - | - | 3 | 0.66 (0.46, 0.95) | Low |
| Major adverse CV and cerebrovascular events | 0.5 - 2.0 | 0.2 - 28.6 | CHD | Andreis et.al, 2022^4^ | 8 | 0.71 (0.58, 0.86) | High | - | - | - |
| **Revascularization** |  |  |  |  |  |  |  |  |  |  |
| Revascularization | 0.5 - 1.0 | 1.0 - 36.0 | CHD | Xiang et.al, 2021^6^ | 3 | 0.50 (0.31, 0.80) | High | - | - | - |
| Revascularization | 0.5 - 1.0 | 6.0 - 24.0 | CHD | Chen et.al, 2023^17^ | 4 | 0.48 (0.31, 0.73) | High | - | - | - |
| Revascularization | 0.5 - 1.0 | 6.0 - 28.6 | CHD | Chen et.al, 2022^22^ | 3 | 0.62 (0.35, 1.20) | High | - | - | - |
| Revascularization | 0.5 - 1.0 | 6.0 - 36.0 | ACS, CCS | Grajek et.al, 2021^9^ | 3 | 0.48 (0.31, 0.74) | High | - | - | - |
| Revascularization | 0.5 - 1.0 | 7.1 - 36.0 | ACS, CCS, surgery (PCI) | Xu et.al, 2022^15^ | 3 | 0.33 (0.13, 0.80) | High | - | - | - |
| **Stroke** |  |  |  |  |  |  |  |  |  |  |
| Ischaemic stroke | 0.5 - 1.0 | 1.0 - 36.0 | CHD | Xiang et.al, 2021^6^ | 3 | 0.32 (0.13, 0.79) | High | - | - | - |
| Ischaemic stroke | 0.5 - 1.0 | 7.1 - 36.0 | CHD, stroke | Fiolet et.al, 2024^29^ | - | - | - | 3 | 0.77 (0.62, 0.96) | Moderate |
| Ischaemic stroke | 0.5 - 1.8 | 1.0 - 36.0 | CHD | Chen et.al, 2022^22^ | - | - | - | 3 | 0.65 (0.35, 1.19) | High |
| Non-cardio-embolic ischaemic stroke | 0.5 - 1.0 | 1.0 - 36.0 | CHD | Shrestha, 2022^3^ | 4 | 0.38 (0.15, 0.74) | High | - | - | - |
| Stroke | 0.5 - 1.0 | 0.2 - 36.0 | ACS, CCS | Ma et.al, 2022^5^ | 4 | 0.35 (0.16, 0.76) | Moderate | - | - | - |
| Stroke | 0.5 - 1.0 | 1.0 - 12.0 | ACS, AIS | Katsanos et.al, 2021^47^ | 3 | 0.54 (0.14, 2.06) | High | - | - | - |
| Stroke | 0.5 - 1.0 | 1.0 - 24.0 | ACS, CCS | Abrantes et.al, 2021^2^ | 4 | 0.32 (0.14, 0.70) | High | - | - | - |
| Stroke | 0.5 - 1.0 | 1.0 - 36.0 | ACS, CHD | Akl et.al, 2024^25^ | 3 | 0.31 (0.15, 0.61) | High | - | - | - |
| Stroke | 0.5 - 1.0 | 6.0 - 12.0 | Surgery (PCI) | Wei et.al, 2023^23^ | 3 | 0.32 (0.14, 0.73) | High | - | - | - |
| Stroke | 0.5 - 1.0 | 7.1 - 36.0 | ACS, CCS | Xu et.al, 2022^15^ | 3 | 0.91 (0.52, 1.60) | High | - | - | - |
| Stroke | 0.5 - 1.8 | < 0.1 - 24.0 | CHD | Chen et.al, 2023^17^ | - | - | - | 3 | 0.71 (0.39, 1.30) | High |
| Stroke | 0.5 - 2.0 | < 0.1 - 24.0 | ACS | Bao et.al, 2022^18^ | 5 | 0.37 (0.17, 0.78) | High | - | - | - |
| Stroke | 0.5 - 2.0 | 0.2 - 28.6 | CHD | Andreis et.al, 2022^4^ | 4 | 0.28 (0.13, 0.62) | High | 3 | 0.66 (0.365, 1.2) | High |
| Stroke | 0.5 - 2.0 | 1.0 - 3.0 | ACS | Younas et.al, 2024^19^ | 4 | 0.45 (0.17, 1.18) | High | - | - | - |
| Stroke | 0.5 - 2.0 | 1.0 - 28.6 | CHD | Bytyçi et.al, 2022^33^ | 4 | 0.27 (0.12, 0.59) | Moderate | - | - | - |
| **Other Outcomes** |  |  |  |  |  |  |  |  |  |  |
| CRP | 0.5 - 1.0 | 0.2 - 19.6 | ACS | Zhou et.al, 2023^21^ | 4 | 0.77 (0.46, 1.27) | Very low | - | - | - |
| CRP | 0.5 - 2.0 | 0.2 - 12.0 | ACS | Zhou et.al, 2023^21^ | 5 | 0.61 (0.38, 0.99) | Low | - | - | - |
| CRP | 0.5 - 2.0 | 0.2 - 19.6 | ACS | Zhou et.al, 2023^21^ | 8 | 0.62 (0.34, 1.13) | Low | - | - | - |
| Hs-CRP | 0.5 - 1.0 | 1.0 - 24.0 | ACS, CCS | Grajek et.al, 2021^9^ | 4 | 0.52 (0.22, 1.23) | Low | - | - | - |
| Hs-CRP | 0.5 - 2.0 | 0.2 - 6.0 | ACS | Younas et.al, 2024^19^ | 6 | 0.44 (0.21, 0.92) | Low | - | - | - |
| Hs-CRP | 0.5 - 2.0 | 0.2 - 24.0 | ACS | Diaz-Arocutipa et.al, 2021^20^ | 4 | 0.64 (0.37, 1.11) | Moderate | - | - | - |
| Leukocytes | 0.5 - 1.0 | 1.0 - 19.6 | ACS | Zhou et.al, 2023^21^ | 3 | 0.91 (0.78, 0.94) | High | - | - | - |
| LVEF | 0.5 - 2.0 | 0.2 - 12.0 | ACS | Zhou et.al, 2023^21^ | 3 | 1.85 (1.07, 3.18) | Moderate | - | - | - |
| Neutrophils | 0.5 - 1.0 | 0.2 - 19.6 | ACS | Zhou et.al, 2023^36^ | 4 | 0.88 (0.76, 1.01) | High | - | - | - |
| ACS, acute coronary syndrome; AF, atrial fibrillation; AIS, acute ischaemic stroke; CABG, coronary artery bypass grafting; CCS, chronic coronary syndromes; CHD, coronary heart disease; CRP, C-reactive protein; CV, cardiovascular; d, day; GRADE, Grading of Recommendations Assessment, Development, and Evaluation; HF, heart failure; Hs-CRP, high-sensitive C-reactive protein; ISR, in-stent restenosis; LVEF, left ventricular ejection fraction; m, month; MACEs, major adverse cardiac events; MI, myocardial infarction; MR, myocardial revascularization; No., number; PCI, percutaneous coronary intervention; POAF, postoperative atrial fibrillation; PROBE, Prospective, Randomized, Open-label, Blinded Endpoint; RCTs, randomised controlled trials. | | | | | | | | | | |
| ^a^ Adverse cardiovascular events: ACS, CV mortality, HF, MI, resuscitated cardiac arrest, stroke, UA, urgent hospitalization for angina and ventricular arrhythmias. | | | | | | | | | |  |

**Supplementary Table S13. Subgroup analysis according to the population of colchicine use on assessed associations**

| **Outcomes** | **Intervention  Dose (mg/d)** | **Intervention Duration (month)** | **Patients** | **Author, year,  reference** | **Acute or stable CAD** | | | **Post-operation** | | |
| --- | --- | --- | --- | --- | --- | --- | --- | --- | --- | --- |
|  |  |  |  |  | **No. of RCTs** | **Random effect size (95% CI)** | **GRADE** | **No. of RCTs** | **Random effect size (95% CI)** | **GRADE** |
|  |  |  |  |  |  |  |  |  |  |  |
| **Adverse events** |  |  |  |  |  |  |  |  |  |  |
| Adverse events | 0.5 - 1.0 | 0.2 - 1.0 | Surgery (aortic, CABG) | Wang et.al, 20221 | - | - | - | 3 | 2.61 (1.28, 5.49) | Low |
| Adverse events | 0.5 - 1.0 | 1.0 - 24.0 | ACS | Abrantes et.al, 20212 | 4 | 1.04 (0.81, 1.35) | Moderate | - | - | - |
| Diarrhea | 0.5 - 1.0 | 1.0 - 36.0 | CHD | Xiang et.al, 20216 | - | - | - | 3 | 2.10 (0.81, 5.40) | Low |
| Drug discontinuation | 0.5 | 7.1 - 28.6 | CHD | Andreis et.al, 20217 | 3 | 1.26 (0.87, 1.82) | Moderate | - | - | - |
| Drug discontinuation | 0.5 - 1.0 | 1.0 - 24.0 | ACS, CCS | Grajek et.al, 20219 | 5 | 1.46 (0.94, 2.29) | Moderate | 4 | 5.78 (0.72, 46.51) | Very low |
| Drug discontinuation | 0.5 - 1.0 | 6.0 - 24.0 | ACS, CCS | Grajek et.al, 20219 | 3 | 1.41 (0.89, 2.22) | Moderate | - | - | - |
| Drug discontinuation | 0.5 - 1.2 | 0.2 - 36.0 | CHD | Kofler et.al, 202111 | 6 | 1.50 (0.98, 2.30) | Low | 4 | 2.18 (1.09, 4.35) | Moderate |
| Drug discontinuation | 0.5 - 2.0 | 0.2 - 28.6 | CHD | Andreis et.al, 20217 | 4 | 1.53 (0.94, 2.50) | Low |  |  |  |
| Drug discontinuation | 1.0 - 2.0 | In-hospital - 1.0 | Surgery (aortic, CABG) | Agarwal et.al, 202312 | - | - | - | 3 | 1.33 (0.94, 1.90) | High |
| Gastrointestinal adverse events | 0.5 - 1.0 | 0.2 - 36.0 | CHD | Shrestha, 20223 | 5 | 1.43 (0.83, 2.47) | Moderate | 3 | 5.48 (0.45, 67.29) | Low |
| Gastrointestinal adverse events | 0.5 - 1.0 | 1.0 - 24.0 | ACS, CCS | Abrantes et.al, 20212 | 4 | 1.27 (0.93, 1.73) | High | 4 | 5.08 (1.17, 22.02) | Very low |
| Gastrointestinal adverse events | 0.5 - 1.0 | 6.0 - 24.0 | CHD | Xia et.al, 202113 | 3 | 1.15 (0.89, 1.49) | High | - | - | - |
| Gastrointestinal adverse events | 0.5 - 1.0 | 7.1 - 28.6 | CHD | Sen et.al, 202114 | 6 | 1.04 (0.89, 1.22) | High | - | - | - |
| Gastrointestinal adverse events | 0.5 - 1.0 | 7.1 - 28.6 | ACS, CCS, surgery (PCI) | Xu et.al, 202215 | 4 | 1.02 (0.92, 1.13) | High | - | - | - |
| Gastrointestinal adverse events | 0.5 - 1.8 | 0.2 - 36.0 | ACS, CCS | Ma et.al, 20225 | 10 | 1.64 (1.14, 2.36) | Low | 3 | 2.94 (1.84, 4.70) | Moderate |
| Gastrointestinal adverse events | 0.5 - 1.8 | 0.2 - 36.0 | CHD | Kofler et.al, 202111 | 7 | 1.50 (1.00, 2.26) | Low | 4 | 3.69 (2.20, 6.17) | High |
| Gastrointestinal adverse events | 0.5 - 1.8 | 0.2 - 36.0 | CHD | Tien et.al, 202116 | 4 | 4.19 (1.01, 17.48) | Very low | 4 | 4.08 (2.01, 8.27) | High |
| Gastrointestinal adverse events | 0.5 - 2.0 | < 0.1 - 24.0 | CHD | Chen et.al, 202317 | 7 | 1.87 (1.15, 3.05) | Low | 4 | 4.82 (1.12, 20.67) | Very low |
| Gastrointestinal adverse events | 0.5 - 2.0 | < 0.1 - 24.0 | ACS | Bao et.al, 202218 | 6 | 2.51 (1.24, 5.06) | Low | - | - | - |
| Gastrointestinal adverse events | 0.5 - 2.0 | 0.2 - 6.0 | ACS | Younas et.al, 202419 | 4 | 2.34 (0.85, 6.42) | Very low | 3 | 2.40 (0.72, 8.02) | Low |
| Gastrointestinal adverse events | 0.5 - 2.0 | 0.2 - 24.0 | ACS | Diaz-Arocutipa et.al, 202120 | 3 | 2.55 (0.75, 6.75) | Low | - | - | - |
| Gastrointestinal adverse events | 0.5 - 2.0 | 0.2 - 28.6 | CHD | Andreis et.al, 20217 | 5 | 1.16 (0.89, 1.52) | Low | - | - | - |
| Gastrointestinal adverse events | 0.5 - 2.0 | 0.2 - 19.6 | ACS | Zhou et.al, 202321 | - | - | - | 3 | 2.24 (0.70, 7.18) | Low |
| Gastrointestinal adverse events | 0.5 - 2.0 | 0.2 - 36.0 | CHD | Chen et.al, 202222 | 5 | 1.53 (0.94, 2.49) | Very low | 4 | 2.75 (1.82, 4.16) | Moderate |
| Gastrointestinal adverse events | 1.0 - 2.0 | In-hospital - 6.0 | Surgery (aortic, CABG) | Agarwal et.al, 202312 | - | - | - | 6 | 2.19 (1.39, 3.46) | Moderate |
| Gastrointestinal adverse events | 1.0 - 2.0 | In-hospital - 6.0 | Surgery (aortic, CABG) | Agarwal et.al, 202312 | - | - | - | 5 | 1.64 (1.24, 2.19) | High |
| Gastrointestinal adverse events (surgery < 1 m) | 1.0 - 2.0 | In-hospital - 50.2 | Surgery (aortic, CABG) | Agarwal et.al, 202312 | - | - | - | 3 | 2.04 (0.78, 5.31) | Low |
| Gastrointestinal adverse events (surgery ≥ 1 m) | 1.0 - 2.0 | 1.0 - 6.0 | Surgery (aortic, CABG) | Agarwal et.al, 202312 | - | - | - | 3 | 2.29 (1.41, 3.72) | High |
| Postoperative adverse events | 0.5 - 1.0 | 1.0 - 12.0 | PCI | Wei et.al, 202323 | 3 | 1.19 (0.93, 1.52) | Moderate | - | - | - |
| **All-cause and cause-specific mortality** |  |  |  |  |  |  |  |  |  |  |
| All-cause mortality | 0.5 - 1.0 | 1.0 - 28.6 | CHD | Akl et.al, 202425 | 3 | 1.80 (0.67, 4.83) | Low | - | - | - |
| All-cause mortality | 0.5 - 1.0 | 6.0 - 24.0 | CHD | Sattar et.al, 202228 | 3 | 1.73 (0.59, 5.09) | High | - | - | - |
| All-cause mortality | 0.5 - 1.0 | 7.1 - 36.0 | CHD, stroke | Fiolet et.al, 202429 | 4 | 1.08 (0.68, 1.72) | Moderate | - | - | - |
| All-cause mortality | 0.5 - 1.0 | 7.1 - 36.0 | CHD | Sen et.al, 202114 | 4 | 1.04 (0.61, 1.78) | Moderate | - | - | - |
| All-cause mortality | 0.5 - 1.2 | 7.1 - 36.0 | ACS, CCS, surgery (PCI) | Aw et.al, 202224 | - | - | - | 3 | 0.58 (0.13, 2.67) | Moderate |
| All-cause mortality | 0.5 - 1.8 | < 0.1 - 24.0 | ACS, CCS | Grajek et.al, 20219 | 5 | 1.23 (0.90, 1.69) | High | 3 | 0.63 (0.27, 1.46) | High |
| All-cause mortality | 0.5 - 1.8 | < 0.1 - 36.0 | ACS, CCS | Wang et.al, 202130 | 3 | 1.21 (0.76, 1.92) | Low | - | - | - |
| All-cause mortality | 0.5 - 1.8 | 0.2 - 36.0 | ACS, CCS | Ma et.al, 20225 | 5 | 1.04 (0.64, 1.69) | High | 5 | 0.78 (0.34, 1.76) | High |
| All-cause mortality | 0.5 - 1.8 | 0.2 - 36.0 | CHD | Kofler et.al, 202111 | 5 | 1.04 (0.63, 1.71) | High | 4 | 0.55 (0.21, 1.45) | High |
| All-cause mortality | 0.5 - 2.0 | < 0.1 | Surgery (PCI) | Fu etal, 202131 | - | - | - | 3 | 0.50 (0.18, 1.43) | High |
| All-cause mortality | 0.5 - 2.0 | < 0.1 - 24.0 | ACS | Bao et.al, 202218 | 4 | 2.39 (0.81, 7.06) | High | - | - | - |
| All-cause mortality | 0.5 - 2.0 | 0.2 - 6.0 | ACS | Younas et.al, 202419 | 4 | 1.04 (0.61, 1.76) | High | - | - | - |
| All-cause mortality | 0.5 - 2.0 | 0.2 - 24.0 | ACS | Diaz-Arocutipa et.al, 202120 | 3 | 3.06 (0.66, 14.10) | High | - | - | - |
| All-cause mortality | 0.5 - 2.0 | 0.2 - 36.0 | CHD | Xiang et.al, 20216 | 3 | 0.71 (0.59, 0.85) | Moderate | 4 | 0.54 (0.29, 1.02) | Moderate |
| All-cause mortality | 0.5 - 2.0 | 1.0 - 28.6 | CHD | Bytyçi et.al, 202233 | 4 | 1.34 (0.80, 2.25) | High | 3 | 0.76 (0.40, 1.47) | High |
| All-cause mortality (postoperative) | 0.5 - 1.0 | 6.0 - 12.0 | Surgery (PCI) | Wei et.al, 202323 | 3 | 1.62 (0.45, 5.89) | High | - | - | - |
| CV mortality | 0.5 - 1.0 | 0.2 - 36.0 | CHD | Kofler et.al, 202111 | 5 | 0.80 (0.49, 1.31) | High | - | - | - |
| CV mortality | 0.5 - 1.0 | 1.0 - 28.6 | CHD | Akl et.al, 202425 | 3 | 0.89 (0.51, 1.55) | Moderate | - | - | - |
| CV mortality | 0.5 - 1.0 | 1.0 - 36.0 | ACS, CCS | Ma et.al, 20225 | 3 | 0.86 (0.57, 1.29) | High | - | - | - |
| CV mortality | 0.5 - 1.0 | 1.0 - 36.0 | ACS, CCS | Grajek et.al, 20219 | 4 | 0.84 (0.50, 1.39) | High | - | - | - |
| CV mortality | 0.5 - 1.0 | 6.0 - 24.0 | ACS, CCS | Abrantes et.al, 20212 | 3 | 0.87 (0.50, 1.52) | High | - | - | - |
| CV mortality | 0.5 - 1.0 | 6.0 - 28.6 | CHD | Niu et.al, 202227 | 3 | 1.04 (0.53, 2.05) | Moderate | - | - | - |
| CV mortality | 0.5 - 1.0 | 6.0 - 36.0 | ACS, CCS | Grajek et.al, 20219 | 3 | 0.85 (0.49, 1.47) | High | - | - | - |
| CV mortality | 0.5 - 1.0 | 7.1 - 28.6 | ACS, CCS | Samuel et.al, 202135 | 3 | 0.82 (0.46, 1.37) | High | - | - | - |
| CV mortality | 0.5 - 1.0 | 7.1 - 36.0 | CHD | Sen et.al, 202114 | 4 | 0.66 (0.27, 1.58) | High | - | - | - |
| CV mortality | 0.5 - 1.0 | 7.1 - 36.0 | ACS, CCS, surgery (PCI) | Xu et.al, 202215 | 5 | 0.70 (0.55, 0.96) | Moderate | - | - | - |
| CV mortality | 0.5 - 1.0 | 7.1 - 36.0 | CHD, stroke | Fiolet et.al, 202429 | 4 | 0.81 (0.48, 1.35) | High | - | - | - |
| CV mortality | 0.5 - 1.8 | 1.0 - 36.0 | CHD | Chen et.al, 202222 | 4 | 0.79 (0.43, 1.44) | High | - | - | - |
| CV mortality | 0.5 - 2.0 | 0.2 - 24.0 | ACS | Bao et.al, 202218 | 3 | 2.03 (0.61, 6.82) | High | - | - | - |
| Non-CV mortality | 0.5 - 1.0 | 1.0 - 36.0 | ACS, CCS | Ma et.al, 20225 | 5 | 0.33 (0.93, 1.90) | High | - | - | - |
| Non-CV mortality | 0.5 - 1.0 | 7.1 - 28.6 | CHD | Andreis et.al, 20217 | 3 | 1.43 (0.94, 2.17) | High | - | - | - |
| Non-CV mortality | 0.5 - 1.0 | 7.1 - 36.0 | CHD | Sen et.al, 202114 | 4 | 1.49 (0.94, 2.36) | High | - | - | - |
| Non-CV mortality | 0.5 - 1.0 | 7.1 - 36.0 | CHD, stroke | Fiolet et.al, 202429 | 4 | 1.38 (0.99, 1.91) | High | - | - | - |
| Non-CV mortality | 0.5 - 1.0 | 7.1 - 36.0 | ACS, CCS | Xu et.al, 202215 | 4 | 1.34 (0.91, 1.99) | High | - | - | - |
| Non-CV mortality | 0.5 - 1.8 | 1.0 - 36.0 | CHD | Chen et.al, 202222 | 4 | 1.35 (0.90, 2.01) | High | - | - | - |
| **Cardiovascular disorders** |  |  |  |  |  |  |  |  |  |  |
| ACS | 0.5 - 1.0 | 1.0 - 22.0 | ACS | Ullah et.al, 202134 | 3 | 0.60 (0.23, 1.59) | High | - | - | - |
| ACS | 0.5 - 1.0 | 1.0 - 24.0 | ACS, CCS | Abrantes et.al, 20212 | 3 | 0.67 (0.51, 0.88) | High | 3 | 0.53 (0.21, 1.38) | Very low |
| ACS | 0.5 - 1.0 | 1.0 - 24.0 | CHD | Chen et.al, 202317 | 3 | 0.53 (0.24, 1.19) | High | - | - | - |
| ACS | 0.5 - 1.8 | 1.0 - 22.7 | ACS | Alberto et.al, 202136 | - | - | - | 3 | 0.56 (0.42, 0.76) | High |
| ACS (new) | 0.5 - 1.8 | < 0.1 - 24.0 | CHD | Chen et.al, 202317 | 4 | 0.68 (0.52, 0.88) | Moderate | 3 | 0.54 (0.28, 1.05) | Low |
| CHD | 0.5 - 2.0 | 0.2 - 28.6 | CHD | Andreis et.al, 20217 | 3 | 0.72 (0.63, 0.83) | High | 3 | 0.84 (0.53, 1.35) | High |
| CCS | 0.5 - 1.8 | 12.0 - 36.0 | CCS | Alberto et.al, 202136 | 3 | 0.49 (0.28, 0.86) | Low | - | - | - |
| MI | 0.5 - 1.0 | 1.0 - 24.0 | CHD | Sattar et.al, 202228 | - | - | - | 3 | 0.59 (0.23, 1.57) | Low |
| MI | 0.5 - 1.0 | 1.0 - 36.0 | ACS, AIS, surgery (PCI) | Masson et.al, 202026 | - | - | - | 3 | 0.45 (0.13, 1.60) | Low |
| MI | 0.5 - 1.0 | 6.0 - 28.6 | CHD | Niu et.al, 202227 | 3 | 0.63 (0.40, 0.99) | High | - | - | - |
| MI | 0.5 - 1.0 | 7.1 - 36.0 | ACS, CHD | Samuel et.al, 202135 | 4 | 0.62 (0.36, 0.88) | Low | - | - | - |
| MI | 0.5 - 1.0 | 7.1 - 36.0 | CHD | Al-Atta et.al, 202138 | 4 | 0.65 (0.46, 0.93) | Low | - | - | - |
| MI | 0.5 - 1.8 | < 0.1 - 24.0 | ACS, CCS | Grajek et.al, 20219 | 4 | 0.69 (0.53, 0.90) | High | 3 | 0.53 (0.18, 1.51) | Low |
| MI | 0.5 - 1.8 | 0.2 - 36.0 | ACS, CCS | Ma et.al, 20225 | 5 | 0.58 (0.39, 0.87) | Low | 3 | 0.58 (0.26, 1.31) | Moderate |
| MI | 0.5 - 1.8 | 1.0 - 36.0 | CHD | Chen et.al, 202222 | 5 | 0.72 (0.53, 0.98) | Moderate | - | - | - |
| MI (acute) | 0.5 - 1.0 | 1.0 - 36.0 | CHD | Xiang et.al, 20216 | - | - | - | 3 | 0.58 (0.23, 1.44) | Moderate |
| MI (recurrent) | 0.5 - 1.2 | 1.0 - 36.0 | CHD | Bytyçi et.al, 202233 | 4 | 0.73 (0.58, 0.93) | High | 3 | 0.65 (0.33, 1.30) | Moderate |
| MI (recurrent) | 0.5 - 1.8 | < 0.1 - 36.0 | ACS, CCS | Wang et.al, 202130 | 4 | 0.71 (0.51, 1.00) | Moderate | - | - | - |
| MI (recurrent) | 0.5 - 2.0 | < 0.1 - 24.0 | ACS | Bao et.al, 202218 | 5 | 0.61 (0.30, 1.24) | High | - | - | - |
| MI (recurrent) | 0.5 - 2.0 | 1.0 - 6.0 | ACS | Younas et.al, 202419 | 3 | 0.45 (0.16, 1.25) | High | 3 | 0.89 (0.68, 1.18) | High |
| POAF | 0.5 - 1.0 | < 0.1 - 3.0 | Surgery (aortic, CABG) | Ge et.al, 202239 | - | - | - | 5 | 0.59 (0.42, 0.84) | Moderate |
| POAF | 0.5 - 1.0 | 0.2 - 1.0 | Surgery (aortic, CABG) | Wang et.al, 20221 | - | - | - | 4 | 0.59 (0.39, 0.89) | Moderate |
| POAF | 0.5 - 2.0 | 0.3 - out-hospital | Surgery (CABG) | Kirov et.al, 202440 | - | - | - | 4 | 0.49 (0.34, 0.69) | High |
| POAF | 0.5 - 2.0 | < 3.0 | Surgery (aortic, CABG, MR) | Zhao et.al, 202241 | - | - | - | 4 | 0.73 (0.52, 1.02) | High |
| POAF | 1.0 - 2.0 | In-hospital - 6.0 | Surgery (aortic, CABG) | Agarwal et.al, 202312 | - | - | - | 7 | 0.73 (0.61, 0.87) | High |
| POAF (< 1 m) | 1.0 - 2.0 | In-hospital - 0.3 | Surgery (aortic, CABG, MR) | Zhao et.al, 202241 | - | - | - | 3 | 0.73 (0.51, 1.04) | High |
| POAF (< 1 m) | 1.0 - 2.0 | In-hospital - 50.2 | Surgery (aortic, CABG, MR) | Agarwal et.al, 202312 | - | - | - | 4 | 0.68 (0.51, 0.91) | High |
| POAF (≥ 1 m) | 1.0 - 2.0 | 1.0 - 6.0 | Surgery (aortic, CABG) | Agarwal et.al, 202312 | - | - | - | 3 | 0.74 (0.56, 0.99) | High |
| **Hospitalization** |  |  |  |  |  |  |  |  |  |  |
| Hospitalization | 0.5 - 1.0 | 1.0 - 28.6 | ACS, CCS | Abrantes et.al, 20212 | 3 | 0.54 (0.20, 1.50) | Moderate | - | - | - |
| **MACEs** |  |  |  |  |  |  |  |  |  |  |
| ACS, AF, mortality, revascularization and stroke | 0.5 - 2.0 | < 0.1 - 6.0 | Surgery (CABG, PCI) | Chen et.al, 202317 | 3 | 0.88 (0.52, 1.49) | High | - | - | - |
| Adverse cardiovascular events ^a^ | 0.5 - 2.0 | 1.0 - 6.0 | ACS | Younas et.al, 202419 | 4 | 0.64 (0.45, 0.89) | Moderate | 3 | 0.88 (0.69, 1.11) | High |
| All-cause mortality, cardiac arrest, ISR, MI, stent thrombosis and stroke | 0.5 - 2.0 | 0.2 - 6.0 | Surgery (PCI) | Wei et.al, 202323 | - | - | - | 5 | 0.68 (0.46, 1.00) | High |
| All-cause mortality, CV mortality, MI and stroke | 0.5 - 1.0 | 6.0 - 24.0 | ACS, CCS | Abrantes et.al, 20212 | - | - | - | 3 | 0.54 (0.23, 1.26) | Very low |
| All-cause mortality, CV mortality, MI and stroke | 0.5 - 1.0 | 6.0 - 24.0 | CHD | Xia et.al, 202113 | 3 | 0.70 (0.59, 0.83) | High | - | - | - |
| All-cause mortality, CV mortality, recurrent MI and stroke | 0.5 - 1.2 | 1.0 - 36.0 | CHD | Bytyçi et.al, 202233 | 4 | 0.71 (0.60, 0.83) | High | 3 | 0.55 (0.26, 1.14) | Low |
| All-cause mortality, CV mortality and stroke | 0.5 - 1.2 | 1.0 - 12.0 | CHD | Aw et.al, 202224 | 3 | 0.73 (0.60, 0.90) | Moderate | 5 | 0.72 (0.54, 0.95) | Moderate |
| All-cause mortality, HF, MI, revascularization and stroke | 0.5 - 2.0 | 0.2 - 19.6 | ACS | Zhou et.al, 202321 | - | - | - | 3 | 0.69 (0.46, 1.01) | High |
| All-cause mortality, HF, MI, revascularization and stroke (≤ 3 d) | 0.5 - 2.0 | 0.2 - 19.6 | ACS | Zhou et.al, 202321 | - | - | - | 3 | 0.68 (0.46, 1.01) | High |
| All-cause mortality, recurrent MI and stroke | 0.5 - 1.8 | < 0.1 - 36.0 | ACS, CCS | Wang et.al, 202130 | 3 | 0.82 (0.70, 0.95) | Low | - | - | - |
| All-cause mortality, recurrent MI, revascularization and stroke | 0.5 | 12.0 - 36.0 | ACS, CCS | Wang et.al, 202130 | 3 | 0.74 (0.61, 0.89) | Low | - | - | - |
| CV mortality, coronary revascularization, MI and stroke | 0.5 | 7.1 - 36.0 | CHD | Condello et.al, 202143 | 3 | 0.73 (0.50, 1.06) | Low | - | - | - |
| CV mortality, coronary revascularization, MI and stroke | 0.5 | 7.1 - 36.0 | ACS, CCS, surgery (PCI) | Xu et.al, 202215 | 4 | 0.63 (0.48, 0.82) | Low | - | - | - |
| CV mortality, coronary revascularization, MI and stroke | 0.5 - 1.0 | 12.0 - 36.0 | CHD | Masson et.al, 202144 | 4 | 0.70 (0.50, 0.98) | Low | - | - | - |
| CV mortality, coronary revascularization, MI and stroke | 0.5 - 1.0 | 7.1 - 28.6 | ACS, CCS | Samuel et.al, 202135 | 3 | 0.71 (0.61, 0.82) | Moderate | - | - | - |
| CV mortality, coronary revascularization, MI and stroke | 0.5 - 1.0 | 7.1 - 36.0 | ACS, CCS, surgery (PCI) | Xu et.al, 202215 | 6 | 0.63 (0.49, 0.80) | Moderate | - | - | - |
| CV mortality, coronary revascularization, MI and stroke | 0.5 - 1.0 | 7.1 - 36.0 | CHD, stroke | Fiolet et.al, 202429 | 4 | 0.67 (0.56, 0.82) | Moderate | - | - | - |
| CV mortality, coronary revascularization, MI and stroke | 0.5 - 1.0 | 7.1 - 36.0 | CHD | Al-Atta et.al, 202138 | 4 | 0.66 (0.52, 0.83) | Low | - | - | - |
| CV mortality, MI and stroke | ≤ 0.5 | 1.0 - 28.6 | CHD | Akl et.al, 202425 | 3 | 0.67 (0.56, 0.80) | Moderate | - | - | - |
| CV mortality, MI and stroke | 0.5 - 1.0 | 0.2 - 36.0 | ACS, CCS | Ma et.al, 20225 | 6 | 0.53 (0.37, 0.76) | Low | - | - | - |
| CV mortality, MI and stroke | 0.5 - 1.0 | > 1.0 | CHD | Akl et.al, 202425 | 3 | 0.68 (0.57, 0.81) | Moderate | - | - | - |
| CV mortality, MI and stroke | 0.5 - 1.0 | 1.0 - 36.0 | ACS, CCS | Akl et.al, 202425 | 3 | 0.70 (0.56, 0.87) | High | - | - | - |
| CV mortality, MI,PCI, revascularization and stroke | 0.5 - 1.8 | < 0.1 - 24.0 | ACS, CCS | Grajek et.al, 20219 | 3 | 0.77 (0.61, 0.96) | Moderate | - | - | - |
| Major CV events b | 0.5 - 1.0 | 7.1 - 36.0 | CHD with diabetes | Kuzemczak et.al, 202146 | 4 | 0.70 (0.50, 0.96) | Moderate | - | - | - |
| **Revascularization** |  |  |  |  |  |  |  |  |  |  |
| Revascularization | 0.5 - 1.0 | 1.0 - 36.0 | ACS, CCS | Grajek et.al, 20219 | 4 | 0.65 (0.41, 1.03) | High | - | - | - |
| Revascularization | 0.5 - 1.0 | 6.0 - 24.0 | Atherosclerosis | Fiolet et.al, 202145 | 3 | 0.73 (0.53, 1.00) | High | - | - | - |
| Revascularization | 0.5 - 1.0 | 6.0 - 24.0 | CHD | Chen et.al, 202317 | 4 | 0.72 (0.57, 0.91) | High | - | - | - |
| Revascularization | 0.5 - 1.0 | 6.0 - 36.0 | ACS, CCS | Grajek et.al, 20219 | 3 | 0.65 (0.38, 1.11) | High | - | - | - |
| Revascularization | 0.5 - 1.0 | 7.1 - 36.0 | ACS, CCS, surgery (PCI) | Xu et.al, 202215 | 4 | 0.48 (0.29, 0.80) | Moderate | - | - | - |
| Revascularization | 0.5 - 1.0 | 12.0 - 36.0 | ACS, CCS | Wang et.al, 202130 | 3 | 0.58 (0.37, 0.91) | Low | - | - | - |
| Restenosis after PCI | 1.0 - 1.2 | 6 | Surgery (PCI) | Tien et.al, 202116 | 3 | 0.75 (0.39, 1.45) | High | 6 | 0.55 (0.37, 0.82) | Moderate |
| **Stroke** |  |  |  |  |  |  |  |  |  |  |
| Ischaemic stroke | 0.5 - 1.0 | 7.1 - 36.0 | ACS, CCS | Samuel et.al, 202135 | 4 | 0.38 (0.13, 0.63) | High | - | - | - |
| Ischaemic stroke | 0.5 - 1.0 | 7.1 - 36.0 | CHD, stroke | Fiolet et.al, 202429 | 4 | 0.50 (0.30, 0.82) | Moderate | - | - | - |
| Ischaemic stroke | 0.5 - 1.8 | 1.0 - 36.0 | CHD | Chen et.al, 202222 | 7 | 0.66 (0.50, 0.86) | Moderate | - | - | - |
| Stroke | 0.5 - 1.0 | 0.2 - 36.0 | ACS, CCS | Ma et.al, 20225 | 5 | 0.50 (0.30, 0.83) | Moderate | - | - | - |
| Stroke | 0.5 - 1.0 | 1.0 - 24.0 | ACS, CCS | Abrantes et.al, 20212 | 3 | 0.63 (0.35, 1.13) | High | - | - | - |
| Stroke | 0.5 - 1.0 | 1.0 - 36.0 | ACS, AIS, CCS | Masson et.al, 202026 | - | - | - | 3 | 0.26 (0.11, 0.62) | High |
| Stroke | 0.5 - 1.0 | 12.0 - 36.0 | ACS, CCS | Grajek et.al, 20219 | 3 | 0.64 (0.36, 1.15) | High | - | - | - |
| Stroke | 0.5 - 1.2 | 7.1 - 36.0 | CHD | Aw et.al, 202224 | 4 | 0.46 (0.27, 0.77) | Moderate | - | - | - |
| Stroke | 0.5 - 1.8 | < 0.1 - 24.0 | CHD | Chen et.al, 202317 | 4 | 0.75 (0.42, 1.33) | High | - | - | - |
| Stroke | 0.5 - 2.0 | < 0.1 - 24.0 | ACS | Bao et.al, 202218 | 3 | 0.70 (0.21, 2.37) | High | - | - | - |
| Stroke | 0.5 - 2.0 | 1.0 - 28.6 | CHD | Bytyçi et.al, 202233 | 4 | 0.62 (0.35, 1.09) | High | - | - | - |
| **Other Outcomes** |  |  |  |  |  |  |  |  |  |  |
| Cancer | 0.5 - 1.0 | 7.1 - 28.6 | CHD | Sen et.al, 202114 | 3 | 0.98 (0.79, 1.21) | High | - | - | - |
| CRP | 0.5 - 1.0 | 0.2 - 19.6 | ACS | Zhou et.al, 202321 | 3 | 1.26 (0.49, 3.23) | Very low | - | - | - |
| CRP | 0.5 - 2.0 | 0.2 - 19.6 | ACS | Zhou et.al, 202321 | 5 | 0.59 (0.22, 1.58) | Low | 3 | 0.67 (0.44, 1.04) | Low |
| Hs-CRP | 0.5 - 1.0 | 1.0 - 24.0 | ACS, CCS | Grajek et.al, 20219 | 5 | 0.49 (0.25, 0.94) | Very low | - | - | - |
| Hs-CRP | 0.5 - 2.0 | 0.2 - 6.0 | ACS | Younas et.al, 202419 | 4 | 0.33 (0.12, 0.87) | Very low | - | - | - |
| Hs-CRP | 0.5 - 2.0 | 0.2 - 24.0 | ACS | Diaz-Arocutipa et.al, 202120 | 3 | 0.54 (0.35, 0.86) | High | - | - | - |
| Leukocytes | 0.5 - 1.0 | 1.0 - 19.6 | ACS | Zhou et.al, 202321 | 3 | 0.41 (0.13, 1.30) | High | - | - | - |
| Neutrophils | 0.5 - 1.0 | 0.2 - 19.6 | ACS | Zhou et.al, 202321 | 4 | 0.39 (0.15, 0.98) | High | - | - | - |
| ACS, acute coronary syndrome; AF, atrial fibrillation; AIS, acute ischaemic stroke; CABG, coronary artery bypass grafting; CAD, coronary artery disease; CCS, chronic coronary syndromes; CHD, coronary heart disease; CI, confidence interval; CRP, C-reactive protein; CV, cardiovascular; d, day; GRADE, Grading of Recommendations Assessment, Development, and Evaluation; HF, heart failure; Hs-CRP, high-sensitive C-reactive protein; ISR, in-stent re-stenosis; m, month; MACEs, major adverse cardiac events; MI, myocardial infarction; MR, myocardial revascularization; NA, not available; No., number; PCI, percutaneous coronary intervention; POAF, postoperative atrial fibrillation; RCTs, randomised controlled trials. a Adverse cardiovascular events: ACS, CV mortality, HF, MI, resuscitated cardiac arrest, stroke, UA, urgent hospitalization for angina and ventricular arrhythmias. b Major CV events: ACS, out-of-hospital cardiac arrest, CV mortality, resuscitated cardiac arrest, MI, stroke, or urgent hospitalization for angina, leading to coronary revascularization, ischaemic stroke, or ischemia-driven coronary revascularization. | | | | | | | | | | |


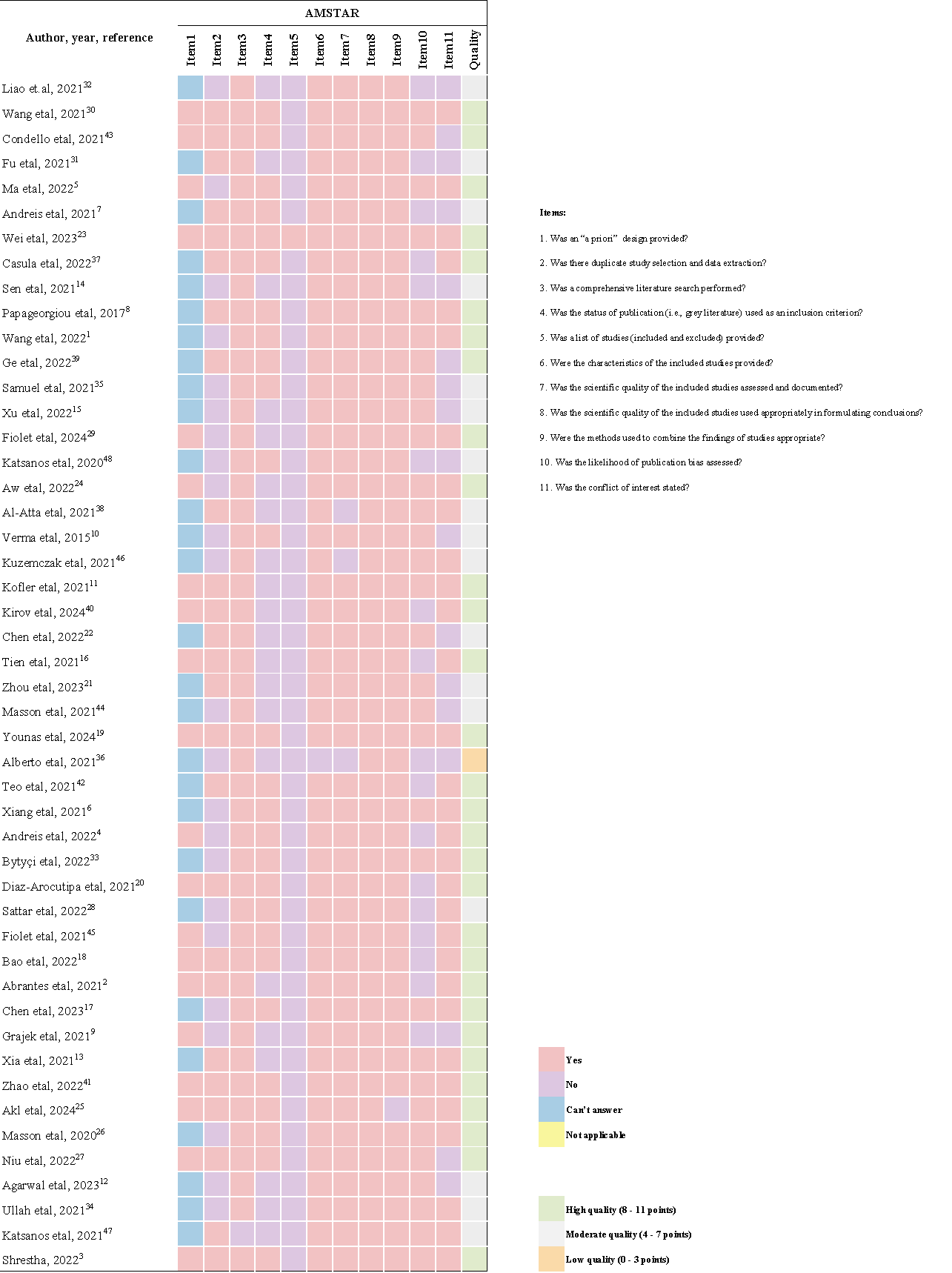


**Supplementary Figure S1. A Measurement Tool to Assess Systematic Reviews (AMSTAR) scores for each published meta-analysis**

| **References** |
| --- |

1. Wang X, Peng X, Li Y, et al. Colchicine for Prevention of Post-Cardiac Surgery and Post-Pulmonary Vein Isolation Atrial Fibrillation: A Meta-Analysis. *Reviews in cardiovascular medicine* 2022; **23**(12): 387.

2. Abrantes AM, Nogueira-Garcia B, Alves M, et al. Low-Dose Colchicine in Coronary Artery Disease　- Systematic Review and Meta-Analysis. *Circulation reports* 2021; **3**(8): 457-64.

3. Shrestha DB, Budhathoki P, Sedhai Y, et al. Colchicine for Patients With Coronary Artery Disease: A Systematic Review and Meta-analysis. *Journal of cardiovascular pharmacology* 2022; **79**(4): 420-30.

4. Andreis A, Imazio M, Piroli F, et al. Efficacy and safety of colchicine for the prevention of major cardiovascular and cerebrovascular events in patients with coronary artery disease: a systematic review and meta-analysis on 12 869 patients. *European journal of preventive cardiology* 2022; **28**(17): 1916-25.

5. Ma Z, Chen J, Jin K, Chen X. Colchicine and coronary heart disease risks: A meta-analysis of randomized controlled clinical trials. *Frontiers in cardiovascular medicine* 2022; **9**: 947959.

6. Xiang Z, Yang J, Yang J, et al. Efficacy and safety of colchicine for secondary prevention of coronary heart disease: a systematic review and meta-analysis. *Internal and emergency medicine* 2021; **16**(2): 487-96.

7. Andreis A, Imazio M, Casula M, Avondo S, De Ferrari GM. Colchicine efficacy and safety for the treatment of cardiovascular diseases. *Internal and emergency medicine* 2021; **16**(6): 1691-700.

8. Papageorgiou N, Briasoulis A, Lazaros G, Imazio M, Tousoulis D. Colchicine for prevention and treatment of cardiac diseases: A meta-analysis. *Cardiovascular therapeutics* 2017; **35**(1): 10-8.

9. Grajek S, Michalak M, Urbanowicz T, Olasińska-Wiśniewska A. A Meta-Analysis Evaluating the Colchicine Therapy in Patients With Coronary Artery Disease. *Frontiers in cardiovascular medicine* 2021; **8**: 740896.

10. Verma S, Eikelboom JW, Nidorf SM, et al. Colchicine in cardiac disease: a systematic review and meta-analysis of randomised controlled trials. *BMC cardiovascular disorders* 2015; **15**: 96.

11. Kofler T, Kurmann R, Lehnick D, et al. Colchicine in Patients With Coronary Artery Disease: A Systematic Review and Meta-Analysis of Randomized Trials. *Journal of the American Heart Association* 2021; **10**(16): e021198.

12. Agarwal S, Beard CW, Khosla J, et al. Safety and efficacy of colchicine for the prevention of post-operative atrial fibrillation in patients undergoing cardiac surgery: a meta-analysis of randomised controlled trials. *Europace : European pacing, arrhythmias, and cardiac electrophysiology : journal of the working groups on cardiac pacing, arrhythmias, and cardiac cellular electrophysiology of the European Society of Cardiology* 2023; **25**(7).

13. Xia M, Yang X, Qian C. Meta-analysis Evaluating the Utility of Colchicine in Secondary Prevention of Coronary Artery Disease. *The American journal of cardiology* 2021; **140**: 33-8.

14. Şen S, Karahan E, Büyükulaş C, Polat YO, Üresin AY. Colchicine for cardiovascular therapy: A drug interaction perspective and a safety meta-analysis. *Anatolian journal of cardiology* 2021; **25**(11): 753-61.

15. Xu H, Mao L, Liu H, Lin Z, Zhang Y, Yang J. Colchicine for Secondary Prevention of Coronary Artery Disease: A Meta-Analysis of Randomised Controlled Trials. *Heart, lung & circulation* 2022; **31**(5): 685-95.

16. Tien YY, Huang HK, Shih MC, Tu YK. Drug repurposing? Cardiovascular effect of colchicine on patients with coronary artery disease: A systematic review and meta-analysis. *Journal of cardiology* 2021; **77**(6): 576-82.

17. Chen T, Liu G, Yu B. A meta-analysis evaluating efficacy and safety of colchicine for prevention of major cardiovascular events in patients with coronary artery disease. *Clinical research in cardiology : official journal of the German Cardiac Society* 2023; **112**(11): 1487-505.

18. Bao YL, Gu LF, Du C, Wang YX, Wang LS. Evaluating the Utility of Colchicine in Acute Coronary Syndrome: A Systematic Review and Meta-Analysis. *Journal of cardiovascular pharmacology* 2022; **80**(5): 639-47.

19. Younas A, Awan Z, Khan T, et al. The effect of colchicine on myocardial infarction: An updated systematic review and meta-analysis of randomised controlled trials. *Current problems in cardiology* 2025; **50**(1): 102878.

20. Diaz-Arocutipa C, Benites-Meza JK, Chambergo-Michilot D, et al. Efficacy and Safety of Colchicine in Post-acute Myocardial Infarction Patients: A Systematic Review and Meta-Analysis of randomised controlled trials. *Frontiers in cardiovascular medicine* 2021; **8**: 676771.

21. Zhou Y, Liu Y, Zeng R, Qiu W, Zhao Y, Zhou Y. Early long-term low-dosage colchicine and major adverse cardiovascular events in patients with acute myocardial infarction: a systematic review and meta-analysis. *Frontiers in cardiovascular medicine* 2023; **10**: 1194605.

22. Chen Y, Zhang H, Chen Y, et al. Colchicine may become a new cornerstone therapy for coronary artery disease: a meta-analysis of randomised controlled trials. *Clinical rheumatology* 2022; **41**(6): 1873-87.

23. Wei ZY, Lai JY, Li YT, et al. Colchicine efficacy comparison at varying time points in the peri-operative period for coronary artery disease: a systematic review and meta-analysis of randomised controlled trials. *Frontiers in cardiovascular medicine* 2023; **10**: 1156980.

24. Aw KL, Koh A, Lee HL, Kudzinskas A, De Palma R. Colchicine for symptomatic coronary artery disease after percutaneous coronary intervention. *Open heart* 2022; **9**(1).

25. Akl E, Sahami N, Labos C, et al. Meta-Analysis of Randomized Trials: Efficacy and Safety of Colchicine for Secondary Prevention of Cardiovascular Disease. *Journal of interventional cardiology* 2024; **2024**: 8646351.

26. Masson W, Lobo M, Molinero G, Masson G, Lavalle-Cobo A. Role of Colchicine in Stroke Prevention: An Updated Meta-Analysis. *Journal of stroke and cerebrovascular diseases : the official journal of National Stroke Association* 2020; **29**(5): 104756.

27. Niu Y, Bai N, Ma Y, Zhong PY, Shang YS, Wang ZL. Safety and efficacy of anti-inflammatory therapy in patients with coronary artery disease: a systematic review and meta-analysis. *BMC cardiovascular disorders* 2022; **22**(1): 84.

28. Sattar L, Memon RA, Ashfaq F, et al. Efficacy and Safety of Colchicine in Prevention of Secondary Cardiovascular Outcomes Among Patients With Coronary Vessel Disease: A Meta-Analysis. *Cureus* 2022; **14**(7): e26680.

29. Fiolet ATL, Poorthuis MHF, Opstal TSJ, et al. Colchicine for secondary prevention of ischaemic stroke and atherosclerotic events: a meta-analysis of randomised trials. *EClinicalMedicine* 2024; **76**: 102835.

30. Wang H, Jiang M, Li X, et al. Anti-inflammatory Therapies for Coronary Heart Disease: A Systematic Review and Meta-Analysis. *Frontiers in cardiovascular medicine* 2021; **8**: 726341.

31. Fu C, Wang B. Colchicine administration for percutaneous coronary intervention: A meta-analysis of randomised controlled trials. *Am J Emerg Med* 2021; **46**: 121-5.

32. Chunfeng L, Ping L, Yun Z, Di L, Qi W. Colchicine for Coronary Heart Disease: A Meta-Analysis of randomised controlled trials. *Heart Surg Forum* 2021; **24**(5): E863-e7.

33. Bytyçi I, Bajraktari G, Penson PE, Henein MY, Banach M. Efficacy and safety of colchicine in patients with coronary artery disease: A systematic review and meta-analysis of randomised controlled trials. *British journal of clinical pharmacology* 2022; **88**(4): 1520-8.

34. Ullah W, Haq S, Zahid S, et al. Safety and Efficacy of Colchicine in Patients with Stable CAD and ACS: A Systematic Review and Meta-analysis. *American journal of cardiovascular drugs : drugs, devices, and other interventions* 2021; **21**(6): 659-68.

35. Samuel M, Tardif JC, Bouabdallaoui N, et al. Colchicine for Secondary Prevention of Cardiovascular Disease: A Systematic Review and Meta-analysis of randomised controlled trials. *The Canadian journal of cardiology* 2021; **37**(5): 776-85.

36. Aimo A, Pascual Figal DA, Bayes-Genis A, Emdin M, Georgiopoulos G. Effect of low-dose colchicine in acute and chronic coronary syndromes: A systematic review and meta-analysis. *European journal of clinical investigation* 2021; **51**(4): e13464.

37. Casula M, Andreis A, Avondo S, Vaira MP, Imazio M. Colchicine for cardiovascular medicine: a systematic review and meta-analysis. *Future Cardiol* 2022; **18**(8): 647-59.

38. Al-Atta A, Kuzemczak M, Alkhalil M. Colchicine for the prevention of ischaemic stroke: An updated meta-analysis of randomized clinical trials. *Brain circulation* 2021; **7**(3): 187-93.

39. Ge P, Fu Y, Su Q, et al. Colchicine for prevention of post-operative atrial fibrillation: Meta-analysis of randomised controlled trials. *Frontiers in cardiovascular medicine* 2022; **9**: 1032116.

40. Kirov H, Caldonazo T, Runkel A, et al. Colchicine in Patients With Coronary Disease Who Underwent Coronary Artery Bypass Surgery: A Meta-Analysis of randomised controlled trials. *The American journal of cardiology* 2024; **231**: 48-54.

41. Zhao H, Chen Y, Mao M, Yang J, Chang J. A meta-analysis of colchicine in prevention of atrial fibrillation following cardiothoracic surgery or cardiac intervention. *Journal of cardiothoracic surgery* 2022; **17**(1): 224.

42. Teo YN, Teo YH, Syn NL, et al. Effects of Colchicine on Cardiovascular Outcomes in Patients with Coronary Artery Disease: A Systematic Review and One-Stage and Two-Stage Meta-Analysis of Randomized-Controlled Trials. *High blood pressure & cardiovascular prevention : the official journal of the Italian Society of Hypertension* 2021; **28**(4): 343-54.

43. Condello F, Sturla M, Reimers B, et al. Association Between Colchicine Treatment and Clinical Outcomes in Patients with Coronary Artery Disease: Systematic Review and Meta-analysis. *Eur Cardiol* 2021; **16**: e39.

44. Masson W, Lobo M, Barbagelata L, Lavalle-Cobo A, Molinero G. Effect of anti-inflammatory therapy on major cardiovascular events in patients with diabetes: A meta-analysis. *Diabetes & metabolic syndrome* 2021; **15**(4): 102164.

45. Fiolet ATL, Opstal TSJ, Mosterd A, et al. Efficacy and safety of low-dose colchicine in patients with coronary disease: a systematic review and meta-analysis of randomized trials. *European heart journal* 2021; **42**(28): 2765-75.

46. Kuzemczak M, Ibrahem A, Alkhalil M. Colchicine in Patients with Coronary Artery Disease with or Without Diabetes Mellitus: A Meta-analysis of Randomized Clinical Trials. *Clin Drug Investig* 2021; **41**(8): 667-74.

47. Katsanos AH, Palaiodimou L, Price C, et al. An Updated Meta-Analysis of RCTs of Colchicine for Stroke Prevention in Patients with Coronary Artery Disease. *Journal of clinical medicine* 2021; **10**(14).

48. Katsanos AH, Palaiodimou L, Price C, et al. Colchicine for stroke prevention in patients with coronary artery disease: a systematic review and meta-analysis. *European journal of neurology* 2020; **27**(6): 1035-8.

49. Boczar KE, Shin S, Bezzina KA, et al. Examining anti-inflammatory therapies in the prevention of cardiovascular events: protocol for a systematic review and network meta-analysis of randomised controlled trials. *BMJ open* 2022; **12**(6): e062702.

50. Fiolet ATL, Nidorf SM, Mosterd A, Cornel JH. Colchicine in Stable Coronary Artery Disease. *Clinical therapeutics* 2019; **41**(1): 30-40.

51. Kundu M, Ghosh S, Shree A, et al. A systematic review on the use of Colchicine in Hemorrhagic Stroke. *World Neurosurg X* 2024; **23**: 100314.

52. Madanchi M, Young M, Tersalvi G, et al. The impact of colchicine on patients with acute and chronic coronary artery disease. *European journal of internal medicine* 2024; **125**: 1-9.

53. McKnight AH, Katzenberger DR, Britnell SR. Colchicine in Acute Coronary Syndrome: A Systematic Review. *Ann Pharmacother* 2021; **55**(2): 187-97.

54. Schattner A. Colchicine - new horizons for an ancient drug. Review based on the highest hierarchy of evidence. *Eur J Intern Med* 2022; **96**: 34-41.
